# Analysis of the Chemical Profile of the Essential Oil from Leaves and Cones of Four Varieties of *Humulus lupulus* Cultivated in a Subtropical Climate Under Edaphoclimatic Conditions

**DOI:** 10.3390/molecules31203605

**Published:** 2026-10-10

**Authors:** Gabriela Catuzo Canônico Silva, José Junior Severino, Fabiana Pereira Alves da Silva, Ricardo Furtado Silva, Janaine Gilliet Campanholi, Cleuni Fernanda Thomas Sulzbach, Judson Ricardo Ribeiro da Silva, Luciana Kazue Otutumi, Maria Graciela Iecher Faria Nunes, Juliana Silveira do Valle, Daniela Dib Gonçalves, João Paulo Francisco, José Eduardo Gonçalves, Antonio Laverde Junior, Zilda Cristiani Gazim

**Affiliations:** 1 Graduate Program in Biotechnology Applied to Agriculture, Universidade Paranaense, Umuarama 87502-210, Brazil; gabriela.canonico@edu.unipar.br (G.C.C.S.); jose.258613@edu.unipar.br (J.J.S.); fabiana.alves@edu.unipar.br (F.P.A.d.S.); ricardofurtado@unipar.br (R.F.S.); gracielaiecher@prof.unipar.br (M.G.I.F.N.); jsvalle@prof.unipar.br (J.S.d.V.); 2 Graduate Program in Animal Science with Emphasis on Bioactive Products, Universidade Paranaense, Umuarama 87502-210, Brazil; janaine.gilliet@edu.unipar.br (J.G.C.); otutumi@prof.unipar.br (L.K.O.); danieladib@prof.unipar.br (D.D.G.); 3 Graduate Program in Medicinal and Phytotherapeutic Plants in Primary Care, Universidade Paranaense, Umuarama 87502-210, Brazil; cleuni210472@edu.unipar.br; 4 Graduate Program in Clean Technologies, Cesumar Institute of Science, Technology and Innovation, UniCesumar, Maringá 87050-390, Brazil; judson.ribeiro@gmail.com (J.R.R.d.S.); jose.goncalves@unicesumar.edu.br (J.E.G.); 5 Department of Agricultural Sciences, Universidade Estadual de Maringá, Campus Umuarama, Umuarama 87020-900, Brazil; jpfrancisco2@uem.br; 6 Laboratory of Organic Chemistry and Biomaterials, Department of Chemistry, Universidade Tecnológica Federal do Paraná, Campus Londrina, Londrina 86036-700, Brazil; aljunior@utfpr.edu.br

**Keywords:** caryophyllene, humulene, Arenito Caiuá, varieties, hops

## Abstract

Cultivating *Humulus lupulus* L. under subtropical conditions is challenging because hops are adapted to temperate climates, where environmental factors influence plant development and secondary metabolism. This study evaluated the essential oil yield and chemical composition of cones and leaves from four hop varieties (Cascade, Columbus, Comet, and Nugget) cultivated under subtropical conditions in northwestern Paraná, Brazil. Essential oils were obtained by hydrodistillation and analyzed by GC–MS. Chemical variability was investigated using Pearson correlation, principal component analysis (PCA), and hierarchical cluster analysis (HCA). Cascade and Columbus showed better agronomic performance, while Nugget and Comet produced the highest essential oil yields in cones (1.532% and 0.695%, respectively). In leaves, Nugget, Cascade, and Comet exhibited the highest yields. Sesquiterpene hydrocarbons predominated in most samples, with α-humulene and β-caryophyllene as major constituents in both organs. Oxygenated sesquiterpenes, including caryophyllene oxide and humulene oxides, were also identified in the essential oils. Phytol was relatively abundant in the leaves of Cascade, Columbus, and Nugget, demonstrating differences between vegetative and reproductive tissues. PCA revealed distinct chemical patterns among varieties and harvest periods, emphasizing the influence of genotype and seasonal conditions. These results demonstrate the potential of hop cultivation under subtropical conditions and highlight leaves as a promising source of volatile compounds for further valorization and industrial applications.

## 1. Introduction

*Humulus lupulus*, commonly known as hops, is cultivated primarily for its cones, which are used in the brewing, pharmaceutical, and cosmetic industries. Cultivation is concentrated in temperate regions, particularly Germany, the United States, and the Czech Republic, which rank among the world’s leading producers. In 2021, the cultivated area reached approximately 66,000 hectares, with a production exceeding 100,000 tons of cones [1,2].

In Brazil, production reached 88 tons in 2023 to meet demand from craft breweries, which has grown in recent years. Consequently, hop cultivation has attracted greater economic interest, primarily because it offers an alternative to reduce reliance on imports, add value to domestic agricultural output, and strengthen regional supply chains linked to the brewing sector [3,4,5].

Studies conducted in various regions indicate that hop development is favored by temperatures between 20 and 30 °C, conditions frequently observed during spring and summer [4]. However, beyond temperature, water availability also plays a decisive role in crop performance, as inadequate rainfall patterns can reduce yield and alter the chemical composition of the cones [6]. In general, environmental factors such as temperature, precipitation, relative humidity, and solar radiation influence hop secondary metabolism, affecting the synthesis of alpha and beta acids and essential oils. Consequently, variations in cultivation conditions can alter both the quantity and composition of these metabolites, compromising the quality and industrial application of the hops [7,8].

*Humulus lupulus* leaves play a fundamental role in the plant’s growth and development, serving as the primary photosynthetic organs that produce photoassimilates and metabolic precursors required for secondary metabolite biosynthesis [9]. Beyond their physiological importance, they represent a significant fraction of the crop’s biomass, yet their commercial utilization remains limited.

Nevertheless, growing interest in utilizing *H. lupulus* leaves has revealed their potential as a source of bioactive compounds with antioxidant, antimicrobial, and anti-inflammatory activities, supporting their potential applications in the pharmaceutical, cosmetic, food, and bioproduct industries. Thus, using leaf biomass can add value to crop co-products and reduce the waste generated after cone harvesting [10,11,12].

The northwestern region of Paraná state, Brazil, features a subtropical climate characterized by high temperatures for much of the year and a seasonal rainfall pattern Peel et al. [13], conditions quite distinct from those observed in traditional hop-growing regions. Although these characteristics make the region promising for the crop’s adaptation, there is still a scarcity of studies simultaneously evaluating the agronomic performance, essential oil production, and chemical composition of cones and leaves across different varieties over consecutive growing cycles, considering both the pre- and post-dormancy periods. Such information is crucial for understanding how varieties adapt to subtropical climatic conditions and for identifying materials with the greatest productive and technological potential [14].

In this context, evaluating varieties with agronomic and technological potential helps identify those best adapted to Brazilian soil and climatic conditions. The Columbus, Cascade, Nugget, and Comet varieties were selected for their commercial relevance and contrasting characteristics in yield potential, environmental adaptation, and aromatic profile, providing suitable materials for assessing agronomic performance and chemical characteristics under subtropical growing conditions [2,15,16,17,18,19].

This study evaluated the agronomic performance, cone and leaf productivity, essential oil yield, and volatile composition of the Columbus, Cascade, Comet, and Nugget varieties grown under subtropical conditions in northwestern Paraná, Brazil. Four harvests, conducted before and after the dormancy period, were evaluated considering the temperature conditions recorded throughout the experimental period.

## 2. Results and Discussion

### 2.1. Phenology and Edaphoclimatic Conditions

**Figure 1:**
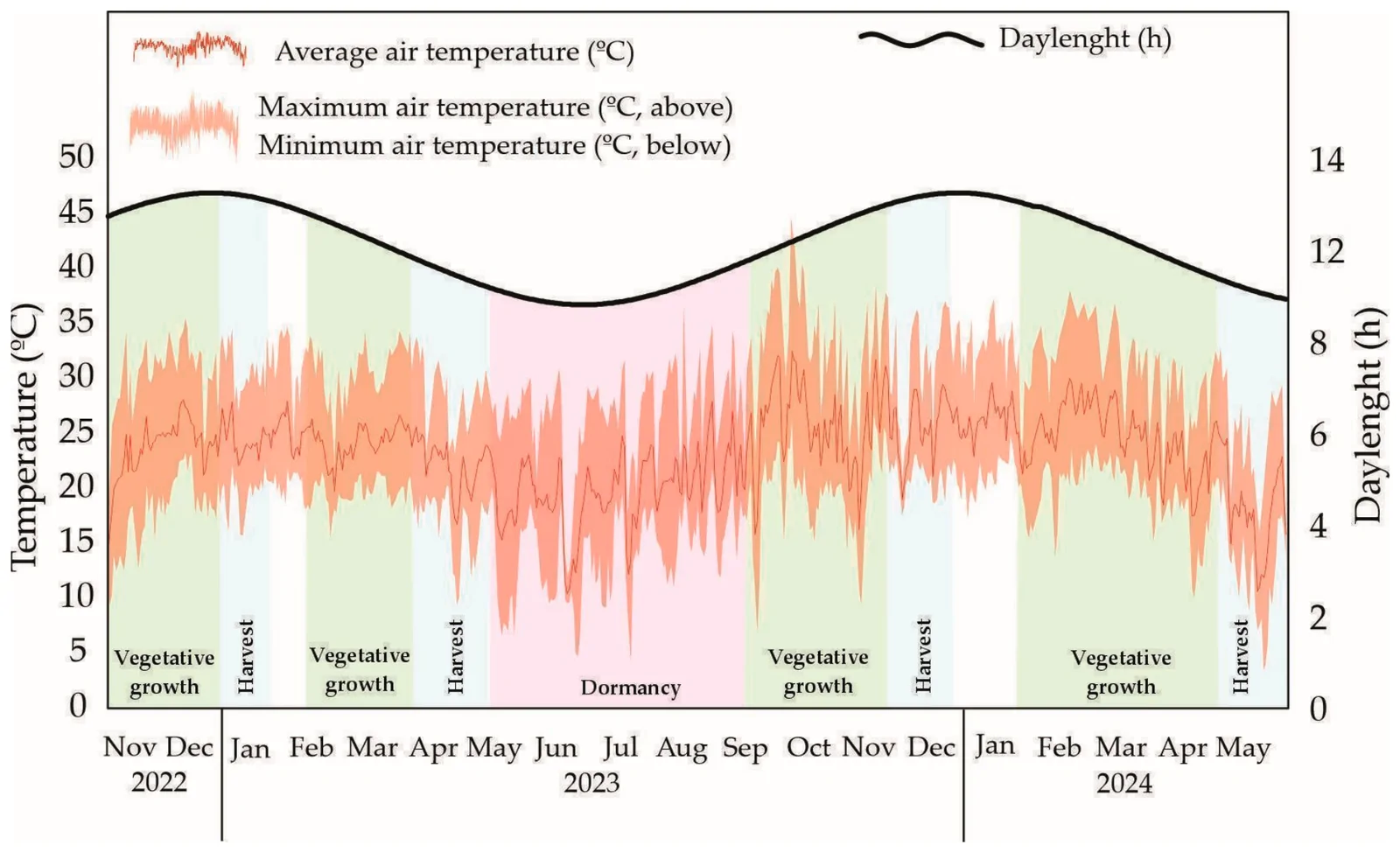
Mean, maximum, and minimum temperatures and day length during the experimental period from November 2022 to May 2024. Shaded areas indicate the phenological stages and harvest periods of *Humulus lupulus*.

**Figure 2:**
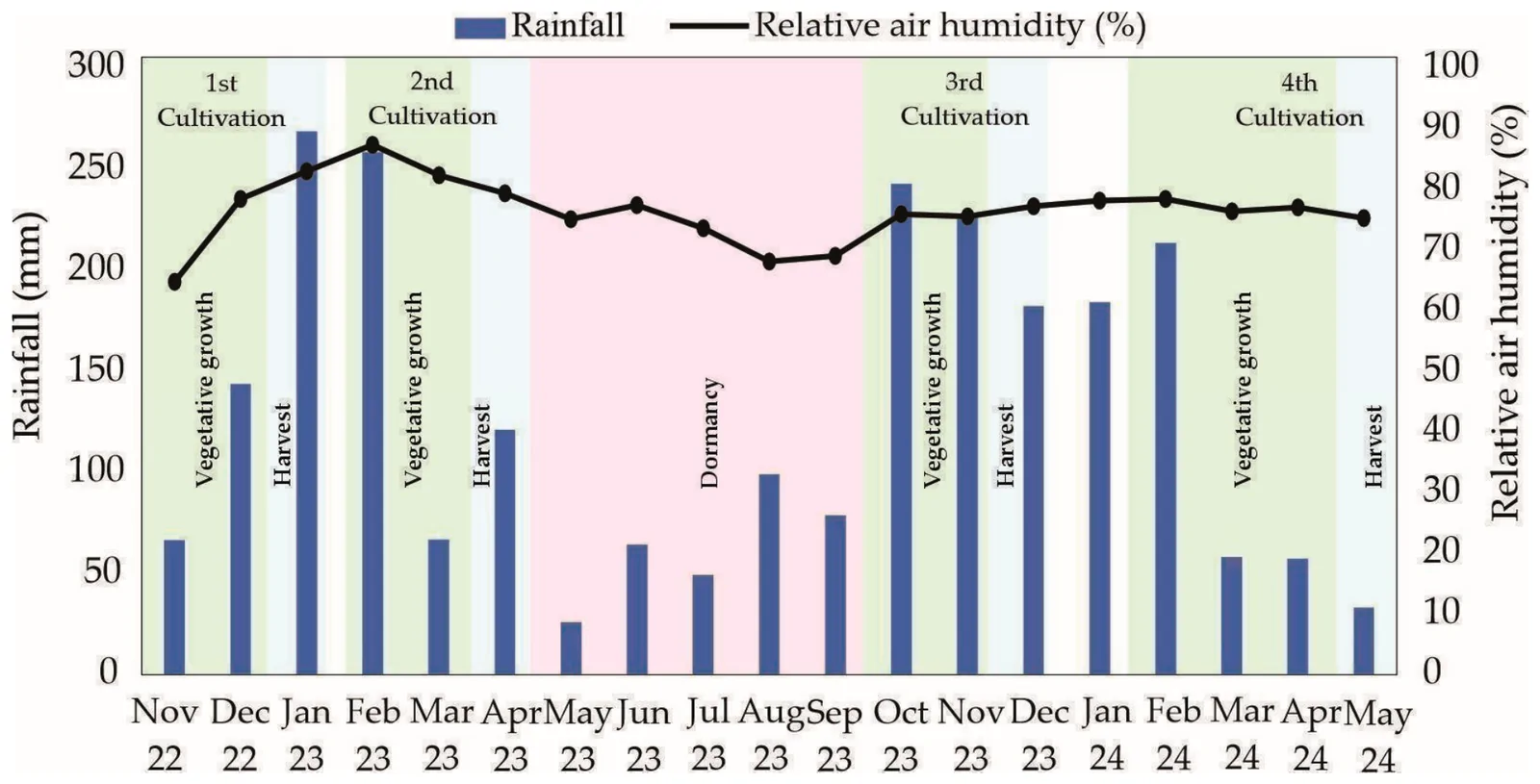
Monthly values of accumulated rainfall (mm) and relative air humidity (%) recorded in the municipality of Umuarama-PR, from November 2022 to May 2024.

Temperature data were analyzed at three times of day (Table S1): 9:00, 15:00, and 21:00. At 9:00, December 2023 had the highest mean temperature (26.77 °C). Temperatures were generally higher at 15:00, ranging from 23.01 °C in June 2023 to 31.15 °C in December 2023, whereas at 21:00 they ranged from 18.61 °C to 25.33 °C during the same months, respectively. Overall, December 2023 showed the highest mean temperatures at all three evaluated times. These temperature variations occurred alongside changes in photoperiod, precipitation, and relative humidity throughout the two growing seasons (2022/2023 and 2023/2024), under which the four hop varieties completed their respective phenological cycles (Figure 1 and Figure 2, Table S2). Such environmental variation is particularly relevant for hop cultivation because temperature, photoperiod, and water availability can influence vegetative growth, flowering, cone development, and ultimately secondary metabolite accumulation.

The experiment ran from 2 November 2022, to 20 May 2024, covering two growing seasons and four harvests of cones and leaves. In the first growing season, harvests were performed in January 2023 (summer) and April 2023 (autumn), followed by a winter dormancy period from June to September. After dormancy, the plants resumed vegetative growth, resulting in two additional harvests in December 2023 and May 2024. Following each harvest, the stems were completely pruned, allowing the plants to initiate a new vegetative cycle.

Under temperate conditions, hop production typically involves winter dormancy, spring vegetative growth, and a single harvest in late summer or early autumn. This phenological pattern is strongly influenced by photoperiod, with hop plants generally requiring longer day lengths during the vegetative phase, whereas shorter photoperiods promote the transition toward reproductive development. Gutiérrez et al. [20] reported that plants exposed to 13.5 and 16.5 h of light flowered normally, whereas longer photoperiods of 20 and 24 h favored vegetative growth and inhibited flowering; conversely, an 8-h photoperiod induced dormancy. Thus, photoperiodic responses are closely associated with the timing of flowering and dormancy in *H. lupulus*.

In the present study, the four varieties were grown under substantially shorter natural photoperiods than those commonly experienced in major temperate hop-producing regions, ranging from approximately 10.75 h at the winter solstice to 13.5 h at the summer solstice (Figure 1). Despite the absence of supplemental lighting, the plants completed two productive cycles within each growing season following severe post-harvest pruning. The occurrence of two annual harvests may therefore reflect the interaction between the subtropical photoperiodic regime and the adopted cultivation practices, particularly post-harvest pruning, which promoted the initiation of a new vegetative cycle [21]. Similar approaches have been investigated under subtropical conditions in Florida (28° N) and Paraná (24° S), although supplemental artificial lighting was used to extend the photoperiod [22,23].

The phenological pattern observed in the present study differs from that generally reported for temperate production regions, where a single annual harvest is commonly associated with the seasonal cycle of dormancy and vegetative growth. This pattern has been reported in Lanuvio, Italy [24], Prosser, Washington, USA [25], and Palermo, Italy [26,27]. However, the occurrence of two harvests in the present study cannot be attributed exclusively to photoperiod, because temperature, water availability, genotype, and pruning management also varied throughout the experimental period. Nevertheless, the results indicate that subtropical environmental conditions, combined with appropriate crop management, may provide a favorable window for multiple productive cycles without artificial photoperiod extension.

Differences in phenological development between temperate and subtropical environments may also affect secondary metabolite accumulation. The biosynthesis of volatile compounds in *H. lupulus* is influenced by the interaction between genotype and environmental conditions, including temperature, photoperiod, and water availability. Consequently, the distinct environmental conditions experienced during the four harvest periods may have contributed to the differences observed among varieties and harvests in the present study, as previously reported by Aqeel et al. [8] and Marceddu et al. [27].

Rainfall and relative humidity were monitored throughout the experimental period, and the results are presented in Figure 2. Total accumulated rainfall was 2090 mm from November 2022 to May 2024, with marked monthly variation.

Rainfall was generally higher during spring and summer and lower during autumn and winter. Rainfall was particularly high in January and February 2023, while lower values were recorded in the subsequent months of the second production cycle. Relative humidity remained comparatively stable throughout the experimental period, although moderate monthly fluctuations were observed. During the winter dormancy period, lower rainfall and the predominantly sandy texture and high permeability of the Caiuá Sandstone-derived soil required drip irrigation to maintain adequate soil moisture in the experimental plots.

During the first growing season (2022/2023), the transplanted plants established rapidly after planting in November 2022 and completed their first productive cycle by January 2023, with no marked phenological differences among varieties. This period featured a relatively narrow photoperiodic range (approximately 13–13.5 h), mean temperatures of 20–27 °C, relative humidity of approximately 85%, and relatively high rainfall. Minimum temperatures fell below 15 °C during the early vegetative phase, while maximum temperatures reached approximately 35 °C in December 2022 (Figure 1 and Figure 2). Under these conditions, all four varieties completed their first reproductive cycle and produced mature cones suitable for the first harvest. Immediately after harvest, the plants were severely pruned, initiating a new vegetative cycle.

The second production cycle of the 2022/2023 growing season ran from January to April 2023 and produced the second harvest. Following pruning, the plants resumed vegetative growth under a progressively shorter photoperiod, decreasing from approximately 13 to 11 h. Mean temperatures remained between 18 and 27 °C, although maximum temperatures reached approximately 35 °C during February and March, and minimum temperatures occasionally fell below 15 °C toward the end of the cycle. Relative humidity remained close to 80%. Rainfall was particularly high at the beginning of this cycle, exceeding 250 mm in February, but decreased substantially to approximately 60–120 mm in March and April (Figure 2). Despite this reduction in rainfall during the reproductive phase, drip irrigation maintained soil moisture; therefore, the decrease in precipitation should not be interpreted directly as evidence of water stress.

Between the two growing seasons, the plants underwent approximately four months of winter dormancy. This period was characterized by lower rainfall, with monthly values ranging from approximately 25 to 100 mm, relatively mild but variable temperatures (10–25 °C), occasional minimum temperatures near 5 °C and maximum temperatures approaching 30 °C, and a shorter photoperiod ranging from approximately 10.75 to 12 h (Figure 1 and Figure 2). These conditions differed substantially from those typically experienced by hops cultivated in temperate regions, particularly in winter temperature. Because of low rainfall and the experimental soil’s high permeability, drip irrigation was maintained to provide adequate soil moisture during dormancy.

At the beginning of the second growing season, in September 2023, the plants were pruned to remove shoots that had developed during dormancy and promote renewed vegetative growth. The spring 2023 period was characterized by relatively regular rainfall (approximately 180–240 mm per month), high relative humidity (approximately 75%), and a photoperiod increasing from approximately 12 h around the spring equinox to 13.5 h near the summer solstice (Figure 1). Compared with the corresponding period of the previous year, spring 2023 was approximately 2–3 °C warmer. It showed greater temperature variability, with mean temperatures ranging from 15 to 31 °C, occasional minimum temperatures below 10 °C, and maximum temperatures approaching 40 °C. By the end of December 2023, all four varieties had completed their phenological development, resulting in the third harvest of cones and leaves.

The fourth production cycle, from January to May 2024, was longer than the corresponding cycle in 2023, which extended from January to April. During the initial vegetative phase, rainfall ranged from approximately 180 to 210 mm, with relatively high temperatures. During the subsequent reproductive phase, however, rainfall decreased substantially to approximately 55–30 mm, while the photoperiod gradually decreased from approximately 12.5 to 11 h. Temperature also varied considerably, with maximum temperatures above 30 °C and occasional minimum temperatures below 10 °C (Figure 1 and Figure 2). Despite reduced rainfall during the later stages of this cycle, irrigation helped maintain soil moisture, allowing the plants to complete their reproductive development and reach the fourth harvest in May 2024.

Overall, the climatic conditions observed across the four production cycles indicate that hop development in the experimental region was associated with substantial seasonal and interannual variation in temperature, photoperiod, rainfall, and relative humidity. Importantly, the occurrence of two productive cycles per growing season was observed without supplemental lighting, suggesting that the subtropical environment and the adopted pruning regime provided conditions favorable for renewed vegetative growth and subsequent reproductive development. However, because photoperiod, temperature, water availability, and pruning were not experimentally isolated, the contribution of each factor to completing the second productive cycle cannot be determined independently.

These findings support the view that hop performance under subtropical conditions results from the interaction among environmental conditions, genotype, and crop management. Previous studies have emphasized the importance of temperature, water availability, and management practices in mitigating abiotic stress and maintaining hop productivity under Mediterranean and subtropical conditions [26,27]. Similarly, Marceddu et al. [27] reported that supplemental lighting can extend vegetative growth and modify the duration of hop phenological phases under Brazilian subtropical conditions. In the present study, however, no supplemental lighting was used, indicating that the observed phenological responses occurred under the natural photoperiodic regime of the experimental region. These results are comparable to those reported by Leles et al. [23] and Marceddu et al. [27], while also highlighting the potential role of post-harvest pruning in enabling multiple productive cycles under subtropical conditions.

### 2.2. Biomass and Essential Oil Production

**Figure 3:**
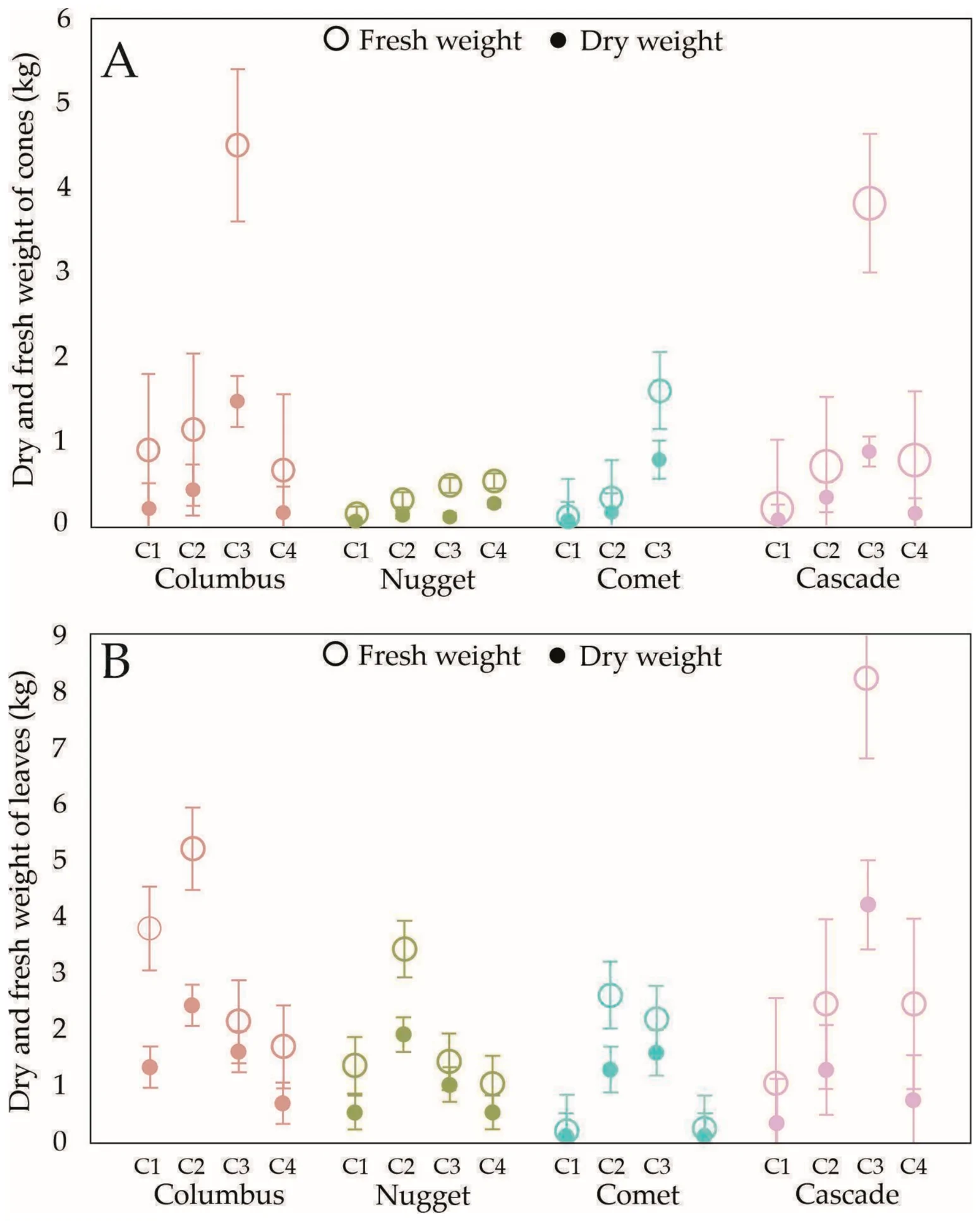
Yield (kg) of fresh weight (FW) and dry weight (DW) of cones (**A**) and leaves (**B**) of the *Humulus lupulus* varieties in four cultivations: 1st Cultivation (November 2022–January 2023); 2nd Cultivation (February–April 2023); 3rd Cultivation (September–December 2023) and 4th Cultivation (January–May 2024).

Fresh and dry biomass production of cones and leaves varied descriptively among the evaluated varieties and cultivation cycles (Figure 3 and Table S3). Data were analyzed using descriptive statistics and are presented as means with their respective standard deviations (vertical bars). The means were calculated from three field replicates for each variety in each of the four cultivation cycles: C1, November 2022 to January 2023; C2, February to April 2023; C3, September to December 2023; and C4, January to May 2024.

Overall, the third harvest, conducted in December 2023, showed the greatest biomass accumulation and cone production, coinciding with higher temperatures after the resumption of vegetative growth (Figure 3A and Table S3). During this harvest, Columbus exhibited the highest cone production, whereas Cascade accumulated the greatest leaf biomass, indicating differences in biomass allocation among varieties under the cultivation conditions evaluated. Fortuna et al. [28], who evaluated five *H. lupulus* varieties in Botucatu, Brazil, also reported high cone productivity for Columbus, Chinook, and Cascade, supporting the favorable agronomic performance of these varieties under Brazilian growing conditions.

Cascade exhibited the greatest leaf biomass accumulation throughout the experiment, particularly during the third harvest in December 2023, when temperatures ranged from 16.32 °C to 31.77 °C (Figure 3B). This result is consistent with findings by Eriksen et al. [29], who reported relatively high carbon assimilation rates and physiological stability in Cascade under temperatures ranging from 24 °C to 39 °C. Cascade’s physiological performance under elevated temperatures may therefore contribute to its ability to maintain vigorous vegetative growth under the thermal conditions observed in the present study. The greater leaf biomass production observed in this variety suggests a stronger investment in vegetative growth. In contrast, Columbus’s higher cone productivity may reflect greater allocation of photoassimilates to reproductive structures. These differences may reflect genotypic variation in source–sink relationships and distinct patterns of carbon partitioning between vegetative growth and reproduction [30].

The observed differences in biomass allocation may consequently reflect variety-specific responses to the environmental conditions prevailing during cultivation. These responses may also relate to secondary metabolite biosynthesis, since environmental factors can influence photoassimilate availability and the metabolic pathways involved in producing volatile compounds [8,30]. Therefore, differences in vegetative and reproductive biomass accumulation may affect not only agronomic productivity but also the chemical composition and potential utilization of different plant tissues.

Beyond agricultural productivity, the substantial accumulation of leaf biomass has implications for industrial utilization of the crop. The identification of α-humulene and β-caryophyllene in essential oils extracted from the leaves of all four varieties demonstrates the presence of volatile metabolites characteristic of *Humulus lupulus* in these tissues. Although essential oil yield was lower in leaves than in cones, the substantial availability of leaf biomass, together with its volatile composition, indicates that these underutilized tissues may have valorization potential [31]. Thus, using leaves may provide an opportunity to add value to residual biomass from hop cultivation and support more comprehensive and sustainable crop use. Further studies addressing extraction efficiency, scalability, and economic feasibility are needed to assess the practical potential of this approach.

### 2.3. Essential Oil Yields (%)

**Figure 4:**
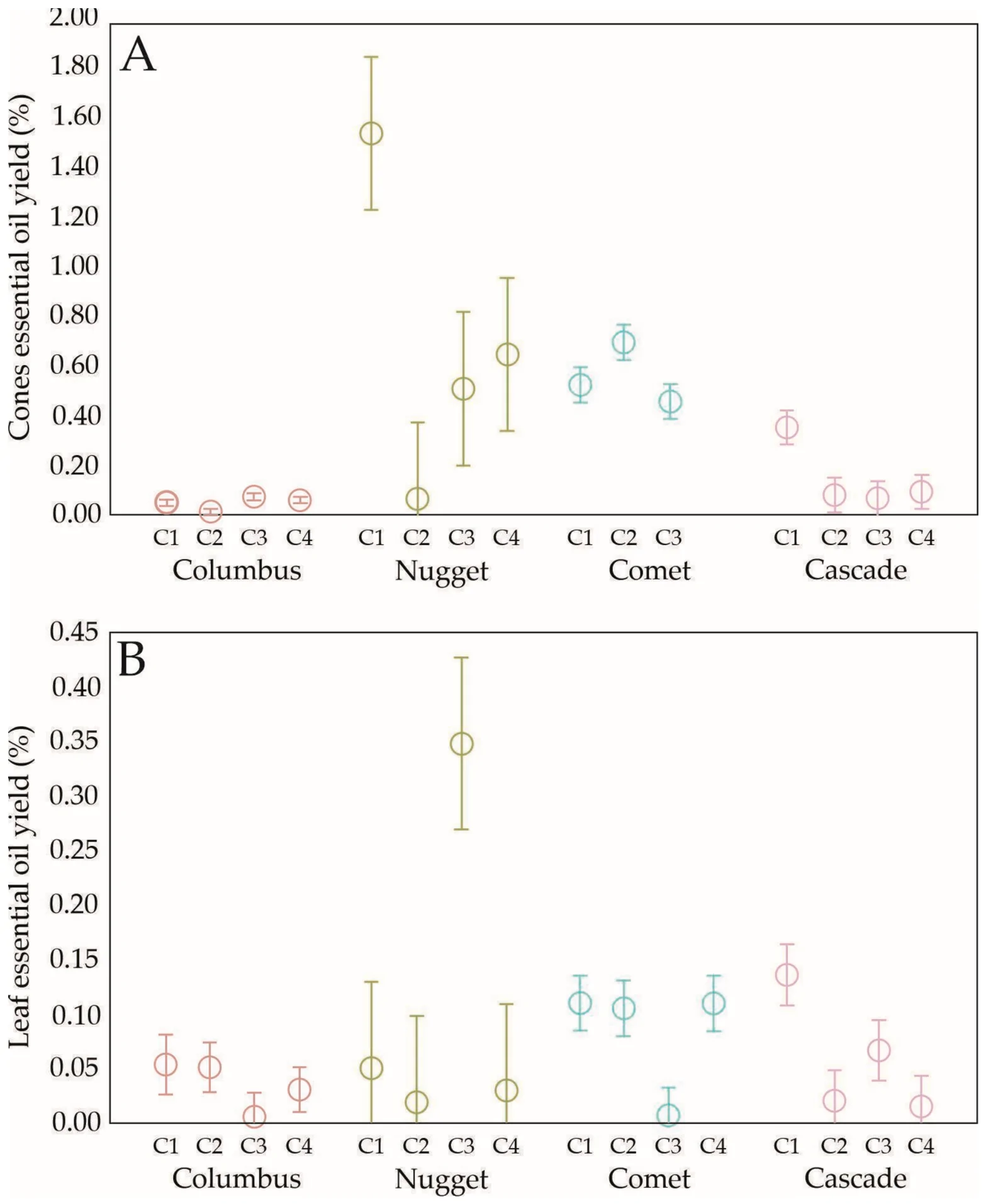
Yield (%) of essential oil of the cones (**A**) and leaves (**B**) of the Cascade, Columbus, Comet, and Nugget varieties of *Humulus lupulus*, in four cultivation periods: 1st Cultivation (November 2022–January 2023); 2nd Cultivation (February–April 2023); 3rd Cultivation (September–December 2023) and 4th Cultivation (January–May 2024).

Table S4 and Figure 4 present essential oil yields (%) from the leaves and cones of the four hop varieties across the two growing seasons. Comparison of these results with biomass production showed that higher leaf or cone biomass did not consistently correspond to higher essential oil yield.

Essential oil yields showed numerical variation among varieties, plant parts, and harvests. The highest numerical yield was observed for Nugget cones in the first harvest (1.532 ± 0.399%), whereas the lowest numerical values were observed in several leaf samples. Because each variety × plant part × harvest combination was represented by a single composite sample, these differences are presented and interpreted descriptively rather than as statistically tested differences.

Across the samples evaluated, cones generally showed higher numerical essential oil yields than leaves (Figure 4 and Table S4). This result is consistent with the high abundance of lupulin glands in hop cones, specialized secretory structures involved in the biosynthesis and accumulation of terpenes and other aromatic compounds. The lower density of secretory structures in leaves may account for their lower essential oil yields, although this tissue also contains volatile compounds characteristic of the species [32].

The low essential oil yield observed in leaves is consistent with the results reported by Katsiotis et al. [33], who reported essential oil yields of less than 0.05% in the leaves of various *H. lupulus* varieties. Despite the low yield, α-humulene and β-caryophyllene were among the predominant compounds tentatively identified in the leaf essential oils, consistent with the presence of volatile compounds characteristic of *H. lupulus* in this tissue. Furthermore, although essential oil production in the leaves is considerably lower than in the cones, the high leaf biomass production observed under the subtropical conditions of the present study may partially offset this limitation, supporting the potential use of this agricultural residue as a complementary source of bioactive compounds [29,31].

Among the evaluated varieties, Nugget showed the highest numerical essential oil yields, ranging from 0.069% to 1.53% across the four cultivation cycles, followed by Comet, with values ranging from 0.459% to 0.696% (Figure 4 and Table S4). These numerical differences may reflect variation in the capacity to synthesize and accumulate volatile compounds, although this interpretation cannot be statistically tested with the present experimental design. Such variation may be associated with genetic characteristics, including lupulin gland density and terpene biosynthetic pathways, as well as environmental conditions during cultivation.

The essential oil yields obtained in the present study also differed from those previously reported for hops grown in Brazil. The highest yield recorded for Nugget (1.53%) exceeded the approximately 1.07% reported by Sabino et al. [7]. In contrast, the yields obtained for Comet (0.53–0.69%) were lower than the 1.34–1.90% range reported by Marceddu et al. [27] and the 2.36% reported by Sabino et al. [7]. These differences may reflect variation in genetic background, cone developmental stage, and cultivation conditions, factors known to influence the biosynthesis and accumulation of volatile compounds in hops [32].

The varieties also displayed distinct growth and production patterns, highlighting differences in resource allocation among vegetative development, cone formation, and secondary metabolite synthesis. Nugget showed the highest numerical essential oil yields, while Cascade exhibited greater vegetative growth and Columbus showed higher cone production. These results suggest that, under the subtropical conditions evaluated, differences in growth and secondary metabolism may reflect the interaction between each variety’s genetic potential and environmental factors, which are known to modulate secondary metabolite biosynthesis through physiological and metabolic changes [8,30]. Thus, the observed differences among varieties may influence both crop productivity and essential oil production and composition.

### 2.4. Essential Oil Composition

The chemical composition of essential oils from the cones of *H. lupulus* varieties Cascade, Comet, Columbus, and Nugget was analyzed by GC-MS, with the tentatively identified compounds accounting for more than 97% of the total chromatographic peak area. Compounds were putatively identified at confidence level 2 according to the Metabolomics Standards Initiative, based on comparisons of mass spectra and retention indices with reference data from spectral libraries and the literature [34]. Unless otherwise indicated, compound identities were not confirmed using authentic reference standards. Across all samples, 41 compounds were tentatively identified in the cone essential oils (Table 1) and 24 compounds were tentatively identified in the leaf essential oils (Table 2), with variations in their relative proportions among the varieties and cultivation cycles evaluated. The tentatively identified compounds were classified into six groups: monoterpene hydrocarbons, oxygenated monoterpenes, sesquiterpene hydrocarbons, oxygenated sesquiterpenes, diterpenes, and other compounds (Table 1 and Table 2).

Hydrocarbons (monoterpenes and sesquiterpenes) account for approximately 75% of total hop essential oil [77]. In the present study, β-myrcene was among the major compounds tentatively identified (characterized by herbaceous, fresh green notes reminiscent of fresh hops) and is typically the major compound, found at concentrations up to 60% of the essential oil. In addition to this monoterpene, notable sesquiterpenes include α-humulene (woody notes), β-caryophyllene (woody and spicy notes), and β-farnesene (woody, citrus, and sweet notes). These four terpenoids can make up 80% to 90% of the hydrocarbon content in many hop essential oils [77,78]. Because of their strong influence on beer characteristics, the myrcene-to-humulene ratio is commonly used to categorize hop batches (noble hops generally exhibit a humulene/myrcene ratio greater than 3.5) [77].

In the present study, hydrocarbon sesquiterpenes were the predominant chemical class observed among the tentatively identified compounds. In cones, Columbus showed the highest content of this major class, followed by Cascade, Nugget, and Comet varieties (Table 1; Figure S1). In contrast, in leaves, Comet exhibited the highest proportion, followed by Nugget, Columbus, and Cascade (Table 2 and Figure S2). Although Columbus and Cascade showed the highest levels of this chemical class and exhibited favorable growth and production performance under the environmental conditions of the northwestern Paraná region, the high abundance of this chemical class in the leaves of Nugget and Comet indicates that the overall proportion of hydrocarbon sesquiterpenes alone is insufficient to explain differences in variety performance or adaptation. Rather, variation in the relative abundance of individual tentatively identified sesquiterpenes may be more informative than the total abundance of the chemical class. Because essential oils contribute substantially to the aromatic characteristics of different hop varieties and to the aroma of beer, the predominance of sesquiterpene hydrocarbons could contribute to differences in the aromatic profiles of these varieties and, consequently, influence the sensory characteristics of beers produced with them. This interpretation is consistent with Carvalho [79], who emphasized the relevance of the specific composition of secondary metabolites to the chemical characterization of hop cultivars. Similarly, Aqeel et al. [8] reported that environmental stress can modify the biosynthesis and accumulation of specialized metabolites, contributing to differences among cultivars and harvest periods. In the present study, variation in the relative abundance of individual sesquiterpenes among cultivars, despite exposure to the same growing conditions, suggests that the chemical composition of hop essential oils reflects interactions between cultivar-specific genetic characteristics and environmental conditions [8,14].

Although chemical characterization of essential oils, through the identification and relative quantification of compounds and their respective chemical classes, provides fundamental information on the chemical profile of different *Humulus lupulus* varieties, this descriptive approach alone does not allow assessment of relationships between metabolites or identification of similarity patterns among samples. In this context, multivariate statistical analyses serve as important complementary tools for interpreting chemical data [80].

Pearson correlation analysis identified positive and negative associations between components, providing evidence of potential co-variations and relationships among the compounds present in the essential oils [81]. The complete Pearson correlation results, including the correlation coefficients, raw *p*-values, Benjamini–Hochberg-adjusted *p*-values, magnitude, and direction of the associations, are presented in Supplementary Table S5.

**Figure 5:**
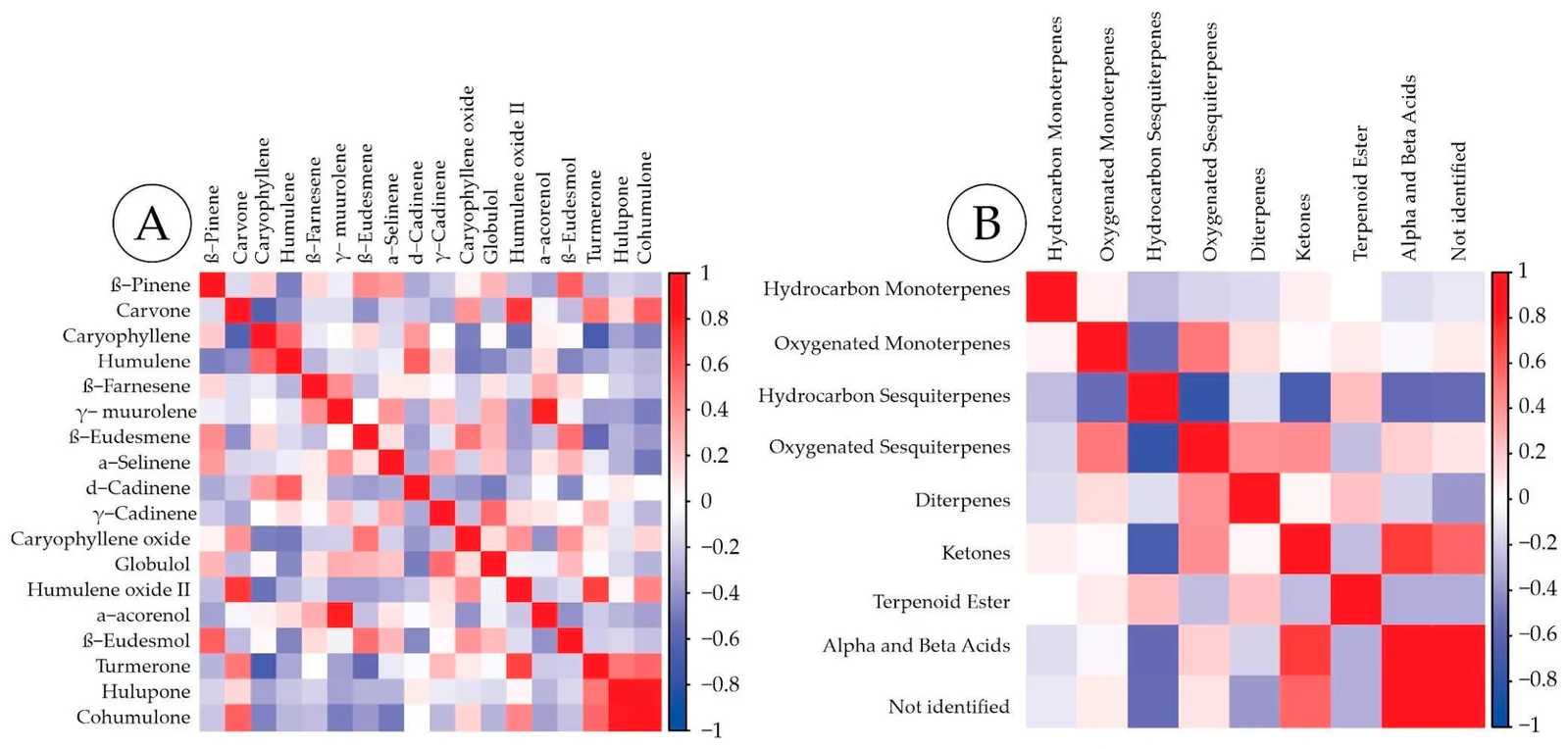
Pearson correlation matrix of terpenoids identified in essential oils extracted from cones of the Cascade, Columbus, Comet, and Nugget varieties of *Humulus lupulus* (components ≥ 6.0 in at least one sample (Table 1). (**A**) Major compounds and (**B**) compound classes. The color scale represents correlations among terpenoid relative abundances, with red indicating positive correlations and blue indicating negative correlations.

Based on the heatmap derived from the correlation matrix (Pearson correlation test) of compounds detected in the hydrodistilled essential oils of hop cone varieties, a very strong correlation was observed between bitter acids (α- and β-acids) and ketones, and between bitter acids and not-identified compounds (Figure 5B). Strong correlations were also observed between oxygenated sesquiterpenes and oxygenated monoterpenes, diterpenes, and ketones, in addition to a moderate interaction between hydrocarbon sesquiterpenes and terpenoid esters. For specific compounds (Figure 5A), a very strong correlation was found between hulupone and cohumulone, which were mainly detected in the third harvest of the Nugget variety (Table 1; Supplementary Table S5). However, the occurrence of hop bitter-acid-related compounds in hydrodistilled fractions should be interpreted cautiously, since α- and β-acids are primarily associated with the resin fraction of *H. lupulus*. In contrast, hydrodistillation may promote the co-distillation or transformation of less volatile compounds [82,83]. In particular, hulupone may result from β-acid oxidation, while α-acids may undergo thermal conversion during heating [84,85]. Therefore, the correlations involving hulupone and cohumulone should be interpreted as associations among compounds detected in the hydrodistilled fractions, rather than as evidence that these compounds necessarily represent native constituents of hop essential oil.

Among the tentatively identified compounds, other observed correlations included very strong correlations between humulene epoxide II and carvone, humulene epoxide II and turmerone, humulene and δ-cadinene, β-pinene and β-eudesmol, γ-muurolene and α-acorenol, cohumulone and carvone; strong correlations between humulene and caryophyllene, β-pinene and β-eudesmene, β-eudesmene and eudesmol, β-farnesene and γ-muurolene, globulol and γ-cadinene, turmerone and hulupone, turmerone and cohumulone. Additionally, β-eudesmene showed a moderate correlation with caryophyllene epoxide, while caryophyllene epoxide also exhibited a moderate correlation with humulene epoxide II, among others.

**Figure 6:**
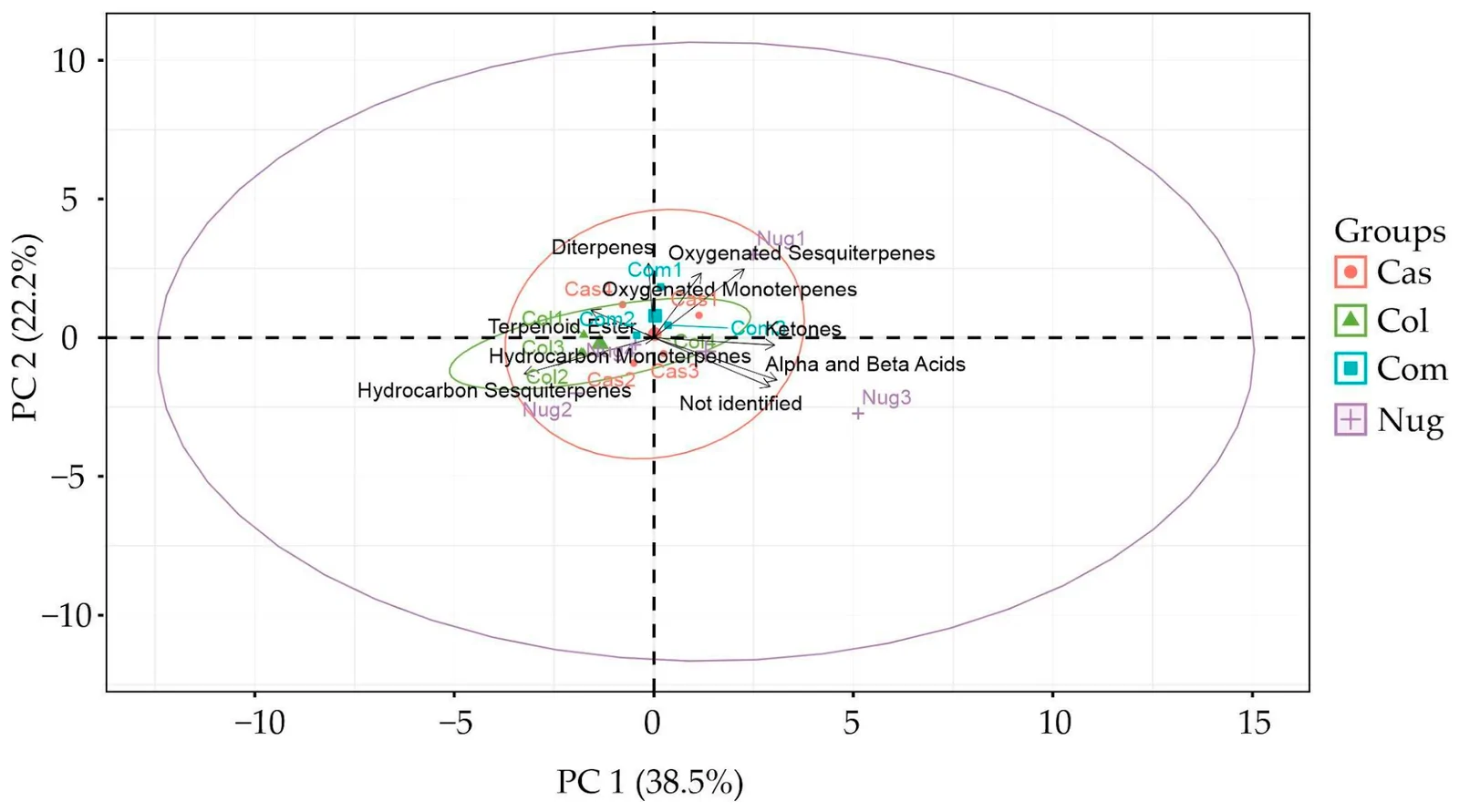
PCA biplot of the major chemical classes identified in the essential oils extracted from cones of the Cascade, Columbus, Comet, and Nugget varieties of *Humulus lupulus* across four cultivation cycles: 1st cultivation (November 2022 to January 2023), 2nd cultivation (February–April 2023), 3rd cultivation (September–December 2023), and 4th cultivation (January–May 2024). Only components with relative abundances ≥ 6.0% in at least one sample were included (Table 1). Cas: Cascade; Col: Columbus; Com: Comet; Nug: Nugget.

**Figure 7:**
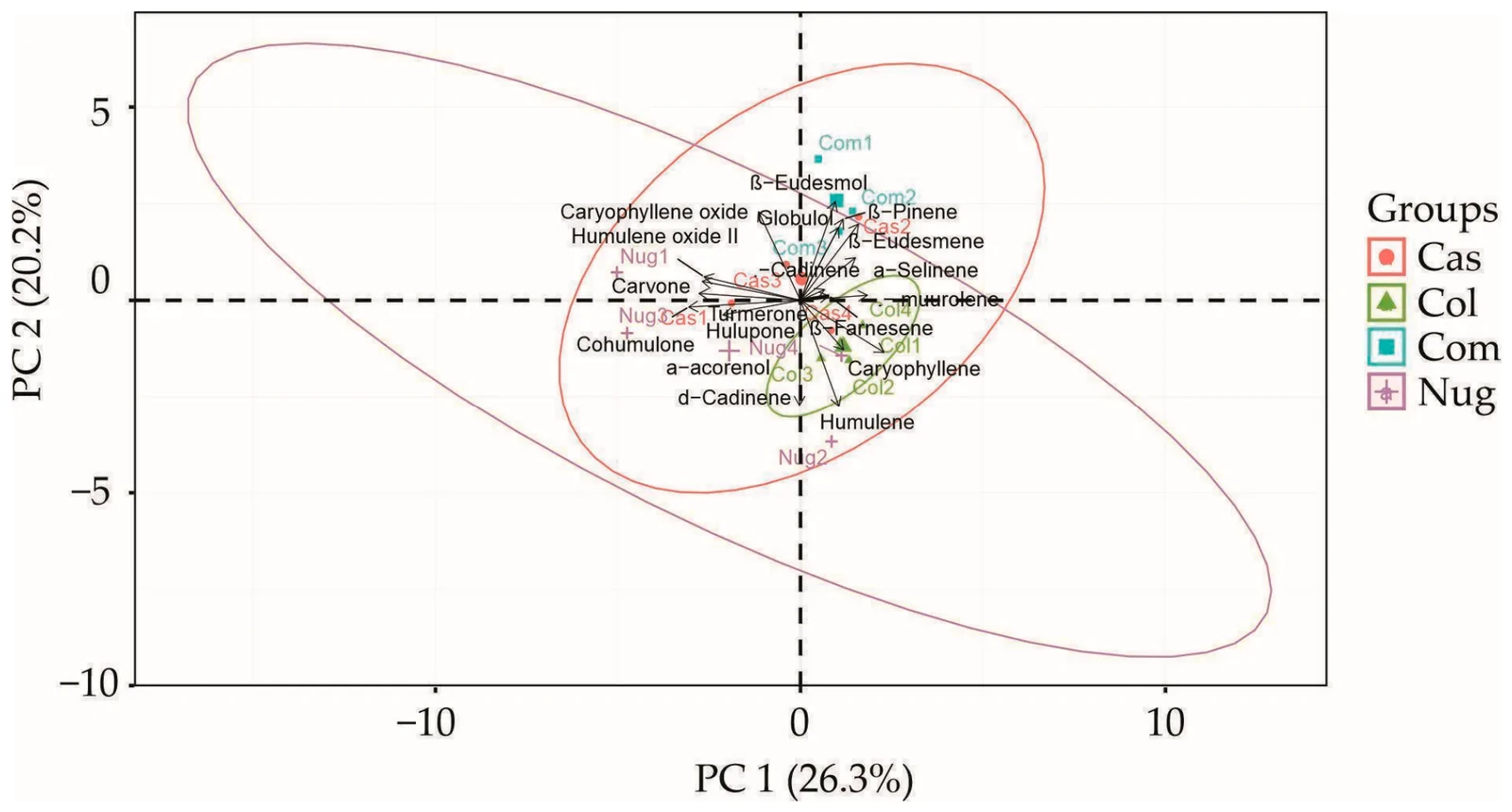
PCA biplot of the major compounds identified in the essential oil extracted from cones of the Cascade, Columbus, Comet, and Nugget varieties of *Humulus lupulus* across four cultivation cycles: 1st cultivation (November 2022 to January 2023), 2nd cultivation (February–April 2023), 3rd cultivation (September–December 2023), and 4th cultivation (January–May 2024). Only components with relative abundances ≥ 6.0% in at least one sample were included (Table 1). Cas, Cascade; Col, Columbus; Com, Comet; Nug, Nugget.

Principal Component Analysis (PCA), represented by a biplot, reduced the dimensionality of the dataset and helped visualize the contribution of the measured variables to sample separation and clustering patterns among the varieties and cultivation periods [86]. Figure 6 and Figure 7 present cone oil data for the four varieties across the four cultivation periods. The highest relative abundance of hydrocarbon sesquiterpenes was observed in Columbus across all cultivation periods, as well as in Cascade and Nugget during the second cultivation period (Figure 6). Clustering was also observed among Cascade, Comet, and Nugget during the first cultivation, suggesting similarities in their chemical profiles, particularly with respect to tentatively identified oxygenated mono- and sesquiterpenes.

PCA of the major tentatively identified compounds (Figure 7) showed associations between sample ordination and the relative abundance of specific compounds in the cone essential oils. The vectors corresponding to δ-cadinene, α-humulene, β-caryophyllene, β-farnesene, and γ-muurolene were oriented toward the region of the biplot predominantly occupied by Columbus samples. In contrast, the vectors corresponding to β-eudesmol, globulol, β-pinene, β-eudesmene, γ-cadinene, α-selinene, and caryophyllene oxide were oriented toward the opposite region of the biplot, predominantly occupied by Comet samples.

**Figure 8:**
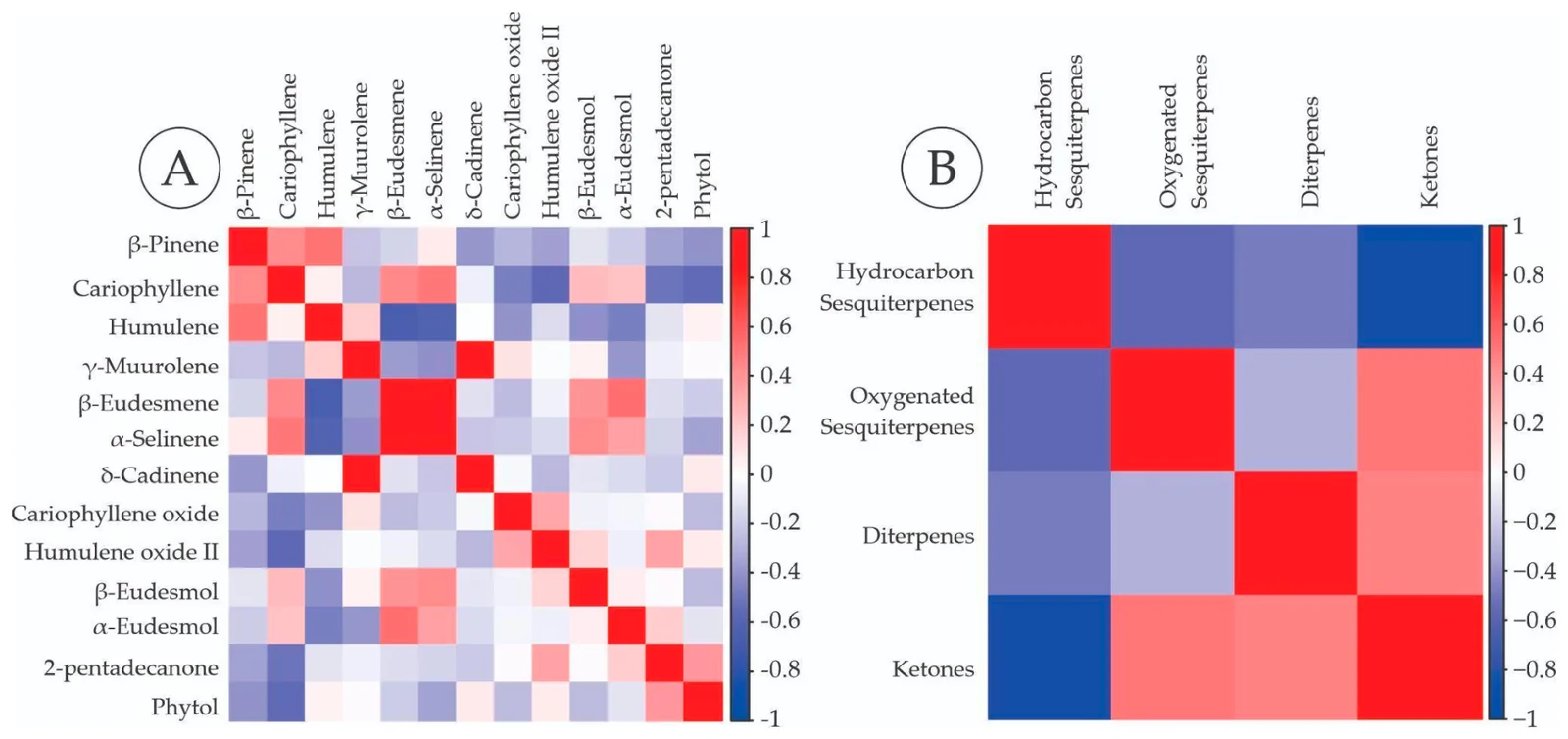
Pearson correlation matrix of compounds identified in essential oils from leaves of the Cascade, Columbus, Comet, and Nugget varieties of *Humulus lupulus* across four cultivation cycles: 1st cultivation (November 2022 to January 2023), 2nd cultivation (February–April 2023), 3rd cultivation (September–December 2023), and 4th cultivation (January–May 2024). Only components with relative abundances ≥ 6.0% in at least one sample were included (Table 2). (**A**) Major compounds and (**B**) compound classes. The color scale represents correlations among compound relative abundances, with red indicating positive correlations and blue indicating negative correlations.

Figure 8 shows the groupings of the essential oils extracted from the leaves. According to the heatmap derived from the correlation matrix of these oils, correlations were observed between oxygenated sesquiterpenes and ketones, as well as between ketones and diterpenes (Figure 8B). The complete Pearson correlation results for the leaf essential oils, including the correlation coefficients, raw *p*-values, Benjamini–Hochberg-adjusted *p*-values, magnitude, and direction of the associations, are provided in Supplementary Table S5. Among the tentatively identified individual compounds, the strongest correlations were observed between γ-muurolene and δ-cadinene, as well as between β-eudesmene and δ-cadinene (Figure 8A). Strong correlations were also found between humulene and β-pinene, α-selinene and caryophyllene, and β-eudesmene and α-eudesmol, in addition to other moderate correlations, such as between β-pinene and caryophyllene and between phytol and 2-pentadecanone.

**Figure 9:**
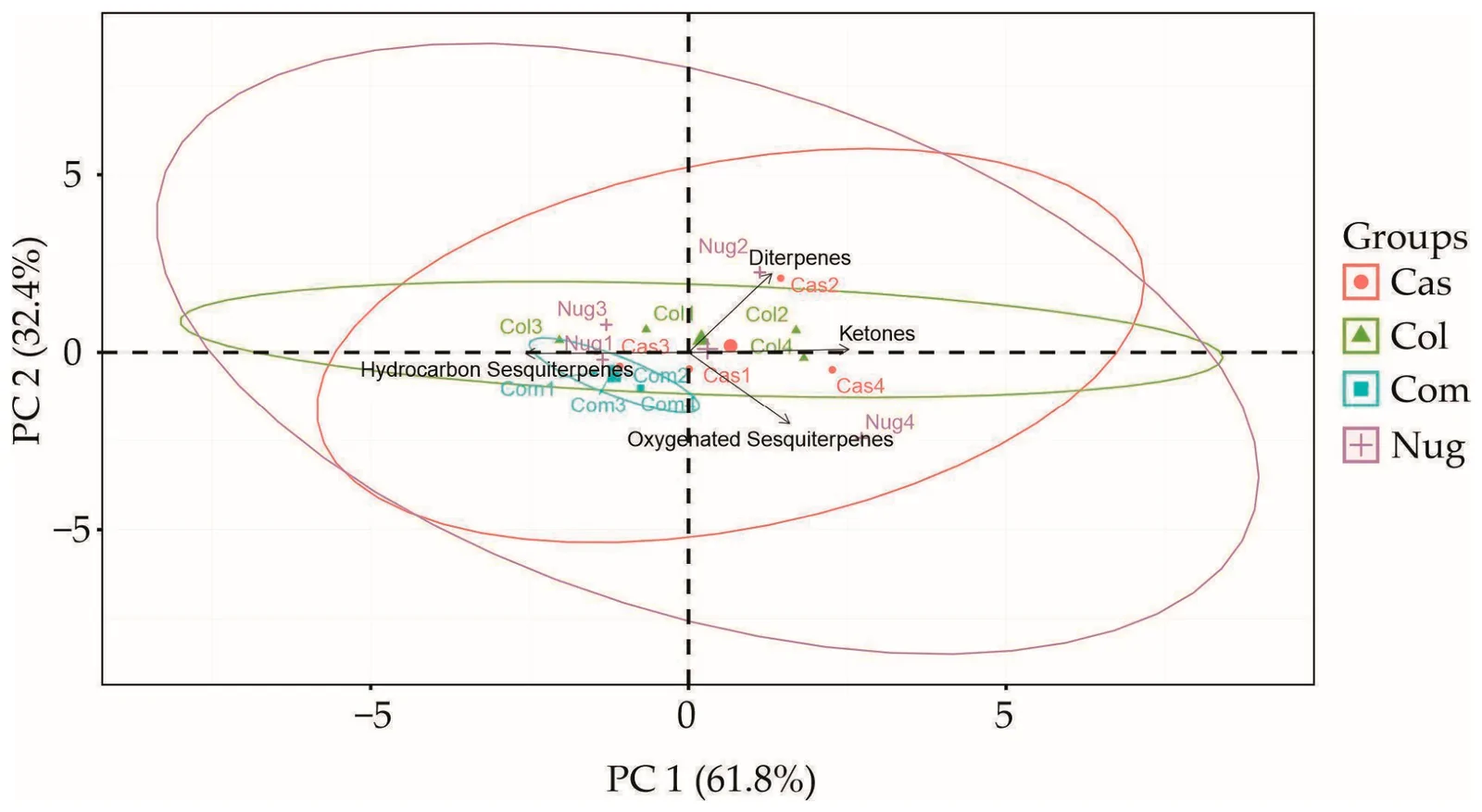
PCA biplot of the major chemical classes identified in essential oils from leaves of the Cascade, Columbus, Comet, and Nugget varieties of *Humulus lupulus* across four cultivation cycles: 1st cultivation (November 2022 to January 2023), 2nd cultivation (February–April 2023), 3rd cultivation (September–December 2023), and 4th cultivation (January–May 2024). Only components with relative abundances ≥ 6.0% in at least one sample were included (Table 2). Cas: Cascade; Col: Columbus; Com: Comet; Nug: Nugget.

**Figure 10:**
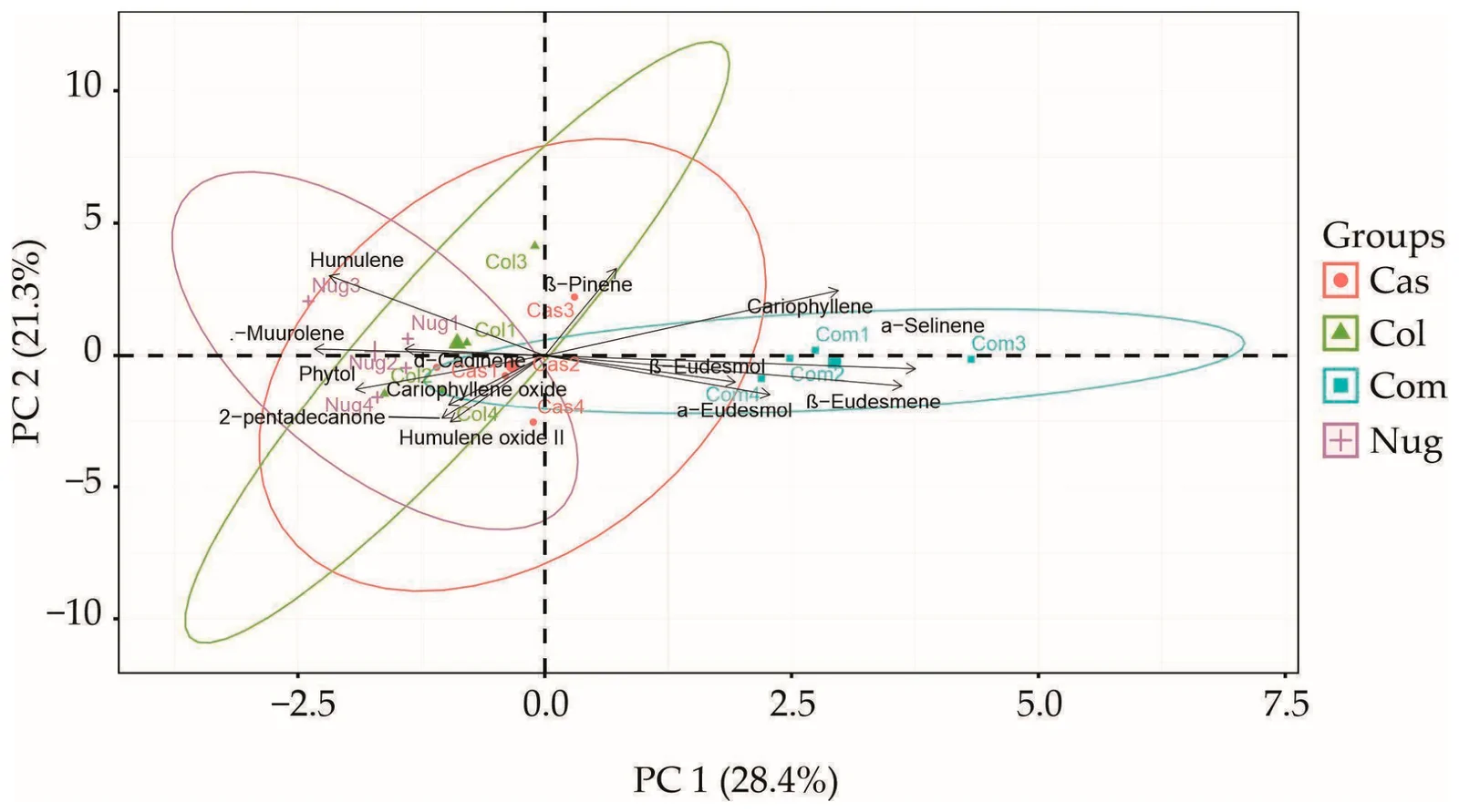
PCA biplot of the major compounds identified in essential oils from leaves of the Cascade, Columbus, Comet, and Nugget varieties of *Humulus lupulus* across four cultivation cycles: 1st cultivation (November 2022 to January 2023), 2nd cultivation (February–April 2023), 3rd cultivation (September–December 2023), and 4th cultivation (January–May 2024). Only components with relative abundances ≥ 6.0% in at least one sample were included (Table 2). Cas: Cascade; Col: Columbus; Com: Comet; Nug: Nugget.

The principal component analysis (PCA) of the four varieties in the first and third harvests (spring/summer cultivations) showed an association with hydrocarbon sesquiterpenes (Figure 9 and Table 2). In the second and fourth cultivations, Columbus, Cascade, and Nugget varieties clustered near the vectors associated with diterpenes and oxygenated sesquiterpenes, suggesting that the chemical composition of the leaves varied across harvests (Figure 9). PCA also showed a predominance of oxygenated sesquiterpenes in the Nug4 variety, which accounted for 60.59% of the total relative peak area. In addition, the occurrence of diterpenes in the Cas2 and Nug2 varieties was noteworthy, suggesting differences in the relative abundance of these compounds among the evaluated varieties (Table 2). The ordination analysis of the tentatively identified compounds across the four varieties (Figure 10) showed that, in addition to α-humulene and β-caryophyllene, phytol was also an important vector associated with sample separation. Notably, caryophyllene, α-selinene, β-eudesmene, and α-eudesmol were consistently associated with samples from the Comet variety, suggesting that these tentatively identified compounds were associated with the chemical profile observed for this variety.

These results suggest that differences among leaf samples reflected variation in the relative abundance of volatile compounds and other compounds detected in the essential oils. The chemical profiles of the leaves differed from those observed in the cones, particularly in the relative contribution of hydrocarbon and oxygenated sesquiterpenes and compounds such as phytol.

The PCA results suggest that, under the subtropical conditions evaluated, the characteristic chemical profile of *Humulus lupulus* was maintained while quantitative variation occurred in the relative abundance of chemical classes and major tentatively identified compounds. Whereas cones differed primarily in the relative abundance of aroma-related sesquiterpenes, leaves showed a stronger association with phytol and other compounds related to the chemical composition of vegetative tissues. These findings support the interpretation that essential oil chemical composition may reflect interactions between variety-specific genetic characteristics and environmental cultivation conditions, consistent with previous reports [14,87,88].

**Figure 11:**
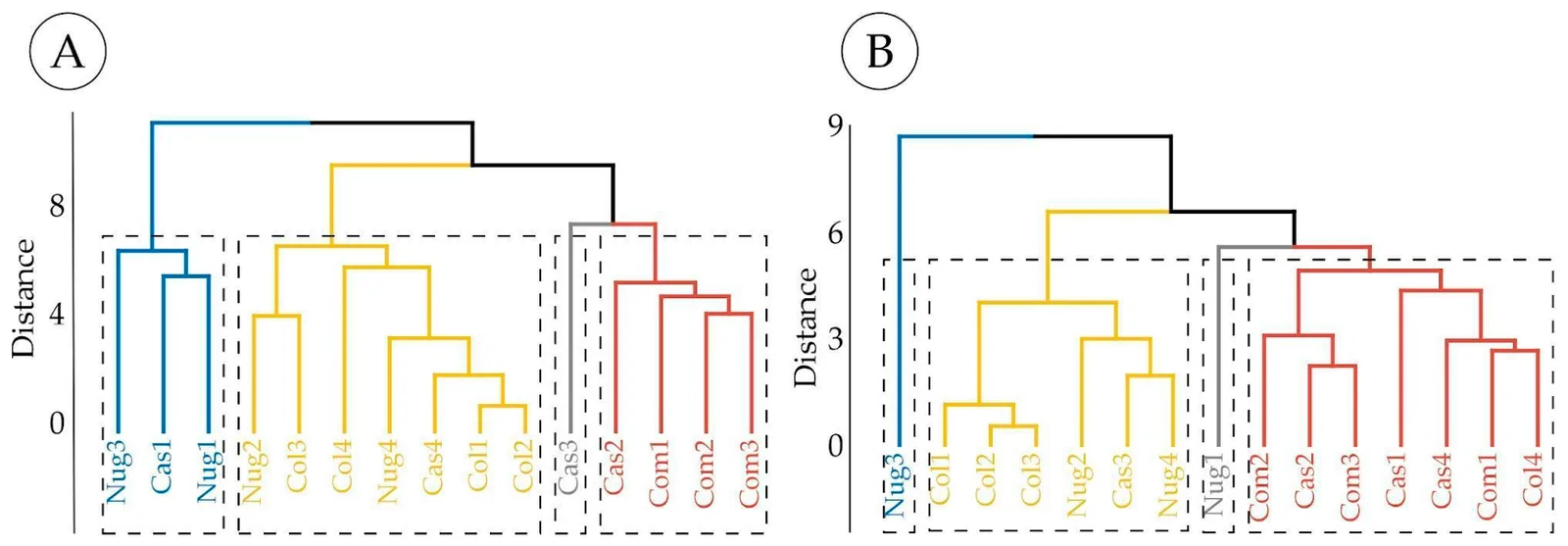
Hierarchical cluster analysis (HCA) based on the chemical composition of essential oils extracted from cones of the Cascade, Columbus, Comet, and Nugget varieties of *Humulus lupulus* across four cultivation cycles: 1st cultivation (November 2022 to January 2023), 2nd cultivation (February–April 2023), 3rd cultivation (September–December 2023), and 4th cultivation (January–May 2024). Only components with relative abundances ≥ 6.0% in at least one sample were included (Table 1). (**A**) Individual compounds and (**B**) compound classes. Cas: Cascade; Col: Columbus; Com: Comet; Nug: Nugget; numbers 1–4 indicate the respective cultivation cycles.

**Figure 12:**
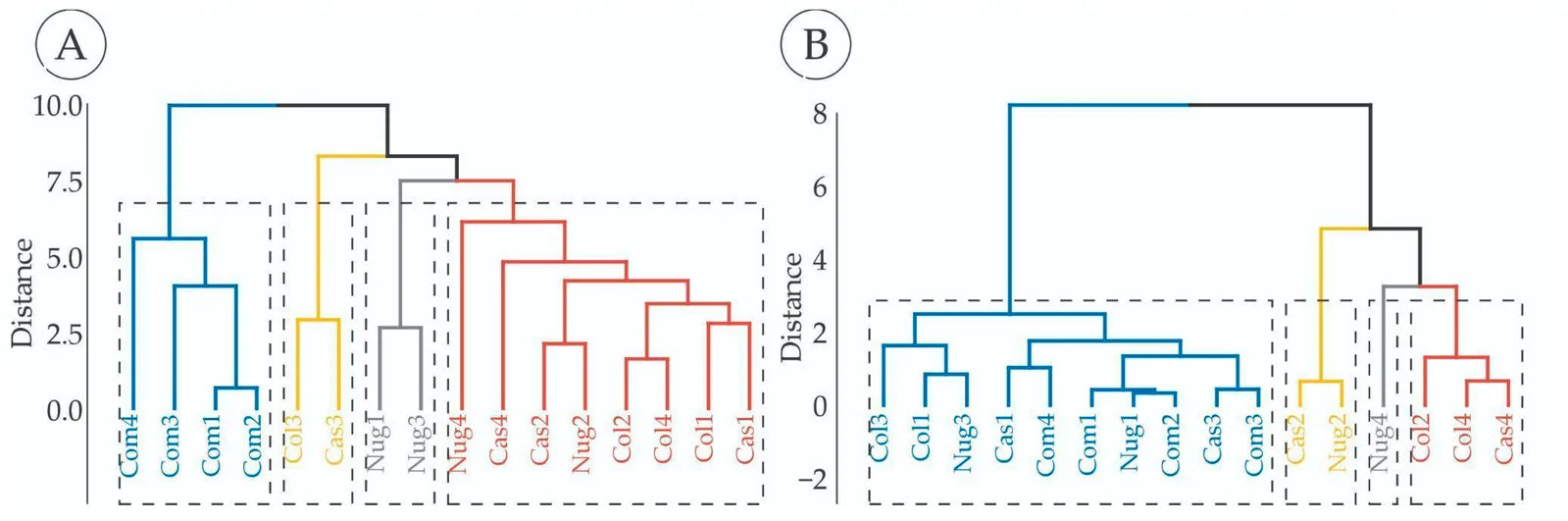
Hierarchical cluster analysis (HCA) based on the chemical composition of essential oils extracted from leaves of the Cascade, Columbus, Comet, and Nugget varieties of *Humulus lupulus* across four cultivation cycles: 1st cultivation (November 2022 to January 2023), 2nd cultivation (February–April 2023), 3rd cultivation (September–December 2023), and 4th cultivation (January–May 2024). Only components with relative abundances ≥ 6.0% in at least one sample were included (Table 2). (**A**) Individual compounds and (**B**) compound classes. Cas: Cascade; Col: Columbus; Com: Comet; Nug: Nugget; numbers 1–4 indicate the respective cultivation cycles.

In turn, a hierarchical cluster analysis (HCA) assessed the chemical similarity among the varieties, grouping those with similar chemical profiles [80]. Together, these approaches broadened the interpretation of the results, enabling an integrated assessment of the chemical variability of essential oils extracted from the cones and leaves of the *Humulus lupulus* varieties Cascade, Columbus, Comet, and Nugget (Figure 11 and Figure 12). Thus, it was possible to understand not only the individual chemical composition of each variety but also the relationships between compounds, as well as the patterns of similarity and differentiation among the chemical profiles.

Studies conducted in different hop-growing regions indicate that the essential oils of *H. lupulus* cones and leaves are characterized by the recurrent occurrence of several sesquiterpenes. However, their relative abundance may vary according to variety, plant tissue, and growing conditions. Compounds commonly reported in *H. lupulus* essential oils include α-humulene, β-caryophyllene, δ-cadinene, caryophyllene oxide, and T-cadinol [87,88]. Among these compounds, α-humulene is recognized as one of the major sesquiterpenes in hop essential oil and was among the earliest constituents identified in hop oil, while β-caryophyllene is also consistently reported as a major sesquiterpene component [17]. In the present study, α-humulene and β-caryophyllene were among the major compounds tentatively identified in the essential oils of both cones and leaves across the four varieties evaluated (Table 1 and Table 2), indicating that these tissues shared part of the chemical profile commonly reported for *H. lupulus*, although differences in the relative abundance of individual compounds were observed [29]. The recurrent detection of these sesquiterpenes across varieties and plant tissues is consistent with their characteristic occurrence in *H. lupulus*. In contrast, differences in their relative abundance may reflect the combined effects of genetic background, tissue-specific metabolism, and growing conditions.

The occurrence and predominance of these sesquiterpenes have also been reported in hop varieties grown under different environmental conditions such as those reported by Forteschi et al. [89] found that the essential oil of Cascade cones cultivated in Chilivani (Ozieri), Sardinia, Italy, contained 24.0–25.6% α-humulene and 9.4–11.2% β-caryophyllene. Similarly, Eyres and Dufour [90] identified α-humulene and β-caryophyllene as major constituents in the essential oils of Cascade, Columbus, and Nugget cultivated in the Marche region of Italy, with α-humulene ranging from 16.42 to 18.16% and β-caryophyllene from 8.03 to 8.70%. In hop leaves, Ovidi et al. [88] reported α-humulene (27.8%) and β-caryophyllene (19.2%) among the major constituents of Chinook essential oil. In addition, Rutnik et al. [32] and Katsiotis et al. [33] reported substantial variation in α-humulene abundance among hop cultivars, indicating that although this compound is recurrently detected in *H. lupulus*, its accumulation may vary considerably among genotypes.

A particularly relevant comparison is provided by Sabino et al. [7], who evaluated the Cascade, Columbus, Comet, and Nugget varieties under Brazilian growing conditions in Botucatu, São Paulo (22°55′ S). Their analysis of hop essential oils indicated that α-humulene represented 0.51% of the oil in Comet and reached 11.34% in Cascade, whereas (E)-caryophyllene ranged from 3.42% in Nugget to 9.70% in Comet. These results indicate that, despite the recurrent occurrence of these sesquiterpenes across varieties, their relative abundance may differ markedly according to genetic background.

This variation is also found in our research, in which Columbus showed the highest α-humulene abundance in both cones and leaves (Table 1 and Table 2). This cultivar-specific accumulation may contribute to differences in the chemical composition and aromatic profiles of essential oils, since α-humulene is an important constituent of hop essential oil and may contribute to the aroma characteristics of hop inflorescences [87].

The variation observed in the present study may reflect differences among varieties and plant tissues. The present study observed differences in α-humulene abundance among varieties (Table 1) and plant tissues, as well as variety-specific differences in the accumulation of common sesquiterpenes. According to Herkenhoff et al. [14], genetic and environmental factors can contribute to variation in hop secondary metabolism, affecting the synthesis and accumulation of volatile compounds. Therefore, the differences observed in α-humulene abundance among varieties and tissues may reflect interactions between genotype and the environmental conditions experienced during cultivation. However, this interpretation represents a proposed explanation rather than an experimentally demonstrated effect in the present study.

The results obtained for the leaves further support the observed pattern. α-Humulene and β-caryophyllene were among the major tentatively identified compounds in the leaf essential oils across varieties and sampling periods (Table 2), consistent with profiles previously reported by Katsiotis et al. [33] and Stanius et al. [10].

Oxygenated sesquiterpenes, including caryophyllene oxide and humulene oxides, were also identified in the essential oils. The occurrence of these compounds is consistent with the presence of their corresponding sesquiterpene hydrocarbons, particularly β-caryophyllene and α-humulene, which were present at relatively high abundances in some of the samples. Oxidative conversion of sesquiterpene hydrocarbons is one possible pathway for forming these oxygenated derivatives. However, oxygenated sesquiterpenes may also arise through biosynthetic or enzyme-mediated processes in plant tissues. In *H. lupulus*, oxygenated sesquiterpenes identified as epoxides, including caryophyllene epoxide and humulene epoxides I and II, have been reported in glandular trichomes, demonstrating their occurrence in these specialized secretory tissues [91]. Nevertheless, their presence in glandular trichomes does not establish a specific enzymatic conversion of β-caryophyllene or α-humulene into their corresponding oxygenated derivatives. In addition, these compounds may be formed or modified during post-harvest handling [92], storage [32], or essential oil extraction, including hydrodistillation [93], as well as the comparison of methods for obtaining hop oil [83]. Therefore, the co-occurrence of β-caryophyllene and caryophyllene oxide, as well as α-humulene and humulene oxides, suggests a possible precursor–derivative relationship, but the present data do not allow the specific origin or formation pathway of these oxygenated sesquiterpenes to be established.

Phytol was present at relatively high relative abundance in some leaf essential oils, particularly those from the second sampling period, although it was not among the major constituents overall (Table 2). Its higher occurrence in leaves than in cones may be associated with differences in tissue composition and physiology. As the phytyl side chain of chlorophyll, phytol can be released during chlorophyll degradation [94,95], suggesting that differences in leaf physiological status, chlorophyll turnover, or sample processing may contribute to its variation among sampling periods. However, these mechanisms were not directly evaluated in the present study and therefore remain possible explanations.

Within this chemical class, farnesene provides an example of the variety-, tissue-, and cultivation-period-specific variation observed in the present study. This tentatively identified sesquiterpene was detected exclusively in the cones of Cascade and Columbus, whereas it was not detected in Nugget or Comet or in the leaf oils (Table 1 and Table 2). Notably, its abundance in Columbus varied markedly among cultivation periods, being 1.12% in the first period, not detected in the second, and increasing to 14.30% and 24.84% in the third and fourth periods, respectively. Thus, although farnesene occurred in both Cascade and Columbus, its abundance was not consistent across cultivation periods in Columbus, indicating substantial temporal variation in the essential oil profile of this variety. Farnesene has been associated with several ecological and physiological processes in plants, including responses to abiotic stresses such as high temperature and water deficit, as well as chemical signaling and interactions with insects [96]. Therefore, its occurrence in the cones of Cascade and Columbus, together with its marked variation among cultivation periods in Columbus, may reflect differences in variety- and/or environment-dependent responses. However, because the present data are correlational, the occurrence or accumulation of farnesene cannot be interpreted as evidence of a causal relationship with variety adaptation. Physiological and molecular studies would be required to determine whether farnesene accumulation is functionally associated with stress responses or environmental adaptation in these varieties.

A similar pattern was observed for β-pinene, although its occurrence was more dependent on cultivar and harvest period than that of farnesene. Srivastava et al. [97] reported that β-pinene emission may increase under combined stress conditions, particularly in response to elevated temperature and high light intensity. In the present study, β-pinene was tentatively identified in the cones of the second harvest of Cascade (28.59%) and in the second and third harvests of Comet (26.51% and 16.16%, respectively). It was also detected in the leaf essential oils only during the third harvest, with the highest relative abundances observed in Cascade (23.31%) and Columbus (26.23%) (Table 2). The occurrence and variation in β-pinene abundance across cultivars, tissues, and harvest periods may reflect differences in plant physiological responses to the environmental conditions prevailing during each developmental stage. Although this pattern is consistent with the stress-related increase in β-pinene reported by Srivastava et al. [97], the present results do not allow a direct causal association between β-pinene accumulation and cultivar adaptation. Further physiological studies would be necessary to determine whether its accumulation is specifically associated with responses to high temperature, light intensity, or other environmental factors.

Additional evidence from Brazilian cultivation systems further highlights the variability of the volatile profile of *H. lupulus* under different growing and post-harvest conditions. Almeida et al. [97] characterized the essential oils of 18 samples representing 13 *H. lupulus* varieties grown under tropical Brazilian conditions. In Cascade, Columbus, Comet, and Nugget, which were also evaluated in the present study, β-myrcene was the predominant constituent, with relative abundances ranging from 25.0 to 41.6%, followed by sesquiterpenes such as β-caryophyllene (2.8–13.0%) and β-farnesene (3.6–27.0%). α-humulene showed greater variability among samples and was detected predominantly in Cascade, reaching 13.8%. Similarly, Sabino et al. [7] reported β-myrcene as the predominant constituent in these four cultivars, with relative abundances of 51.82% in Cascade, 56.50% in Columbus, 56.80% in Comet, and 57.38% in Nugget. In contrast, β-myrcene was not tentatively identified in any cone or leaf samples in the present study.

The non-detection of β-myrcene in the present study, despite its high reported abundance in the same cultivars reported by Sabino et al. [7] and Almeida et al. [98], represents an important difference in the volatile profiles obtained under Brazilian growing conditions. This discrepancy may be interpreted not only in terms of post-harvest losses, but also in relation to the biosynthetic organization of terpene metabolism in *H. lupulus*. According to Wang et al. [99], hop glandular trichomes produce major volatile terpenes from common isoprenoid precursors, with a metabolic bifurcation occurring at the level of prenyl diphosphates. β-Myrcene is a monoterpene derived from geranyl diphosphate (GPP), whereas α-humulene and β-caryophyllene are sesquiterpenes derived from farnesyl diphosphate (FPP). In hop, HlMTS2 has been experimentally characterized as a myrcene synthase that converts GPP into β-myrcene, whereas HlSTS1 produces both α-humulene and β-caryophyllene from FPP [98].

Thus, although these compounds belong to the same general terpenoid metabolic network and share the upstream precursors isopentenyl diphosphate (IPP) and dimethylallyl diphosphate (DMAPP), their formation depends on different prenyl diphosphate pools and distinct mono- and sesquiterpene synthase activities.

Consequently, the predominance of α-humulene and β-caryophyllene observed in the present samples in this study, together with the absence of detectable β-myrcene (Table 1), may reflect differences in the relative contribution of the GPP- and FPP-derived branches of terpene biosynthesis. Environmental conditions during plant development may shift this balance by affecting terpene synthase expression or activity and the availability of their precursors. In this regard, Eriksen et al. [100] identified transcripts associated with volatile terpene biosynthesis in hop leaf lupulin glands, including myrcene and humulene synthases, and reported changes in the expression of several of these transcripts under heat and low-water stress. Therefore, the chemical profile observed in the present study may reflect not only the genetic background of the cultivars but also environmentally induced changes in the relative contribution of monoterpene- and sesquiterpene-derived pathways.

Nevertheless, a biosynthetic explanation alone is unlikely to account for the complete absence of β-myrcene in the analytical profiles, particularly because this compound is commonly reported as a major constituent of hop cone essential oils. A complementary explanation may involve post-harvest processing. As a small, acyclic monoterpene hydrocarbon, β-myrcene is highly volatile and susceptible to chemical transformation, making it particularly sensitive to drying, storage, and extraction conditions. Experimental studies have demonstrated changes in β-myrcene abundance during hop processing, including during pre-drying storage and drying [93].

In the present study, both leaves and cones were dried before extraction and then subjected to hydrodistillation for 180 min. Importantly, Katsiotis et al. [33] reported β-myrcene at concentrations of 1% or less in essential oils obtained from dried leaves of ten *H. lupulus* cultivars, whereas α-humulene and β-caryophyllene were the predominant constituents. More recently, Campos et al. [94] demonstrated that hydrodistillation time significantly affected the chemical composition of hop essential oil, including β-myrcene, with variation observed among extraction times of 60, 120, 180, and 240 min. Thus, when comparing the volatile profiles of hop samples, consider differences in plant tissue, environmental conditions, drying, and hydrodistillation conditions. The absence of β-myrcene in the present study may therefore result from the combined influence of these biological and processing factors, although the contribution of chromatographic conditions cannot be completely excluded.

Another aspect to consider when interpreting the observed differences in the chemical composition of essential oils is the edaphic characteristics of the *H. lupulus* cultivation site. The plants evaluated in this study were grown in soil derived from Caiuá Sandstone, a sandy-loam textured soil characterized by high sand content, low water retention and availability, low cation exchange capacity (CEC), and acidic pH. Soils derived from Caiuá Sandstone are known for low clay and organic matter content and, consequently, reduced cation retention capacity and lower buffering capacity; these characteristics can increase susceptibility to nutrient loss via leaching and make element availability in the soil solution more dynamic [101,102].

Although the lower water retention was partially offset by the irrigation used in the experiment, the soil’s low CEC and acidic pH may have influenced nutrient availability dynamics throughout the crop cycle. In this context, soil pH differentially influences mineral elements. At the same time, acidic conditions may favor the availability of certain micronutrients, such as Fe, Mn, Zn, and Cu; they can reduce the availability of other elements, such as P and Mo, and increase the risk of Al and Mn toxicity. Thus, beyond nutritional limitations, the soil’s chemical characteristics may have altered the proportions and timing of nutrients actually available to the plants, thereby affecting physiological processes related to growth, cone formation, and specialized metabolism. This possibility is particularly relevant for *H. lupulus*, given that edaphic and nutritional factors have been reported to contribute to regional differences in hop quality and chemical composition. Studies on Cascade and Mosaic varieties have shown that cultivation environment characteristics, including soil pH and Zn, S, and Mn concentrations, are significantly associated with various hop quality attributes, highlighting the influence of edaphic conditions on the crop’s chemical performance [103].

Furthermore, under Brazilian conditions, nutrient availability has also been shown to influence *H. lupulus* production: Lagos et al. [104] observed a linear increase in dry matter production and essential oil yield in the Chinook cultivar in response to increasing nitrogen doses. At the same time, uptake studies demonstrate differences among hop cultivars regarding nitrogen acquisition and utilization efficiency [104,105].

Because terpenoids are products of a highly regulated metabolic network whose activity depends on precursor availability, plant physiological status, and interactions with environmental factors, changes in nutritional status may indirectly affect carbon allocation and specialized metabolite synthesis [106].

Differences between the chemical profiles observed in the present study and those reported by Sabino et al. [7] and Almeida et al. [98] may reflect differences in climate, growing season, genetic material, and cultivation conditions, including soil characteristics. Although the soil in the present study was analyzed and appropriately corrected before cultivation, the plants were grown on Caiuá Sandstone, characterized by low cation exchange capacity (CEC) and acidity. Differences in edaphic conditions among studies may have contributed to the variation in essential oil composition observed among the evaluated plant materials. However, the present study was not designed to determine the specific contribution of soil properties to essential oil composition. Therefore, these differences should be interpreted as chemical variation under the specific genetic and cultivation conditions evaluated, rather than as evidence of a stable regional or variety-specific chemotype.

From an agronomic perspective, hop cultivation was feasible under the subtropical conditions of northwestern Paraná, although the varieties differed in their performance across cultivation periods. Columbus showed the highest performance among the varieties evaluated under the experimental conditions, indicating its potential for further investigation. However, this result alone does not demonstrate superior adaptation to the region, as adaptation and stability require evaluation across multiple cultivation cycles and independent environments.

Overall, the findings provide a basis for further investigation of hop production in northwestern Paraná. Future studies should evaluate multiple cultivation cycles and environments while integrating agronomic, edaphic, climatic, and chemical variables, as well as irrigation and fertilization practices. Such approaches would help determine the consistency of the responses observed here and clarify the factors associated with variation in terpenoid composition. This broader validation will be important for assessing the potential of specific varieties for the region without prematurely attributing a stable regional chemical profile or identity to the raw material.

## 3. Materials and Methods

### 3.1. Characterization of the Experimental Area

The experiment was conducted in a 1000 m^2^ experimental area belonging to the State University of Maringá (UEM), Umuarama Regional Campus, located at 23°47′22.37″ S, 53°15′29.98″ W, at an altitude of 401 m [107]. According to the Köppen classification (Peel et al. [13], the region has a humid subtropical mesothermal (Cfa) climate characterized by hot summers and rare winter frosts; rainfall tends to be concentrated in summer, although there is no defined dry season. Mean temperatures exceed 22 °C in the warmest months and fall below 18 °C in the coldest months, with a mean annual precipitation of 1500 mm [108].

The soil in which the hop crop was established is derived from Caiuá Sandstone and is classified as a Dystroferric Red Latosol, characterized by a sandy-loam surface layer with low clay and organic matter contents [109].

### 3.2. Crop Establishment

A total of 204 seedlings representing four hop varieties, 51 each of Cascade, Columbus, Nugget, and Comet, were acquired from the company Lupulo Ouro Verde (Rodovia José Budny, 149, Vila Nova, Içara, Santa Catarina State, Brazil). The seedlings were delivered in plastic nursery bags (polybags). Planting was carried out with a spacing of 3.0 m between rows and 1.0 m between plants (0.50 m), utilizing a double-stem training system with the main stem supported, in accordance with crop management recommendations, and a support structure designed to prevent shading between plants.

### 3.3. Treatments and Experimental Design

**Figure 13:**
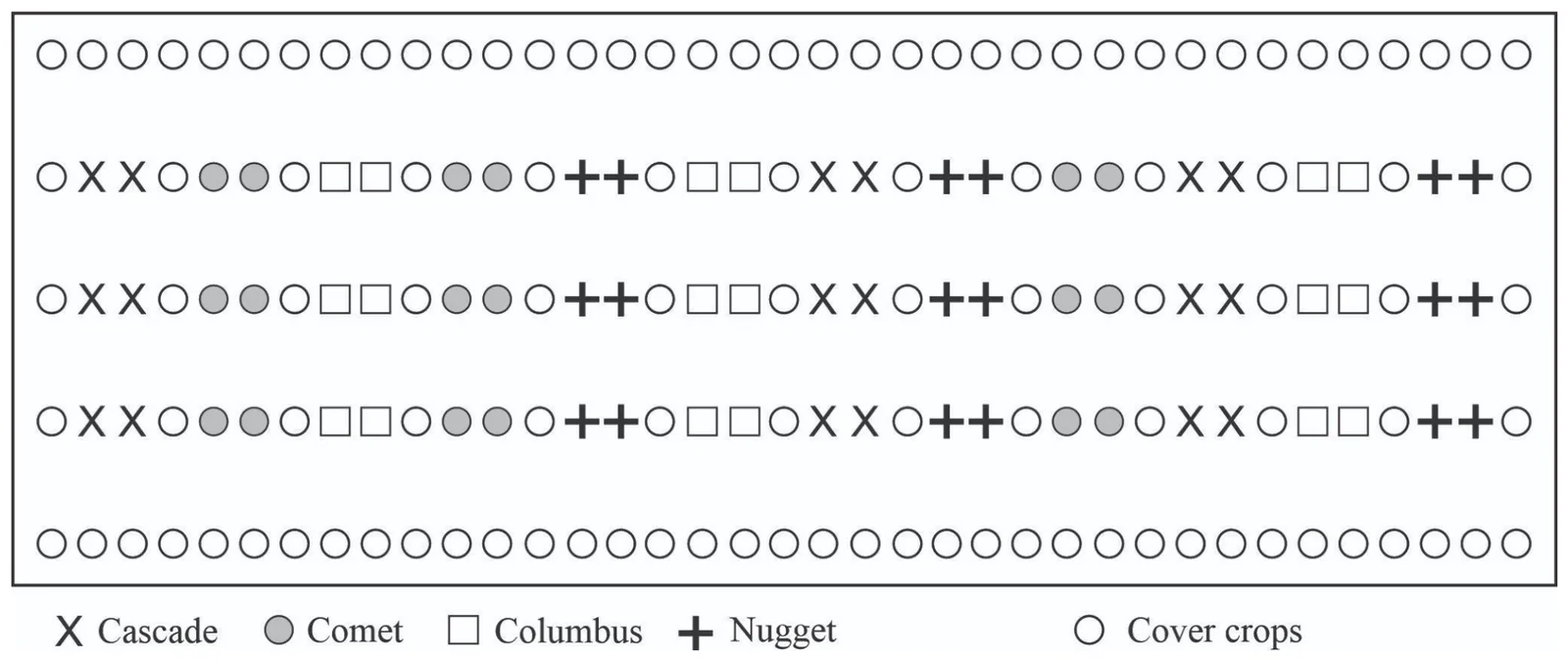
Layout of the experimental area for *Humulus lupulus* cultivation.

For each variety, 18 plants were established and distributed into three blocks of six plants each, resulting in three field replications per variety. For biomass measurements, the field block was considered the experimental unit, resulting in three field replications per variety. Additional plants of the same varieties were planted around the experimental area as border plants to minimize edge effects (Figure 13).

At each harvest, the cones and leaves from the 18 plants of each cultivar were collected and pooled, resulting in a single composite sample for each cultivar and harvest. The three field blocks were therefore not processed or analyzed separately for cone yield, leaf yield, or essential oil yield. For essential oil analysis, aliquots of the composite samples were subsequently subjected to triplicate extraction, which represented technical replicates rather than independent field or biological replicates.

### 3.4. Soil Preparation, Fertilization, and Crop Management

Before planting in 2022, and before the beginning of vegetative growth in the subsequent growing cycles (2023 and 2024), soil samples were collected at depths of 0–20 and 20–40 cm for chemical characterization. The evaluated properties included pH determined in CaCl_2_, phosphorus (P), organic matter (O.M.), calcium (Ca), potassium (K), magnesium (Mg), aluminum (Al), cation exchange capacity (CEC), and base saturation (V). The results of the soil analysis are detailed in Table S1.

Based on the results, fertilization and planting recommendations for the hop crop were implemented following the guidelines of [109]. Soil acidity correction was performed based on the base saturation level obtained from the chemical analysis, raising it to 70% where necessary [2].

Before planting, raised beds were prepared along the planting rows by tilling the soil to facilitate root system development, as recommended by Spósito et al. [2]. Planting holes approximately 20 cm deep were prepared in the beds according to the designated plant spacing.

Top-dressing fertilization was performed using single superphosphate (208 g), potassium chloride (5 g), and urea (22 g), according to Teixeira, Aquino, and Macedo [110]. Fertilizer applications were split according to the phenological stages of the hop crop and the proportions recommended by the same authors.

The hop cultivars were planted on 2 November 2022, and four harvests were conducted in January 2023, April 2023, December 2023, and May 2024. Harvesting was performed when the cones reached maturity, according to the criteria described by Spósito et al. [2].

### 3.5. Meteorological Data Collection

Meteorological data on air temperature (maximum, mean, and minimum; °C), mean relative humidity (%), and accumulated rainfall (mm) were obtained from an automatic weather station installed near the experimental area. Air temperature and relative humidity were measured using an HMP155A thermo-hygrometer sensor (Vaisala Inc., Helsinki, Finland), while rainfall was recorded using a TE525MM tipping-bucket rain gauge (Texas Electronics Inc., Dallas, TX, USA).

The sensors were connected to a CR3000 datalogger (Campbell Scientific Inc., Logan, UT, USA), programmed to take readings every second and store mean values for continuous variables and accumulated rainfall at 15-min intervals. Before installation, the sensors were calibrated according to the manufacturers’ specifications, and periodic maintenance ensured data quality and reliability.

For the analysis of temperature variation at different times of the day, records obtained at 09:00, 15:00, and 21:00 were grouped by month, and the corresponding mean values were calculated. Table S2 presents these data.

### 3.6. Irrigation System and Management

A drip irrigation system was used, featuring drip tapes with emitters spaced 0.15 m apart and a nominal flow rate of 1.6 L h^−1^. The system was adjusted to operate at a working pressure of 15 m H_2_O. Before installation, water application efficiency and uniformity were evaluated using the methodology proposed by Karmeli and Keller [111], resulting in a Christiansen’s Uniformity Coefficient (CUC) of 92%.

Irrigation management was based on tensiometers installed at depths of 0.20 and 0.40 m. Readings were taken every 2–3 days using a digital tensiometer, and the irrigation requirement to restore soil moisture to field capacity was calculated based on the mean soil matric potential (ψm).

Based on the tensiometer readings, soil water content was estimated using the Van Genuchten [112] equation, with the parameters presented in Table 3. Model parameters were fitted using RETC software (Version 6.02) developed by the US Salinity Laboratory–USDA Van Genuchten [112], utilizing data pairs obtained from the soil water retention curve.

### 3.7. Cone and Leaf Yield

After each harvest, cones and leaves from the 18 plants of each cultivar were separated from the stems and pooled to obtain a single composite sample per cultivar and harvest. The fresh weight of cones and leaves was determined by weighing the respective composite samples. The plant material was then dried on trays at ambient temperature (32 °C) for approximately 7 days, until a constant dry weight was reached (Table S3). The dried material was subsequently stored in sealed kraft paper bags at room temperature for 5 days before hydrodistillation. The interval between harvest and the beginning of drying was approximately 4 h. Yield was expressed in kilograms (kg) of fresh weight (FW) and dry weight (DW), as shown in Table S3.

### 3.8. Essential Oil Extraction

Essential oil extraction was performed using dried leaves and cones from the different varieties evaluated. The plant material used for extraction consisted of composite samples obtained by pooling the material collected from the 18 plants of each variety at each harvest. Thus, each composite sample represented the experimental unit for essential oil extraction. The plant material was weighed separately, with 100 g of leaves used for each of the four varieties. For the cones, different masses were used according to the variety: 50 g for Cascade and Columbus and 20 g for Comet and Nugget. No cones were obtained from the Comet variety in the fourth harvest. After weighing, the plant material was homogenized with distilled water using an industrial blender (SPOLU, model SPL-023ECO, Itajobi, SP, Brazil). The resulting mixture was transferred to a distillation flask and hydrodistilled for 3 h using a Clevenger-type apparatus (Carlabor, São Paulo, SP, Brazil) according to Flythe [113]. After distillation, the essential oil was recovered from the distillation apparatus using *n*-Hexane was dried over anhydrous sodium sulfate to remove residual moisture. The resulting solution was transferred to pre-weighed open glass vials and placed at −4 °C at the door of a refrigerator to allow the *n*-hexane to evaporate. The vials were kept open under these conditions for approximately one month, until the solvent had evaporated, before gravimetric determination of the recovered essential oil. The mass of the recovered essential oil was determined by weighing the vial containing the oil after solvent evaporation and subtracting the tare mass of the empty vial. Three extraction replicates were performed using aliquots of the same composite sample for each variety, plant material, and harvest. These replicates were considered technical replicates and were not treated as independent biological replicates.

### 3.9. Essential Oil Yield (%)

Essential oil yield (%) was calculated as the ratio between the mass of essential oil obtained (g) and the mass of dried plant material used for extraction (g), multiplied by 100. For each cultivar, plant material, and harvest, three technical extraction replicates were performed using aliquots of the same composite sample. The mean and standard deviation of the technical replicates were calculated for descriptive purposes. Because the three replicates originated from the same composite sample, they were not considered independent biological replicates and were not used as independent observations for inferential statistical comparisons.

### 3.10. Chemical Characterization of Essential Oils by Gas Chromatography–Mass Spectrometry (GC-MS)

The essential oils obtained from the leaves and cones of *Humulus lupulus* samples were diluted 1:10 (*v*/*v*) with dichloromethane, corresponding to a final concentration of 0.1 mg/mL, and analyzed by gas chromatography coupled with mass spectrometry (GC–MS) using an Agilent 7890B gas chromatograph coupled to a 5977A mass selective detector (MSD) (Agilent Technologies, Santa Clara, CA, USA). Separation was performed on an HP-5MS UI capillary column (30 m × 0.25 mm × 0.25 μm). The oven temperature was initially set at 50 °C and held for 1 min, then increased at 10 °C/min to 250 °C, followed by an increase at 25 °C/min to 300 °C, which was maintained for 10 min. Helium was used as the carrier gas at a flow rate of 1.0 mL/min.

The injector temperature was set at 250 °C, and 2 μL of each sample was injected in split mode at a split ratio of 20:1. The transfer line, ion source, and quadrupole temperatures were maintained at 290, 230, and 150 °C, respectively. Compounds were ionized by electron ionization (EI) at 70 eV, and mass spectra were acquired in full-scan mode over an *m*/*z* range of 30–600, with a solvent delay of 3 min [83].

A single sample per variety and harvest was analyzed by GC–MS. Compounds were identified by comparing their mass spectra with those in the NIST 11.0 spectral library and their calculated retention indices (RI), determined using a homologous series of n-alkanes (C7–C40), with literature values [114]. A compound was identified when the library match was ≥90% and the calculated RI was within [7] RI units of the corresponding literature value. The relative abundance of each identified compound was calculated as the percentage of its chromatographic peak area relative to the total integrated peak area. Therefore, the values reported in Table 1 and Table 2 represent relative chromatographic abundances and should not be interpreted as absolute concentrations or quantitative determinations.

### 3.11. Multivariate Statistical Analysis

To investigate patterns of variability, similarity, and correlation in the chemical composition of essential oils extracted from the cones and leaves of *Humulus lupulus* cultivars Cascade, Columbus, Comet, and Nugget, multivariate and univariate statistical analyses were performed. Multivariate analyses included Principal Component Analysis (PCA), Hierarchical Cluster Analysis (HCA), and Pearson correlation analysis and were conducted using R software (version 4.3.2; R Core Team). The analyses were performed separately for the compounds and chemical classes identified in cones and leaves.

The statistical analyses were based on the relative percentages of the compounds identified in Table 1 and Table 2. Only compounds with a content ≥ 6.0% in cones and in leaves in at least one of the analyzed samples were included in the statistical matrices. The data were organized into sample × variable matrices, with samples as observations and compounds or chemical classes as variables.

Before the multivariate analyses, the availability of essential oil samples was verified for each cultivar and cultivation cycle. The Comet 4C cone sample was excluded from the multivariate analyses because an insufficient amount of cones was obtained during the fourth cultivation cycle to allow essential oil extraction. Thus, no essential oil sample was available for this cultivar × cultivation cycle combination, and Comet 4C was treated as a missing sample at the sample level rather than as a sample with zero compound concentrations. Accordingly, we did not include this missing sample in the PCA, HCA, or Pearson correlation analyses.

For samples in which essential oil was successfully obtained, values reported as trace amounts or not detected for individual compounds were treated as zero when constructing the statistical matrices. Variables exhibiting the same value across all included samples (zero variance) were excluded before the multivariate analyses because they did not contribute to sample discrimination.

For Principal Component Analysis (PCA), the data were mean-centered and scaled to unit variance using the prcomp function in R. Hierarchical Cluster Analysis (HCA) used the same standardized matrices, with Euclidean distance as the similarity measure and Ward’s method (ward.D2) for cluster formation. Pearson correlation analyses were performed separately for cones and leaves and were conducted at two levels: individual chemical compounds and chemical classes. Pearson correlation coefficients (rrr) were calculated for each pair of variables using the samples available for the respective plant organ and data level. For each pairwise correlation, both the raw *p*-value and the Benjamini–Hochberg-adjusted *p*-value were calculated to account for multiple comparisons. Associations with an adjusted *p*-value below 0.05 were considered statistically significant. The magnitude of the correlations was classified according to the absolute value of *r* as weak, moderate, strong, or very strong, and the direction was classified as positive or negative according to the sign of *r*. The PCA and HCA analyses and their graphical representations were generated using the factoextra package in R, whereas the Pearson correlation matrices and heatmaps were generated using the corrplot package in R.

Essential oil yield (%) was calculated for each cultivar and cultivation cycle. The results were expressed as mean ± standard deviation and presented graphically. Because essential oil yield was not subjected to inferential statistical analysis, no analysis of variance or multiple comparisons among means was performed.

## 4. Conclusions

The present study demonstrated the feasibility of cultivating *Humulus lupulus* L. under the subtropical edaphoclimatic and management conditions evaluated in northwestern Paraná, Brazil. The evaluated cultivars showed distinct agronomic and chemical responses across the cultivation cycles. Cascade and Columbus exhibited the best overall agronomic performance under the conditions tested, whereas Nugget and Comet showed higher essential oil yields in cones. In leaves, Nugget, Cascade, and Comet presented the highest essential oil yields, indicating that vegetative tissues may also represent a relevant source of volatile compounds.

Chemical characterization revealed a predominance of sesquiterpene hydrocarbons in most samples, particularly α-humulene and β-caryophyllene. In contrast, oxygenated sesquiterpenes and the high abundance of phytol in leaves highlighted differences between reproductive and vegetative tissues. The absence or low detectability of β-myrcene, commonly reported as a major constituent of hop essential oils, indicates that the volatile profiles observed in this study differed from those commonly reported for *H. lupulus*. However, the contribution of specific environmental, seasonal, or post-harvest factors to these differences cannot be separated under the experimental conditions evaluated.

Multivariate analyses further revealed differences in essential oil profiles among cultivars and harvest periods, indicating that both cultivar and harvest period were associated with the observed chemical differentiation. Nevertheless, because the study was conducted at a single location and environmental and management conditions varied simultaneously among cultivation cycles, these results should not be interpreted as evidence of a specific genotype × environment interaction or as demonstrating the individual effects of particular environmental factors. Further multi-location and multi-year studies under controlled management conditions are warranted to disentangle the effects of genotype, environment, seasonality, and their interactions on the agronomic performance and chemical composition of *H. lupulus*.

## Figures and Tables

**Table 1:** Chemical composition and relative peak area (%) of compounds identified in essential oils from cones of the Cascade, Columbus, Comet, and Nugget varieties of *Humulus lupulus* across four cultivation cycles.

Nº	TR	Compounds	Cas 1	Cas 2	Cas 3	Cas 4	Col 1	Col 2	Col 3	Col 4	Com 1	Com 2	Com 3	Com 4	Nug 1	Nug 2	Nug 3	Nug 4	RI Cal	RI Lit	Ref. Lit.
1	3.194	α-Pinene	-	-	-	-	-	-	-	-	3.94	0.17	-	-	-	-	-	-	911	917	[35]
2	3.387	Camphor	-	-	-	-	0.18	-	-	-	-	0.43	-	-	1.64	-	3.23	-	935	933	[35]
3	3.564	β-Pinene	3.61	28.59	-	-	2.88	-	0.91	0.78	3.41	26.51	16.16	-	-		-	0.28	955	957	[36]
4	4.211	β-Cimene	3.04	-	-	0.88	-	-	-	-	0.34	0.59	0.57	-	-	-	-	-	1017	1023	[37]
5	4.423	Eucalyptol	1.56	1.05	-	0.33	1.28	-	-	0.37	0.66	0.70	-	-	2.02	-	-	1.05	1035	1033	[38]
6	5.233	Fenchone	5.46	-	-	0.40	-	-	-	0.34	0.19	0.31	2.29	-	-	-	-		1089	1089	[39]
7	5.379	linalol	1.17	-	-	0.55	-	-	-	0.35	0.78	0.59	-	-	-	-	-	1.07	1099	1100	[40]
8	7.423	Carvone	3.55	0.74	-	0.90	-	-	-	0.85	1.28	1.35	-	-	6.57	-	2.54	2.52	1244	1244	[41]
9	8.006	2- undecanone	-	0.85	0.87	0.44	-	-	-	1.97	0.59	0.85	1.21	-	1.84	-	2.83	0.62	1285	1283	[42]
11	8.243	α-limonene diepoxide	-	-	-	4,12	-	-	-	-	2.02	2,56	-	-	-	-	-	-	1301	1295	[43]
12	8.492	Methyl geraniate	-	-	-	1.79	-	-	-	-	1.23	1.55	-	-	-	-	-	-	1320	1326	[40]
13	9.126	α-Copaene	-	-	-	4.60	-	-	-	1.20	-	-	-	-	-	-	-	0.63	1369	1367	[44]
14	9.270	Geranyl acetate	-	-	-	1.38	1.60	-	1.42	1.88	0.84	0.81	-	-	-	-	-	-	1380	1380	[45]
15	9.685	Caryophyllene	-	12.43	8.2	11.57	13.3	13.5	12.4	12.49	3.13	12.4	19.54	-	4.44	22.5	3.50	13.58	1432	1431	[46]
16	9.985	α-bergamotene	-	-	-	1.07	0.94	1.24	1.33	2.56	-	-	-	-	-	-	-	-	1436	1436	[46]
17	10.112	Humulene	-	2.49	18.79	25.96	39.5	44.9	33.6	3.62	0.48	1.40	0.87	-	8.63	57.4	2.34	30.49	1447	1447	[47]
18	10.244	β-Farnesene	12.18	18.81	7.8	1.24	1.12	-	14.3	24.84	-	-	-	-	-	-	-	-	1457	1459	[40]
19	10.433	4,5-di-epi- aristocholene	-	-	-	1.44	-	-	-	-	1.42	1.42	2.81	-	-	-	-		1472	1471	[48]
20	10.507	γ-muurolene	2.36	1.81	1.81	2.41	2.18	2.34	1.44	6.35	3.05	2.21	1.08	-	-	-		5.37	1478	1474	[49]
21	10.647	β-Eudesmene	-	5.39	0.77	3.24	7.21	6.71	7.95	-	14.48	9.42	11.83	-	-	-		1.73	1489	1489	[40]
22	10.684	α-Selinene	7.22	8.59	6.88	4.49	4.86	6.33	-	3.22	7.19	8.48	-	-	-	3.44	1.12	6.76	1496	1494	[50]
23	10.752	δ-Cadinene	5.03	1.74	1.47	2.13	2.56	3.24	7.20	2.85	-	0.83	2.03	-	1.25	11.2	4.34	2.55	1496	1493	[36]
24	10.898	Geranyl isobutyrate	-	-	-	1.38	1.95	2.27	1.70	1.42	1.45	1.79	-	-	-	-	-	-	1509	1514	[51]
25	10.972	γ-Cadinene	-	-	6.09	1.11	1.76	1.63	-	1.49	0.73	1.00	0.46	-	-	-	-	1.32	1509	1511	[49]
26	11.074	β-Cadinene	-	2.66	-	1.07	-				1.15	3.21	3.35	-	-	-	-	2.98	1529	1530	[52]
27	11.320	Caryophyllene oxide	7.81	4.40	4.16	1.25	3.45	1.55	10.67	1.57	26.23	7.07	12.61	-	19.27	-	4.76	2.84	1545	1552	[38]
28	11.829	Globulol	-	-	6.40	2.00	-	-		5.36	3.95	6.57	3.96	-	-	-		-	1582	1583	[53]
29	12.112	Humulene oxide II	15.52	-	16.91	1.44	4.31	3.02	0.93	1.43	2.82	0.51	7.72	-	34.55	-	6.83	1.72	1612	1610	[54]
30	12.120	Isoaromadendrene epoxide	-	-	-	1.78	-	-	-	-	3.52	0.78	-	-	-	-	-	0.56	1613	1612	[55]
31	12.314	α-acorenol	12.42	-	1.56	9.41	8.00	11.21	4.80	18.73	0.73	0.55	4.80	-	-	-	-	17.7	1631	1630	[56]
32	12.362	T-cadinol	-	-	-	0.47	-	-	-	-	-	-	-	-	5.58	5.39	4.46	-	1635	1635	[57]
33	12.366	Cubenol	-	-	-	1.18	-	-	-	1.57	2.64	0.84	-	-	-	-	-	-	1635	1636	[47]
34	12.407	β-Eudesmol	-	8.03	2.91	1.34	-	-	-	-	6.2	0.74	5.56	-	-	-	-	-	1639	1645	[58]
35	12.473	Turmerone	10.34	-	10.74	-	-	-	-	-	-	-	-	-	7.43	-	10.00	-	1645	1649	[59]
36	12.795	Aromadendrene oxide-(I)	-	-	-	1.76	-	-	-	1.00	0.68	0.42	0.61	-	-	-	-	1.26	1673	1672	[60]
37	13.522	Farnesol	-	-	3.18	4.03	-	-	-	2.13	2.11	2.66	-	-	2.5	-	5.43	2.11	1740	1747	[61]
38	16.631	n.i	-	-	-	-	-	-	-	-	-	-	-	-	-	-	16.3	-	1952	-	
39	16.651	Geranyl linalool	-	-	-	0.75	1.70	-	1.60	0.87	2.63	0.29	2.14	-	-	-	-	-	2054	2046	[62]
40	17.166	Hulupone	-	-	1.45	-	-	-	-	-	-	-	-	-	-	-	13.16	-	2109	2103	[63]
41	17.208	Phytol	-	0.77	-	0.77	0.93	1.87	-	0.49	-	-	-	-	-	-	-	0.92	2114	2115	[46]
42	18.346	Cohumulone	-	-	-	-	-	-	-	-	-	-	-	-	4.27	-	6.80	-	2267	NF	
43	21.208	n.i	7.82	-	-	-	-	-	-	-	-	-	-	-	-	-	6.67	-	2709		
44	21.433	n.i	-	-	-	-	-	-	-	-	-	-	-	-	-	-	5.00	-	2785		
Hydrocarbon Monoterpenes			6.65	28.59	-	0.88	3.06	-	0.91	0.78	7.69	27.70	16.73	-	1.64	-	3.23	0.28			
Oxygenated Monoterpenes			11.74	1.79	-	10.85	1.28	-	-	1.91	4.93	5.51	2.29	-	8.59	-	2.54	4.64			
Hydrocarbon Sesquiterpenes			26.79	53.92	51.81	60.33	73.45	79.94	77.95	58.62	31.63	40.37	41.97	-	14.32	94.54	11.3	65.41			
Oxygenated Sesquiterpenes			46.09	12.43	45.86	24.66	15.76	15.78	16.40	31.79	48.88	20.14	35.26	-	69.33	5.39	31.48	26.1			
Diterpenes			-	0.77	-	1.52	2.63	1.87	1.60	1.36	2.63	0.29	2.14	-	4.27	-	-	0.93			
Ketones Terpenoid Ester			-	0.85	0.87	0.44	-	-	-	1.97	0.59	0.85	1.21	-	1.84	-	2.83	0.62			
			-	-	-	4.55	3.55	2.27	3.12	3.30	3.52	4.15	-	-	-	-	-	-			
Alpha and Beta Acids Not identified			-	-	1.45	-	-	-	-	-	-	-	-	-	4.27	-	19.96	-			
			7.82	-	-	-	-	-	-	-	-	-	-	-		-	27.97	-			
			**99.09**	**98.35**	**99.99**	**98.68**	**99.73**	**99.86**	**99.98**	**99.73**	**99.87**	**99.01**	**99.6**	**-**	**99.99**	**99.93**	**99.31**	**98.06**			

Compounds are listed in order of elution from the HP-5MS column. Retention indices were calculated using a homologous series of *n*-alkanes (C7–C40) analyzed on an HP-5MS column. Compounds were tentatively identified at confidence level 2 according to the Metabolomics Standards Initiative, based on comparison of their mass spectra with the NIST 11.0 spectral library and their calculated retention indices (RI calc.) with literature values (RI lit.). Unless otherwise indicated, compound identities were not confirmed using authentic reference standards. Relative peak area (%): percentage of the total chromatographic peak area represented by each compound; RI calc.: calculated retention index; RI lit.: retention index reported in the literature; RT: retention time; (–): compound not detected; n.i.: not identified; NF: not found. Cas1, Col1, Com1, and Nug1 correspond to the 1st cultivation (November 2022–January 2023); Cas2, Col2, Com2, and Nug2 correspond to the 2nd cultivation (February–April 2023); Cas3, Col3, Com3, and Nug3 correspond to the 3rd cultivation (September–December 2023); and Cas4, Col4, Com4, and Nug4 correspond to the 4th cultivation (January–May 2024). Cas: Cascade; Col: Columbus; Com: Comet; Nug: Nugget.

**Table 2:** Chemical composition and relative peak area (%) of compounds identified in essential oils from leaves of the Cascade, Columbus, Comet, and Nugget varieties of *Humulus lupulus* across four cultivations.

Nº	TR	Compounds	Cas 1	Cas 2	Cas 3	Cas 4	Col 1	Col 2	Col 3	Col 4	Com 1	Com 2	Com 3	Com 4	Nug 1	Nug 2	Nug 3	Nug 4	RI Calc	RI Lit	Ref. Lit.
1	3.896	β-Pinene		-	23.3	-	-	0.38	26.2	1.11	-	-	14,5	-	-	-	8,75	-	950	957	[36]
2	4.545	Limonene	-	-	-	-	1.93	0.18	-	5.13	-	-	-	-	1.6	-	-	-	1043	1044	[49]
3	9.181	α-Copaene	-	-	-	0.98	2.45	0.44	-	1.51	1.05	0.96	1.43	0.94	2.38	0.79	-	-	1373	1378	[64]
4	9.685	Caryophyllene	14.70	9.97	22.9	7.98	17.15	8.66	30.1	7.34	34.77	29.70	24.50	26.95	14.43	10.00	18.76	3.13	1432	1431	[46]
5	9.800	β-Copaene	2.55	2.43	-	-	-	-	-	2.56	3.86	4.05	-	-	2.66	0.85	-	5.68	1457	1459	[65]
6	10.112	Humulene	27.74	13.1	30.5	13.5	30.91	18.9	43.5	15.4	7.75	7.39	3.15	6.27	23.42	19.19	28.29	5.74	1472	1472	[66]
7	10.446	γ-Muurolene	2.39	-	-	2.01	2.55	2.56	-	1.93	-	-	-	3.58	7.11	1.76	9.67	3.20	1473	1474	[49]
8	10.624	α-Muurolene	-	2.81	-	4.01	1.69	2.35	-	3.69	-	-	-	-	0.84	3.48	-	2.07	1487	1481	[67]
9	10.658	β-Eudesmene	3.78	4.07	-	3.20	3.00	2.55	-	2.64	7.78	7.74	13.5	6.95	2.97	2.35	-	-	1489	1489	[40]
10	10.761	α-Selinene	-	3.90	7.36	2.97	5.06	3.01	-	4.60	9.68	9.93	17.8	10.2	4.22	3.43	-	1.56	1497	1497	[68]
11	10.972	γ-Cadinene	2.36	1.91	-	1.92	2.13	1.59	-	1.97	2.41	2.66	3.26	2.44	5.53	3.29	-	3.97	1515	1514	[69]
12	11.072	δ-Cadinene	3.60	3.09	-	2.51	3.96	3.50	-	1.94	4.87	5.29	1.61	3.98	11.28	4.69	12.64	4.05	1524	1524	[47]
13	11.081	β-Guaiene	-	-	-	-	-	-	-	-	2.95	4.85	-	2.19	-	-	-	1.68	1525	1529	[70]
14	11.241	γ-Selinene	-	-	-	-	-	-	-	-	2.65	0.82	0.96	4.19	-	-	-	-	1539	1532	[41]
15	11.808	Caryophyllene oxide	11.24	5.40	8.97	13.3	4.33	8.08	-	15.4	8.38	9.47	3.95	8.97	4.86	2.57	8.07	45.00	1586	1589	[40]
16	11.882	Humulene epoxide II	13.21	5.05	6.77	8.58	5.35	10.6	-	7.24	1.92	2.97	4.89	8.36	6.87	3.29	-	9.71	1592	1591	[71]
17	12.221	cubenol	2.56	1.60	-	0.54	0.99	0.56	-	1.12	2.57	1.57	-	2.10	5.76	1.33	-	-	1622	1623	[72]
18	12.314	α-acorenol	-	-	-	2.12	-	-	-	3.75	0.76	0.69	-	2.35	-	-	-	5.88	1622	1623	[72]
19	12.407	β-Eudesmol	-	-	-	2.00	-	-	-	-	-	0.46	2.09	8.19	-	-	-	-	1639	1645	[59]
20	12.617	α-Cadinol	1.18	-	-	-	1.38	5.39	-		0.45	0.83	3.8	-	-	-	-	-	1650	1650	[49]
21	12.686	α-Eudesmol	2.33	2.64	-	9.27	-	-	-	-	6.43	5.99	4.52	-	-	-	-	-	1664	1670	[73]
22	15.333	2-Pentadecanone	-	-	-	9.61	2.95	7.56	-	7.53	0.82	0.71	-	1.22	-	4.97	-	5.03	1716	1717	[74]
23	15.335	Hexahydrofarnesyl acetone	1.72	5.30	-	-	-	-	-	2.01	-	-	-	-	0.86	1.18	-	3.30	1917	1915	[75]
24	17.166	Phytol	10.2	37.71	-	12.8	14.15	22.85	-	11.8	0.86	3.91	-	1.00	4.54	36.62	13.62	-	2109	2105	[76]
	Hydrocarbon Monoterpenes		-	-	23.31	-	1.93	0.56	26.23	6.24	-	-	14.50	-	1.60	-	8.75	-			
	Hydrocarbon Sesquiterpenes		57.12	41.26	60.75	39.05	68.90	43.55	73.57	43.6	77.77	73.39	66.21	67.69	74.84	48.83	69.36	31.08			
	Oxygenated Sesquiterpenes		30.52	12.05	15.74	35.85	12.05	24.64	-	27.51	20.51	21.98	19.25	29.97	17.49	7.19	8.07	60.59			
	Diterpenes		10.20	37.71	-	12.80	14.15	22.85	-	11.80	0.86	3.91	-	1.00	4.54	36.62	13.62	-			
	Ketones		1.72	5.30	-	9.61	2.95	7.56	-	9.54	0.82	0.71	-	1.22	0.86	6.15	-	8.33			
	**Total identified**		**99.56**	**98.96**	**99.80**	**97.31**	**99.98**	**99.17**	**99.80**	**98.71**	**99.96**	**99.99**	**99.96**	**99.88**	**99.33**	**99.79**	**99.80**	**100.00**			

Compounds are listed in order of elution from the HP-5MS column. Retention indices were calculated using a homologous series of *n*-alkanes (C7–C40) analyzed on an HP-5MS column. Compounds were tentatively identified at confidence level 2 according to the Metabolomics Standards Initiative, based on comparison of their mass spectra with the NIST 11.0 spectral library and their calculated retention indices (RI calc.) with literature values (RI lit.). Unless otherwise indicated, compound identities were not confirmed using authentic reference standards; Relative peak area (%): percentage of the total chromatographic peak area represented by each compound; (–): compound not detected. Cas1, Col1, Com1, and Nug1 correspond to 1st Cultivation (November 2022–January 2023); Cas2, Col2, Com2, and Nug2 correspond to 2nd Cultivation (February–April 2023); Cas3, Col3, Com3, and Nug3 correspond to 3rd Cultivation (September–December 2023) and Cas4, Col4, Com4, and Nug4 correspond to 4th Cultivation (January–May 2024). Cas: Cascade; Col: Columbus; Com: Comet; Nug: Nugget.

**Table 3:** Saturated (θs) and residual (θr) soil water contents and empirical parameters (α, n, and m) of the van Genuchten model [110].

Soil Layer (cm)	θs (m^3^ m^−3^)	θr (m^3^ m^−3^)	α (k Pa^−1^)	n	m
0–20	0.2320	0.1057	0.3237	1.5550	0.3569
20–40	0.3560	0.1611	0.3189	1.5387	0.3501

## Data Availability

The datasets generated during and/or analyzed during the current study are available from the corresponding author upon reasonable request.

## Supplementary Materials

The following supporting information can be downloaded at: https://www.mdpi.com/article/10.3390/molecules31203605/s1, Table S1: Soil chemical properties measured at two soil depths before and during the hop cultivation period in 2022, 2023, and 2024; Table S2: Monthly mean temperature recorded at the experimental site at 09:00, 15:00, and 21:00 from November 2022 to May 2024; Table S3: Fresh weight (FW) and dry weight (DW) yields (kg) of cones and leaves from four Humulus lupulus varieties across four harvests; Table S4: Essential oil yield (%) from cones and leaves of four Humulus lupulus varieties across four cultivations; Table S5: Complete Pearson correlation analysis of compounds and chemical classes identified in hop cones and leaves; Figure S1: Chromatograms obtained by gas chromatography coupled with mass spectrometry (GC–MS) of the essential oil extracted from the cones of the Cascade variety of Humulus lupulus over the four cultivation cycles 1C: 1st Cultivation (November 2022 to January 2023); 2C: 2nd Cultivation (February–April 2023); 3C: 3rd Cultivation: (September–December 2023) and 4C: 4th Cultivation (January–May 2024); Figure S2: Chromatograms obtained by gas chromatography coupled with mass spectrometry (GC–MS) of the essential oil extracted from the cones of the Columbus variety of Humulus lupulus over the four cultivation cycles. 1C: 1st Cultivation (November 2022 to January 2023); 2C: 2nd Cultivation (February–April 2023); 3C: 3rd Cultivation: (September–December 2023) and 4C: 4th Cultivation (January–May 2024); Figure S3: Chromatograms obtained by gas chromatography coupled with mass spectrometry (GC–MS) of the essential oil extracted from the cones of the Comet variety of Humulus lupulus over the four cultivation cycles. 1C: 1st Cultivation (November 2022 to January 2023); 2C: 2nd Cultivation (February–April 2023); 3C: 3rd Cultivation: (September–December 2023) and 4C: Absent; Figure S4: Chromatograms obtained by gas chromatography coupled with mass spectrometry (GC–MS) of the essential oil extracted from the cones of the Nugget variety of Humulus lupulus over the four cultivation cycles. 1C: 1st Cultivation (November 2022 to January 2023); 2C: 2nd Cultivation (February–April 2023); 3C: 3rd Cultivation: (September–December 2023) and 4C: 4th Cultivation (January–May 2024); Figure S5: Chromatograms obtained by gas chromatography coupled with mass spectrometry (GC–MS) of the essential oil extracted from the leaves of the Cascade variety of Humulus lupulus over the four cultivation cycles. 1C: 1st Cultivation (November 2022 to January 2023); 2C: 2nd Cultivation (February–April 2023); 3C: 3rd Cultivation: (September–December 2023) and 4C: 4th Cultivation (January–May 2024); Figure S6: Chromatograms obtained by gas chromatography coupled with mass spectrometry (GC–MS) of the essential oil extracted from the leaves of the Columbus variety of Humulus lupulus over the four cultivation cycles. 1C: 1st Cultivation (November 2022 to January 2023); 2C: 2nd Cultivation (February–April 2023); 3C: 3rd Cultivation: (September–December 2023) and 4C: 4th Cultivation (January–May 2024); Figure S7: Chromatograms obtained by gas chromatography coupled with mass spectrometry (GC–MS) of the essential oil extracted from the leaves of the Comet variety of Humulus lupulus over the four cultivation cycles. 1C: 1st Cultivation (November 2022 to January 2023); 2C: 2nd Cultivation (February–April 2023); 3C: 3rd Cultivation: (September–December 2023) and 4C: 4th Cultivation (January–May 2024); Figure S8: Chromatograms obtained by gas chromatography coupled with mass spectrometry (GC–MS) of the essential oil extracted from the leaves of the Nugget variety of Humulus lupulus over the four cultivation cycles. 1C: 1st Cultivation (November 2022 to January 2023); 2C: 2nd Cultivation (February–April 2023); 3C: 3rd Cultivation: (September–December 2023) and 4C: 4th Cultivation (January–May 2024).
