# Supplementary material for: Analysis of the Chemical Profile of the Essential Oil from Leaves and Cones of Four Varieties of Humulus lupulus Cultivated in a Subtropical Climate Under Edaphoclimatic Conditions
Source: Molecules. 2026 Oct 10;31(20):3605. doi: 10.3390/molecules31203605 (PMC13707769; doi:10.3390/molecules31203605)
Supplement: Supplementary file 1 [file molecules-31-03605-s001.zip › molecules-4528094-supplementary.pdf]

**Table S1**: Soil chemical properties measured at two soil depths before and during the hop cultivation period in 2022, 2023, and 2024.

| Year | Depth (cm) | pH $CaCl_2$ | P mg $dm^{-3}$ | O.M. % | Ca | K | Mg | Al | CTC | V % |
|---|---|---|---|---|---|---|---|---|---|---|
| | | | | | ................ | | cmol$_c$ $dm^{-3}$ | ................ | | |
| 2022 | 0-20 | 6,01 | 20,57 | 1,68 | 3,12 | 0,23 | 0,56 | 0,00 | 6,79 | 57,88 |
| | 20-40 | 5,92 | 14,62 | 1,56 | 3,85 | 0,43 | 0,20 | 0,00 | 7,57 | 59,31 |
| 2023 | 0-20 | 5,16 | 10,96 | 1,30 | 2,66 | 0,03 | 0,21 | 0,00 | 6,50 | 44,61 |
| | 20-40 | 4,68 | 5,15 | 1,22 | 1,82 | 0,02 | 0,14 | 0,00 | 5,53 | 35,80 |
| 2024 | 0-20 | 5,40 | 14,80 | 1,94 | 2,74 | 0,18 | 0,23 | 0,00 | 5,75 | 54,78 |
| | 20-40 | 4,70 | 4,13 | 1,35 | 1,83 | 0,14 | 0,21 | 0,00 | 4,80 | 45,42 |

*P – Phosphorus; O.M. – Organic Matter; Ca - Calcium; K – Potassium; Mg – Magnesium; Al – Aluminum; CEC – Cation Exchange Capacity; V% - base saturation

**Table S2**: Monthly mean temperature recorded at the experimental site at 09:00, 15:00, and 21:00 from November 2022 to May 2024.

| | Temperature | | |
|---|---|---|---|
| **Period** | **09:00** | **15:00** | **21:00** |
| 11/2022 | 21.42±3.31 $^{ABCa}$ | 27.71±3.94 $^{Aa}$ | 22.81±3.69 $^{Aa}$ |
| 12/2022 | 24.50±2.37 $^{ABCa}$ | 29.64±3.29 $^{Aa}$ | 25.02±2.13 $^{Aa}$ |
| 01/2023 | 23.70±2.02 $^{ABCa}$ | 28.77±3.25 $^{Aa}$ | 24.22±2.54 $^{Aa}$ |
| 02/2023 | 22.23±2.15 $^{ABCa}$ | 27.65± 3.14 $^{Aa}$ | 23.01±2.76 $^{Aa}$ |
| 03/2023 | 23.76±1.72 $^{ABCb}$ | 30.32±2.40 $^{Aa}$ | 24.25±2.51 $^{Ab}$ |
| 04/2023 | 20.92±2.48 $^{ABCa}$ | 27.05±3.35 $^{Aa}$ | 22.27±2.84 $^{Aa}$ |
| 05/2023 | 19.18±2.51 $^{ABCb}$ | 25.96±2.60 $^{Aa}$ | 20.98±2.52 $^{Aab}$ |
| 06/2023 | 16.32±3.48 $^{Ca}$ | 23.01±6.36 $^{Aa}$ | 18.61±4.41 $^{Aa}$ |
| 07/2023 | 17.29±2.90 $^{BCa}$ | 24.64±4.15 $^{Aa}$ | 19.66±3.40 $^{Aa}$ |
| 08/2023 | 19.82±3.19 $^{ABCa}$ | 27.18±4.08 $^{Aa}$ | 21.73±3.37 $^{Aa}$ |
| 09/2023 | 23.35±4.39 $^{ABCa}$ | 29.79±5.54 $^{Aa}$ | 23.51±4.79 $^{Aa}$ |
| 10/2023 | 24.75±2.91 $^{ABCa}$ | 29.63±4.53 $^{Aa}$ | 24.13±3.15 $^{Aa}$ |
| 11/2023 | 24.86±2.89 $^{ABCa}$ | 29.67±4.60 $^{Aa}$ | 24.24±3.13 $^{Aa}$ |
| 12/2023 | 26.77±2.75 $^{Aa}$ | 31.15±3.94 $^{Aa}$ | 25.33±2.48 $^{Aa}$ |
| 01/2024 | 26.67±2.77 $^{ABa}$ | 31.12±4.01 $^{Aa}$ | 25.34±2.41 $^{Aa}$ |
| 02/2024 | 26.08±2.67 $^{ABa}$ | 30.58±3.60 $^{Aa}$ | 24.81±2.67 $^{Aa}$ |
| 03/2024 | 26.22±2.08 $^{ABa}$ | 31.77±3.34 $^{Aa}$ | 25.96±1.86 $^{Aa}$ |
| 04/2024 | 24.33±2.85 $^{ABCa}$ | 31.03±3.22 $^{Aa}$ | 24.38±2.69 $^{Aa}$ |
| 05/2024 | 20.22±5.86 $^{ABCa}$ | 26.89±6.99 $^{Aa}$ | 20.92±5.70 $^{Aa}$ |

Values are expressed as mean ± standard deviation. Different lowercase letters within rows indicate significant differences among times of day, whereas different uppercase letters within columns indicate significant differences among months, according to Tukey's test ($p < 0.05$).

**Table S3:** Fresh weight (FW) and dry weight (DW) yields (kg) of cones and leaves from four Humulus lupulus varieties across four harvests.

| | | Varieties | | | | | | | |
|---|---|---|---|---|---|---|---|---|---|
| | | Cascade | | Columbus | | Comet | | Nugget | |
| **Period** | **Part of the Plant** | **FW** | **DW** | **FW** | **DW** | **FW** | **DW** | **FW** | **DW** |
| **1C** | Cones | 0.17 | 0.05 | 0.90 | 0.22 | 0.12 | 0.04 | 0.11 | 0.04 |
| | Leaves | 1.05 | 0.39 | 3.79 | 1.31 | 0.24 | 0.14 | 1.35 | 0.51 |
| **2C** | Cones | 0.70 | 0.33 | 1.11 | 0.42 | 0.32 | 0.15 | 0.28 | 0.13 |
| | Leaves | 2.45 | 1.29 | 5.21 | 2.51 | 2.60 | 1.28 | 3.43 | 1.92 |
| **3C** | Cones | 3.79 | 0.89 | 4.48 | 1.51 | 1.57 | 0.79 | 0.45 | 0.09 |
| | Leaves | 8.41 | 4.20 | 2.15 | 1.76 | 2.18 | 1.84 | 1.43 | 1.11 |
| **4C** | Cones | 0.79 | 0.16 | 0.67 | 0.17 | ND | ND | 0.50 | 0.25 |
| | Leaves | 2.45 | 0.82 | 1.70 | 0.62 | 0.24 | 0.15 | 1.03 | 0.56 |

**1C**: 1st Cultivation (November 2022 to January 2023); **2C**: 2nd Cultivation (February–April 2023); **3C**: 3rd Cultivation: (September–December 2023) and **4C**: 4th Cultivation (January–May 2024).

**Table S4:** Essential oil yield (%) from cones and leaves of four *Humulus lupulus* varieties across four cultivations.

| **Period** | **Part of the Plant** | **Varieties** | | | |
|---|---|---|---|---|---|
| | | **Cascade** | **Columbus** | **Comet** | **Nugget** |
| 1C | Cones | 0.356±0.072 | 0.054±0.025 | 0.526±0.158 | 1.532±0.399 |
| | Leaves | 0.136±0.139 | 0.054±0.024 | 0.110±0.102 | 0.051±0.001 |
| 2C | Cones | 0.084±0.036 | 0.017±0.005 | 0.695±0.279 | 0.069±0.009 |
| | Leaves | 0.021±0.009 | 0.051±0.020 | 0.106±0.105 | 0.019±0.002 |
| 3C | Cones | 0.070±0.028 | 0.077±0.013 | 0.459±0.058 | 0.510±0.000 |
| | Leaves | 0.067±0.103 | 0.006±0.001 | 0.007±0.004 | 0.348±0.575 |
| 4C | Cones | 0.096±0.047 | 0.064±0.014 | ND | 0.648±0.055 |
| | Leaves | 0.016±0.001 | 0.031±0.006 | 0.110±0.048 | 0.030±0.001 |

Values are expressed as mean ± standard deviation of three technical extraction replicates performed using aliquots of the same composite sample for each cultivar, plant part, and harvest. Technical replicates were not considered independent biological replicates. ND: not Determined. **1C**: 1st Cultivation (November 2022 to January 2023); **2C**: 2nd Cultivation (February–April 2023); **3C**: 3rd Cultivation: (September–December 2023) and **4C**: 4th Cultivation (January–May 2024).

**Table S5: Complete Pearson correlation analysis of compounds and chemical classes identified in hop cones and leaves.**

| Data set | Variable 1 | Variable 2 | Pearson r | p-value | BH-adjusted p-value | * p < 0.05 | * BH-adjusted p < 0.05 | Magnitude | Direction | Significance |
|---|---|---|---|---|---|---|---|---|---|---|
| C-Cmp | Hulupone | Cohumulone | 0.827 | 0.000 | 0.018 | sim | sim | Very strong | Positive | * |
| C-Cmp | γ- muurolene | α-acorenol | 0.812 | 0.000 | 0.018 | sim | sim | Very strong | Positive | * |
| C-Cmp | Carvone | Humulene oxide II | 0.743 | 0.001 | 0.076 | sim | nao | Very strong | Positive | ns |
| C-Cmp | Humulene oxide II | Turmerone | 0.710 | 0.003 | 0.116 | sim | nao | Very strong | Positive | ns |
| C-Cmp | Caryophyllene | Turmerone | -0.688 | 0.005 | 0.140 | sim | nao | Strong | Negative | ns |
| C-Cmp | Carvone | Caryophyllene | -0.624 | 0.013 | 0.330 | sim | nao | Strong | Negative | ns |
| C-Cmp | Humulene | γ-Cadinene | 0.598 | 0.018 | 0.340 | sim | nao | Strong | Positive | ns |
| C-Cmp | β-Pinene | β-Eudesmol | 0.594 | 0.019 | 0.340 | sim | nao | Strong | Positive | ns |
| C-Cmp | Carvone | Cohumulone | 0.592 | 0.020 | 0.340 | sim | nao | Strong | Positive | ns |
| C-Cmp | Turmerone | Cohumulone | 0.569 | 0.027 | 0.392 | sim | nao | Strong | Positive | ns |
| C-Cmp | Caryophyllene | Humulene | 0.564 | 0.028 | 0.392 | sim | nao | Strong | Positive | ns |
| C-Cmp | β-Eudesmene | Turmerone | -0.557 | 0.031 | 0.392 | sim | nao | Strong | Negative | ns |
| C-Cmp | δ-Cadinene | Globulol | 0.551 | 0.033 | 0.392 | sim | nao | Strong | Positive | ns |
| C-Cmp | β-Eudesmene | β-Eudesmol | 0.537 | 0.039 | 0.426 | sim | nao | Strong | Positive | ns |
| C-Cmp | β-Eudesmene | Caryophyllene oxide | 0.520 | 0.047 | 0.426 | sim | nao | Strong | Positive | ns |
| C-Cmp | Turmerone | Hulupone | 0.518 | 0.048 | 0.426 | sim | nao | Strong | Positive | ns |
| C-Cmp | Carvone | Turmerone | 0.516 | 0.049 | 0.426 | sim | nao | Strong | Positive | ns |
| C-Cmp | Caryophyllene | Humulene oxide II | -0.514 | 0.050 | 0.426 | nao | nao | Strong | Negative | ns |
| C-Cmp | α-Selinene | Cohumulone | -0.491 | 0.063 | 0.509 | nao | nao | Moderate | Negative | ns |
| C-Cmp | Humulene | Caryophyllene oxide | -0.481 | 0.070 | 0.516 | nao | nao | Moderate | Negative | ns |
| C-Cmp | γ- muurolene | Cohumulone | -0.477 | 0.072 | 0.516 | nao | nao | Moderate | Negative | ns |
| C-Cmp | γ-Cadinene | Globulol | -0.471 | 0.077 | 0.516 | nao | nao | Moderate | Negative | ns |
| C-Cmp | Caryophyllene | Caryophyllene oxide | -0.463 | 0.083 | 0.516 | nao | nao | Moderate | Negative | ns |
| C-Cmp | Humulene oxide II | Cohumulone | 0.462 | 0.083 | 0.516 | nao | nao | Moderate | Positive | ns |
| C-Cmp | β-Pinene | Humulene | -0.460 | 0.084 | 0.516 | nao | nao | Moderate | Negative | ns |
| C-Cmp | Caryophyllene | Cohumulone | -0.452 | 0.090 | 0.532 | nao | nao | Moderate | Negative | ns |
| C-Cmp | Humulene | β-Eudesmol | -0.448 | 0.094 | 0.532 | nao | nao | Moderate | Negative | ns |
| C-Cmp | β-Pinene | β-Eudesmene | 0.439 | 0.102 | 0.550 | nao | nao | Moderate | Positive | ns |
| C-Cmp | γ-Cadinene | β-Eudesmol | -0.434 | 0.106 | 0.550 | nao | nao | Moderate | Negative | ns |
| C-Cmp | Humulene | Globulol | -0.432 | 0.108 | 0.550 | nao | nao | Moderate | Negative | ns |
| C-Cmp | β-Farnesene | γ- muurolene | 0.421 | 0.118 | 0.581 | nao | nao | Moderate | Positive | ns |
| C-Cmp | Caryophyllene oxide | Humulene oxide II | 0.416 | 0.123 | 0.587 | nao | nao | Moderate | Positive | ns |
| C-Cmp | Carvone | β-Eudesmene | -0.409 | 0.130 | 0.587 | nao | nao | Moderate | Negative | ns |
| C-Cmp | Carvone | Caryophyllene oxide | 0.409 | 0.131 | 0.587 | nao | nao | Moderate | Positive | ns |
| C-Cmp | Caryophyllene oxide | α-acorenol | -0.399 | 0.140 | 0.588 | nao | nao | Moderate | Negative | ns |
| C-Cmp | Carvone | Humulene | -0.398 | 0.142 | 0.588 | nao | nao | Moderate | Negative | ns |
| C-Cmp | α-acorenol | β-Eudesmol | -0.398 | 0.142 | 0.588 | nao | nao | Moderate | Negative | ns |
| C-Cmp | γ- muurolene | α-Selinene | 0.391 | 0.150 | 0.589 | nao | nao | Moderate | Positive | ns |
| C-Cmp | Caryophyllene oxide | β-Eudesmol | 0.390 | 0.151 | 0.589 | nao | nao | Moderate | Positive | ns |
| C-Cmp | γ-Cadinene | Caryophyllene oxide | -0.387 | 0.154 | 0.589 | nao | nao | Moderate | Negative | ns |

| | | | | | | | | | | |
|---|---|---|---|---|---|---|---|---|---|---|
| C-Cmp | Caryophyllene | γ-Cadinene | 0.380 | 0.162 | 0.605 | nao | nao | Moderate | Positive | ns |
| C-Cmp | β-Eudesmene | Cohumulone | -0.374 | 0.170 | 0.605 | nao | nao | Moderate | Negative | ns |
| C-Cmp | β-Pinene | α-Selinene | 0.371 | 0.174 | 0.605 | nao | nao | Moderate | Positive | ns |
| C-Cmp | β-Eudesmene | γ-Cadinene | -0.368 | 0.177 | 0.605 | nao | nao | Moderate | Negative | ns |
| C-Cmp | γ- muurolene | Humulene oxide II | -0.367 | 0.178 | 0.605 | nao | nao | Moderate | Negative | ns |
| C-Cmp | β-Eudesmene | Humulene oxide II | -0.364 | 0.182 | 0.605 | nao | nao | Moderate | Negative | ns |
| C-Cmp | α-acorenol | Cohumulone | -0.359 | 0.189 | 0.615 | nao | nao | Moderate | Negative | ns |
| C-Cmp | γ- muurolene | Turmerone | -0.353 | 0.197 | 0.629 | nao | nao | Moderate | Negative | ns |
| C-Cmp | Caryophyllene | Hulupone | -0.348 | 0.204 | 0.637 | nao | nao | Moderate | Negative | ns |
| C-Cmp | β-Pinene | α-acorenol | -0.344 | 0.210 | 0.641 | nao | nao | Moderate | Negative | ns |
| C-Cmp | γ- muurolene | Hulupone | -0.341 | 0.214 | 0.641 | nao | nao | Moderate | Negative | ns |
| C-Cmp | Humulene | Turmerone | -0.337 | 0.219 | 0.645 | nao | nao | Moderate | Negative | ns |
| C-Cmp | Carvone | δ-Cadinene | -0.334 | 0.224 | 0.645 | nao | nao | Moderate | Negative | ns |
| C-Cmp | β-Pinene | γ-Cadinene | -0.329 | 0.231 | 0.648 | nao | nao | Moderate | Negative | ns |
| C-Cmp | α-Selinene | γ-Cadinene | -0.328 | 0.233 | 0.648 | nao | nao | Moderate | Negative | ns |
| C-Cmp | α-Selinene | δ-Cadinene | 0.324 | 0.239 | 0.648 | nao | nao | Moderate | Positive | ns |
| C-Cmp | γ-Cadinene | δ-Cadinene | -0.322 | 0.241 | 0.648 | nao | nao | Moderate | Negative | ns |
| C-Cmp | γ- muurolene | γ-Cadinene | -0.316 | 0.251 | 0.662 | nao | nao | Moderate | Negative | ns |
| C-Cmp | γ- muurolene | Globulol | 0.311 | 0.259 | 0.663 | nao | nao | Moderate | Positive | ns |
| C-Cmp | β-Farnesene | α-acorenol | 0.310 | 0.260 | 0.663 | nao | nao | Moderate | Positive | ns |
| C-Cmp | α-Selinene | Humulene oxide II | -0.301 | 0.276 | 0.693 | nao | nao | Moderate | Negative | ns |
| C-Cmp | β-Eudesmene | Hulupone | -0.287 | 0.300 | 0.694 | nao | nao | Weak | Negative | ns |
| C-Cmp | β-Pinene | Turmerone | -0.287 | 0.300 | 0.694 | nao | nao | Weak | Negative | ns |
| C-Cmp | Globulol | Cohumulone | -0.285 | 0.303 | 0.694 | nao | nao | Weak | Negative | ns |
| C-Cmp | α-Selinene | Hulupone | -0.284 | 0.305 | 0.694 | nao | nao | Weak | Negative | ns |
| C-Cmp | β-Pinene | Globulol | 0.277 | 0.318 | 0.694 | nao | nao | Weak | Positive | ns |
| C-Cmp | α-Selinene | β-Eudesmol | 0.274 | 0.323 | 0.694 | nao | nao | Weak | Positive | ns |
| C-Cmp | α-acorenol | Hulupone | -0.273 | 0.325 | 0.694 | nao | nao | Weak | Negative | ns |
| C-Cmp | Humulene | β-Farnesene | -0.273 | 0.325 | 0.694 | nao | nao | Weak | Negative | ns |
| C-Cmp | Humulene | Humulene oxide II | -0.272 | 0.326 | 0.694 | nao | nao | Weak | Negative | ns |
| C-Cmp | β-Eudesmene | Globulol | 0.272 | 0.326 | 0.694 | nao | nao | Weak | Positive | ns |
| C-Cmp | Humulene | Cohumulone | -0.272 | 0.327 | 0.694 | nao | nao | Weak | Negative | ns |
| C-Cmp | Carvone | Globulol | -0.270 | 0.331 | 0.694 | nao | nao | Weak | Negative | ns |
| C-Cmp | δ-Cadinene | Cohumulone | -0.265 | 0.341 | 0.695 | nao | nao | Weak | Negative | ns |
| C-Cmp | δ-Cadinene | Turmerone | 0.262 | 0.345 | 0.695 | nao | nao | Weak | Positive | ns |
| C-Cmp | Globulol | β-Eudesmol | 0.262 | 0.345 | 0.695 | nao | nao | Weak | Positive | ns |
| C-Cmp | β-Farnesene | Cohumulone | -0.255 | 0.359 | 0.713 | nao | nao | Weak | Negative | ns |
| C-Cmp | Carvone | β-Eudesmol | -0.251 | 0.367 | 0.716 | nao | nao | Weak | Negative | ns |
| C-Cmp | β-Pinene | Humulene oxide II | -0.250 | 0.370 | 0.716 | nao | nao | Weak | Negative | ns |
| C-Cmp | δ-Cadinene | Caryophyllene oxide | -0.241 | 0.386 | 0.723 | nao | nao | Weak | Negative | ns |
| C-Cmp | β-Farnesene | β-Eudesmene | -0.241 | 0.387 | 0.723 | nao | nao | Weak | Negative | ns |
| C-Cmp | β-Eudesmol | Cohumulone | -0.239 | 0.390 | 0.723 | nao | nao | Weak | Negative | ns |
| C-Cmp | β-Eudesmene | α-acorenol | -0.238 | 0.393 | 0.723 | nao | nao | Weak | Negative | ns |
| C-Cmp | γ- muurolene | δ-Cadinene | 0.236 | 0.397 | 0.723 | nao | nao | Weak | Positive | ns |
| C-Cmp | γ-Cadinene | Humulene oxide II | -0.226 | 0.417 | 0.741 | nao | nao | Weak | Negative | ns |

| | | | | | | | | | | |
|---|---|---|---|---|---|---|---|---|---|---|
| C-Cmp | Humulene | Hulupone | -0.225 | 0.420 | 0.741 | nao | nao | Weak | Negative | ns |
| C-Cmp | α-Selinene | Globulol | 0.222 | 0.426 | 0.741 | nao | nao | Weak | Positive | ns |
| C-Cmp | β-Pinene | Cohumulone | -0.222 | 0.426 | 0.741 | nao | nao | Weak | Negative | ns |
| C-Cmp | Humulene oxide II | α-acorenol | -0.212 | 0.448 | 0.767 | nao | nao | Weak | Negative | ns |
| C-Cmp | Carvone | γ-Cadinene | -0.210 | 0.452 | 0.767 | nao | nao | Weak | Negative | ns |
| C-Cmp | α-acorenol | Turmerone | -0.208 | 0.456 | 0.767 | nao | nao | Weak | Negative | ns |
| C-Cmp | β-Pinene | δ-Cadinene | -0.203 | 0.467 | 0.771 | nao | nao | Weak | Negative | ns |
| C-Cmp | β-Pinene | Caryophyllene | 0.203 | 0.469 | 0.771 | nao | nao | Weak | Positive | ns |
| C-Cmp | β-Farnesene | Caryophyllene oxide | -0.198 | 0.479 | 0.779 | nao | nao | Weak | Negative | ns |
| C-Cmp | β-Eudesmol | Turmerone | -0.190 | 0.497 | 0.799 | nao | nao | Weak | Negative | ns |
| C-Cmp | γ- muurolene | Caryophyllene oxide | -0.188 | 0.501 | 0.799 | nao | nao | Weak | Negative | ns |
| C-Cmp | α-Selinene | Caryophyllene oxide | -0.184 | 0.512 | 0.808 | nao | nao | Weak | Negative | ns |
| C-Cmp | β-Pinene | Hulupone | -0.173 | 0.537 | 0.827 | nao | nao | Weak | Negative | ns |
| C-Cmp | Caryophyllene oxide | Cohumulone | 0.171 | 0.543 | 0.827 | nao | nao | Weak | Positive | ns |
| C-Cmp | β-Farnesene | Hulupone | -0.170 | 0.544 | 0.827 | nao | nao | Weak | Negative | ns |
| C-Cmp | Carvone | α-Selinene | -0.170 | 0.546 | 0.827 | nao | nao | Weak | Negative | ns |
| C-Cmp | Caryophyllene | β-Eudesmene | 0.160 | 0.570 | 0.841 | nao | nao | Weak | Positive | ns |
| C-Cmp | Carvone | Hulupone | 0.158 | 0.575 | 0.841 | nao | nao | Weak | Positive | ns |
| C-Cmp | β-Eudesmol | Hulupone | -0.154 | 0.583 | 0.841 | nao | nao | Weak | Negative | ns |
| C-Cmp | β-Pinene | β-Farnesene | 0.151 | 0.590 | 0.841 | nao | nao | Weak | Positive | ns |
| C-Cmp | Globulol | Hulupone | -0.147 | 0.600 | 0.841 | nao | nao | Weak | Negative | ns |
| C-Cmp | β-Pinene | Carvone | -0.147 | 0.601 | 0.841 | nao | nao | Weak | Negative | ns |
| C-Cmp | Humulene | β-Eudesmene | -0.147 | 0.602 | 0.841 | nao | nao | Weak | Negative | ns |
| C-Cmp | Caryophyllene | α-Selinene | -0.146 | 0.604 | 0.841 | nao | nao | Weak | Negative | ns |
| C-Cmp | Humulene | α-acorenol | 0.139 | 0.622 | 0.841 | nao | nao | Weak | Positive | ns |
| C-Cmp | Carvone | β-Farnesene | -0.139 | 0.623 | 0.841 | nao | nao | Weak | Negative | ns |
| C-Cmp | Humulene oxide II | β-Eudesmol | -0.138 | 0.623 | 0.841 | nao | nao | Weak | Negative | ns |
| C-Cmp | β-Farnesene | β-Eudesmol | 0.136 | 0.630 | 0.841 | nao | nao | Weak | Positive | ns |
| C-Cmp | β-Farnesene | Humulene oxide II | -0.135 | 0.632 | 0.841 | nao | nao | Weak | Negative | ns |
| C-Cmp | Carvone | γ- muurolene | -0.135 | 0.632 | 0.841 | nao | nao | Weak | Negative | ns |
| C-Cmp | Caryophyllene oxide | Globulol | 0.130 | 0.644 | 0.848 | nao | nao | Weak | Positive | ns |
| C-Cmp | δ-Cadinene | Humulene oxide II | 0.128 | 0.649 | 0.848 | nao | nao | Weak | Positive | ns |
| C-Cmp | Humulene | δ-Cadinene | 0.126 | 0.654 | 0.848 | nao | nao | Weak | Positive | ns |
| C-Cmp | β-Eudesmene | α-Selinene | 0.119 | 0.673 | 0.865 | nao | nao | Weak | Positive | ns |
| C-Cmp | β-Farnesene | Globulol | 0.114 | 0.686 | 0.872 | nao | nao | Weak | Positive | ns |
| C-Cmp | β-Eudesmene | δ-Cadinene | -0.113 | 0.689 | 0.872 | nao | nao | Weak | Negative | ns |
| C-Cmp | α-Selinene | α-acorenol | 0.109 | 0.699 | 0.876 | nao | nao | Weak | Positive | ns |
| C-Cmp | Humulene | γ- muurolene | -0.107 | 0.704 | 0.876 | nao | nao | Weak | Negative | ns |
| C-Cmp | Caryophyllene oxide | Hulupone | -0.104 | 0.713 | 0.880 | nao | nao | Weak | Negative | ns |
| C-Cmp | δ-Cadinene | α-acorenol | 0.094 | 0.739 | 0.905 | nao | nao | Weak | Positive | ns |
| C-Cmp | γ-Cadinene | Hulupone | 0.090 | 0.751 | 0.908 | nao | nao | Weak | Positive | ns |
| C-Cmp | Caryophyllene oxide | Turmerone | 0.087 | 0.758 | 0.908 | nao | nao | Weak | Positive | ns |
| C-Cmp | δ-Cadinene | Hulupone | -0.086 | 0.759 | 0.908 | nao | nao | Weak | Negative | ns |
| C-Cmp | α-Selinene | Turmerone | -0.084 | 0.767 | 0.909 | nao | nao | Weak | Negative | ns |

| | | | | | | | | | | |
|---|---|---|---|---|---|---|---|---|---|---|
| C-Cmp | Caryophyllene | β-Farnesene | -0.082 | 0.772 | 0.909 | nao | nao | Weak | Negative | ns |
| C-Cmp | β-Farnesene | γ-Cadinene | 0.078 | 0.783 | 0.909 | nao | nao | Weak | Positive | ns |
| C-Cmp | β-Pinene | γ- muurolene | -0.077 | 0.786 | 0.909 | nao | nao | Weak | Negative | ns |
| C-Cmp | β-Farnesene | α-Selinene | 0.075 | 0.791 | 0.909 | nao | nao | Weak | Positive | ns |
| C-Cmp | Humulene | α-Selinene | -0.071 | 0.800 | 0.912 | nao | nao | Weak | Negative | ns |
| C-Cmp | Caryophyllene | α-acorenol | 0.070 | 0.805 | 0.912 | nao | nao | Weak | Positive | ns |
| C-Cmp | Globulol | α-acorenol | -0.061 | 0.830 | 0.921 | nao | nao | Weak | Negative | ns |
| C-Cmp | β-Pinene | Caryophyllene oxide | 0.059 | 0.834 | 0.921 | nao | nao | Weak | Positive | ns |
| C-Cmp | Globulol | Humulene oxide II | -0.059 | 0.835 | 0.921 | nao | nao | Weak | Negative | ns |
| C-Cmp | γ- muurolene | β-Eudesmol | -0.058 | 0.837 | 0.921 | nao | nao | Weak | Negative | ns |
| C-Cmp | Humulene oxide II | Hulupone | 0.043 | 0.879 | 0.960 | nao | nao | Weak | Positive | ns |
| C-Cmp | Carvone | α-acorenol | -0.041 | 0.884 | 0.960 | nao | nao | Weak | Negative | ns |
| C-Cmp | Caryophyllene | Globulol | 0.031 | 0.912 | 0.974 | nao | nao | Weak | Positive | ns |
| C-Cmp | Caryophyllene | β-Eudesmol | 0.031 | 0.913 | 0.974 | nao | nao | Weak | Positive | ns |
| C-Cmp | β-Farnesene | δ-Cadinene | 0.025 | 0.931 | 0.974 | nao | nao | Weak | Positive | ns |
| C-Cmp | Globulol | Turmerone | -0.023 | 0.935 | 0.974 | nao | nao | Weak | Negative | ns |
| C-Cmp | δ-Cadinene | β-Eudesmol | 0.022 | 0.939 | 0.974 | nao | nao | Weak | Positive | ns |
| C-Cmp | γ-Cadinene | Turmerone | -0.022 | 0.939 | 0.974 | nao | nao | Weak | Negative | ns |
| C-Cmp | γ-Cadinene | α-acorenol | -0.020 | 0.942 | 0.974 | nao | nao | Weak | Negative | ns |
| C-Cmp | Caryophyllene | γ- muurolene | 0.012 | 0.967 | 0.987 | nao | nao | Weak | Positive | ns |
| C-Cmp | γ-Cadinene | Cohumulone | -0.011 | 0.969 | 0.987 | nao | nao | Weak | Negative | ns |
| C-Cmp | Caryophyllene | δ-Cadinene | -0.007 | 0.979 | 0.987 | nao | nao | Weak | Negative | ns |
| C-Cmp | β-Farnesene | Turmerone | 0.007 | 0.980 | 0.987 | nao | nao | Weak | Positive | ns |
| C-Cmp | γ- muurolene | β-Eudesmene | 0.003 | 0.991 | 0.991 | nao | nao | Weak | Positive | ns |
| C-Class | Alpha and Beta Acids | ns | 0.932 | 0.000 | 0.000 | sim | sim | Very strong | Positive | * |
| C-Class | Hydrocarbon Sesquiterpenes | Oxygenated Sesquiterpenes | -0.767 | 0.001 | 0.015 | sim | sim | Very strong | Negative | * |
| C-Class | Ketones | Alpha and Beta Acids | 0.728 | 0.002 | 0.025 | sim | sim | Very strong | Positive | * |
| C-Class | Hydrocarbon Sesquiterpenes | Ketones | -0.665 | 0.007 | 0.062 | sim | nao | Strong | Negative | ns |
| C-Class | Ketones | ns | 0.574 | 0.025 | 0.182 | sim | nao | Strong | Positive | ns |
| C-Class | Hydrocarbon Sesquiterpenes | Alpha and Beta Acids | -0.552 | 0.033 | 0.185 | sim | nao | Strong | Negative | ns |
| C-Class | Oxygenated Monoterpenes | Hydrocarbon Sesquiterpenes | -0.537 | 0.039 | 0.185 | sim | nao | Strong | Negative | ns |
| C-Class | Hydrocarbon Sesquiterpenes | ns | -0.532 | 0.041 | 0.185 | sim | nao | Strong | Negative | ns |
| C-Class | Oxygenated Monoterpenes | Oxygenated Sesquiterpenes | 0.507 | 0.054 | 0.216 | nao | nao | Strong | Positive | ns |
| C-Class | Oxygenated Sesquiterpenes | Ketones | 0.427 | 0.112 | 0.404 | nao | nao | Moderate | Positive | ns |
| C-Class | Oxygenated Sesquiterpenes | Diterpenes | 0.415 | 0.124 | 0.407 | nao | nao | Moderate | Positive | ns |
| C-Class | Diterpenes | ns | -0.372 | 0.172 | 0.516 | nao | nao | Moderate | Negative | ns |
| C-Class | Terpenoid Ester | Alpha and Beta Acids | -0.310 | 0.261 | 0.698 | nao | nao | Moderate | Negative | ns |
| C-Class | Terpenoid Ester | ns | -0.303 | 0.272 | 0.698 | nao | nao | Moderate | Negative | ns |
| C-Class | Hydrocarbon Monoterpenes | Hydrocarbon Sesquiterpenes | -0.256 | 0.356 | 0.737 | nao | nao | Weak | Negative | ns |
| C-Class | Ketones | Terpenoid Ester | -0.250 | 0.368 | 0.737 | nao | nao | Weak | Negative | ns |
| C-Class | Oxygenated Sesquiterpenes | Terpenoid Ester | -0.247 | 0.374 | 0.737 | nao | nao | Weak | Negative | ns |
| C-Class | Hydrocarbon Sesquiterpenes | Terpenoid Ester | 0.245 | 0.380 | 0.737 | nao | nao | Weak | Positive | ns |
| C-Class | Diterpenes | Terpenoid Ester | 0.240 | 0.389 | 0.737 | nao | nao | Weak | Positive | ns |

| | | | | | | | | | | |
|---|---|---|---|---|---|---|---|---|---|---|
| C-Class | Oxygenated Sesquiterpenes | Alpha and Beta Acids | 0.184 | 0.510 | 0.882 | nao | nao | Weak | Positive | ns |
| C-Class | Diterpenes | Alpha and Beta Acids | -0.178 | 0.527 | 0.882 | nao | nao | Weak | Negative | ns |
| C-Class | Hydrocarbon Monoterpenes | Oxygenated Sesquiterpenes | -0.168 | 0.548 | 0.882 | nao | nao | Weak | Negative | ns |
| C-Class | Hydrocarbon Monoterpenes | Diterpenes | -0.150 | 0.594 | 0.882 | nao | nao | Weak | Negative | ns |
| C-Class | Oxygenated Monoterpenes | Diterpenes | 0.139 | 0.622 | 0.882 | nao | nao | Weak | Positive | ns |
| C-Class | Hydrocarbon Monoterpenes | Alpha and Beta Acids | -0.136 | 0.630 | 0.882 | nao | nao | Weak | Negative | ns |
| C-Class | Hydrocarbon Sesquiterpenes | Diterpenes | -0.133 | 0.637 | 0.882 | nao | nao | Weak | Negative | ns |
| C-Class | Oxygenated Sesquiterpenes | ns | 0.101 | 0.720 | 0.939 | nao | nao | Weak | Positive | ns |
| C-Class | Hydrocarbon Monoterpenes | ns | -0.091 | 0.748 | 0.939 | nao | nao | Weak | Negative | ns |
| C-Class | Oxygenated Monoterpenes | Terpenoid Ester | 0.088 | 0.756 | 0.939 | nao | nao | Weak | Positive | ns |
| C-Class | Oxygenated Monoterpenes | ns | 0.072 | 0.799 | 0.942 | nao | nao | Weak | Positive | ns |
| C-Class | Hydrocarbon Monoterpenes | Ketones | 0.068 | 0.811 | 0.942 | nao | nao | Weak | Positive | ns |
| C-Class | Hydrocarbon Monoterpenes | Oxygenated Monoterpenes | 0.055 | 0.845 | 0.942 | nao | nao | Weak | Positive | ns |
| C-Class | Diterpenes | Ketones | 0.049 | 0.863 | 0.942 | nao | nao | Weak | Positive | ns |
| C-Class | Oxygenated Monoterpenes | Alpha and Beta Acids | -0.030 | 0.915 | 0.956 | nao | nao | Weak | Negative | ns |
| C-Class | Oxygenated Monoterpenes | Ketones | 0.025 | 0.929 | 0.956 | nao | nao | Weak | Positive | ns |
| C-Class | Hydrocarbon Monoterpenes | Terpenoid Ester | 0.009 | 0.975 | 0.975 | nao | nao | Weak | Positive | ns |
| L-Cmp | β-Eudesmene | α-Selinene | 0.872 | 0.000 | 0.001 | sim | sim | Very strong | Positive | * |
| L-Cmp | γ-Muurolene | γ-Cadinene | 0.858 | 0.000 | 0.001 | sim | sim | Very strong | Positive | * |
| L-Cmp | Humulene | β-Eudesmene | -0.658 | 0.006 | 0.146 | sim | nao | Strong | Negative | ns |
| L-Cmp | Humulene | α-Selinene | -0.609 | 0.012 | 0.239 | sim | nao | Strong | Negative | ns |
| L-Cmp | Cariophyllene | Humulene oxide II | -0.558 | 0.025 | 0.347 | sim | nao | Strong | Negative | ns |
| L-Cmp | Cariophyllene | Phytol | -0.544 | 0.029 | 0.347 | sim | nao | Strong | Negative | ns |
| L-Cmp | β-Eudesmene | β-Eudesmol | 0.539 | 0.031 | 0.347 | sim | nao | Strong | Positive | ns |
| L-Cmp | β-Pinene | Humulene | 0.525 | 0.037 | 0.355 | sim | nao | Strong | Positive | ns |
| L-Cmp | Cariophyllene | α-Selinene | 0.509 | 0.044 | 0.355 | sim | nao | Strong | Positive | ns |
| L-Cmp | Cariophyllene | 2-pentadecanone | -0.506 | 0.046 | 0.355 | sim | nao | Strong | Negative | ns |
| L-Cmp | Cariophyllene | Cariophyllene oxide | -0.470 | 0.066 | 0.441 | nao | nao | Moderate | Negative | ns |
| L-Cmp | Humulene | β-Eudesmol | -0.468 | 0.068 | 0.441 | nao | nao | Moderate | Negative | ns |
| L-Cmp | Cariophyllene | β-Eudesmene | 0.449 | 0.081 | 0.486 | nao | nao | Moderate | Positive | ns |
| L-Cmp | α-Selinene | β-Eudesmol | 0.432 | 0.095 | 0.487 | nao | nao | Moderate | Positive | ns |
| L-Cmp | β-Pinene | Cariophyllene | 0.431 | 0.096 | 0.487 | nao | nao | Moderate | Positive | ns |
| L-Cmp | β-Eudesmene | β-Eudesmol | 0.410 | 0.115 | 0.487 | nao | nao | Moderate | Positive | ns |
| L-Cmp | Humulene | β-Eudesmol | -0.407 | 0.118 | 0.487 | nao | nao | Moderate | Negative | ns |
| L-Cmp | γ-Muurolene | α-Selinene | -0.405 | 0.119 | 0.487 | nao | nao | Moderate | Negative | ns |
| L-Cmp | Humulene | Cariophyllene oxide | -0.398 | 0.127 | 0.487 | nao | nao | Moderate | Negative | ns |
| L-Cmp | β-Pinene | Phytol | -0.394 | 0.131 | 0.487 | nao | nao | Moderate | Negative | ns |
| L-Cmp | 2-pentadecanone | Phytol | 0.385 | 0.141 | 0.487 | nao | nao | Moderate | Positive | ns |
| L-Cmp | γ-Muurolene | β-Eudesmol | -0.385 | 0.141 | 0.487 | nao | nao | Moderate | Negative | ns |
| L-Cmp | β-Pinene | γ-Cadinene | -0.383 | 0.144 | 0.487 | nao | nao | Moderate | Negative | ns |
| L-Cmp | γ-Muurolene | β-Eudesmene | -0.362 | 0.169 | 0.512 | nao | nao | Moderate | Negative | ns |
| L-Cmp | α-Selinene | β-Eudesmol | 0.358 | 0.174 | 0.512 | nao | nao | Moderate | Positive | ns |

| | | | | | | | | | | |
|---|---|---|---|---|---|---|---|---|---|---|
| L-Cmp | β-Pinene | Humulene oxide II | -0.352 | 0.181 | 0.512 | nao | nao | Moderate | Negative | ns |
| L-Cmp | β-Pinene | 2-pentadecanone | -0.348 | 0.186 | 0.512 | nao | nao | Moderate | Negative | ns |
| L-Cmp | α-Selinene | Phytol | -0.347 | 0.188 | 0.512 | nao | nao | Moderate | Negative | ns |
| L-Cmp | Humulene oxide II | 2-pentadecanone | 0.345 | 0.190 | 0.512 | nao | nao | Moderate | Positive | ns |
| L-Cmp | Cariophyllene oxide | Humulene oxide II | 0.333 | 0.208 | 0.540 | nao | nao | Moderate | Positive | ns |
| L-Cmp | β-Pinene | Cariophyllene oxide | -0.279 | 0.295 | 0.719 | nao | nao | Weak | Negative | ns |
| L-Cmp | Cariophyllene | γ-Muurolene | -0.270 | 0.312 | 0.719 | nao | nao | Weak | Negative | ns |
| L-Cmp | γ-Cadinene | Humulene oxide II | -0.262 | 0.328 | 0.719 | nao | nao | Weak | Negative | ns |
| L-Cmp | Cariophyllene | β-Eudesmol | 0.258 | 0.334 | 0.719 | nao | nao | Weak | Positive | ns |
| L-Cmp | β-Eudesmol | Phytol | -0.257 | 0.337 | 0.719 | nao | nao | Weak | Negative | ns |
| L-Cmp | Cariophyllene oxide | Phytol | -0.256 | 0.339 | 0.719 | nao | nao | Weak | Negative | ns |
| L-Cmp | β-Eudesmene | Cariophyllene oxide | -0.255 | 0.341 | 0.719 | nao | nao | Weak | Negative | ns |
| L-Cmp | Cariophyllene | β-Eudesmol | 0.229 | 0.393 | 0.790 | nao | nao | Weak | Positive | ns |
| L-Cmp | β-Pinene | γ-Muurolene | -0.228 | 0.395 | 0.790 | nao | nao | Weak | Negative | ns |
| L-Cmp | α-Selinene | γ-Cadinene | -0.215 | 0.423 | 0.798 | nao | nao | Weak | Negative | ns |
| L-Cmp | γ-Cadinene | 2-pentadecanone | -0.208 | 0.439 | 0.798 | nao | nao | Weak | Negative | ns |
| L-Cmp | α-Selinene | Cariophyllene oxide | -0.202 | 0.454 | 0.798 | nao | nao | Weak | Negative | ns |
| L-Cmp | β-Eudesmol | 2-pentadecanone | 0.199 | 0.459 | 0.798 | nao | nao | Weak | Positive | ns |
| L-Cmp | β-Eudesmene | Phytol | -0.199 | 0.460 | 0.798 | nao | nao | Weak | Negative | ns |
| L-Cmp | β-Pinene | β-Eudesmol | -0.199 | 0.461 | 0.798 | nao | nao | Weak | Negative | ns |
| L-Cmp | Humulene | γ-Muurolene | 0.187 | 0.489 | 0.829 | nao | nao | Weak | Positive | ns |
| L-Cmp | Humulene oxide II | β-Eudesmol | 0.169 | 0.532 | 0.850 | nao | nao | Weak | Positive | ns |
| L-Cmp | α-Selinene | 2-pentadecanone | -0.168 | 0.534 | 0.850 | nao | nao | Weak | Negative | ns |
| L-Cmp | β-Pinene | β-Eudesmene | -0.168 | 0.534 | 0.850 | nao | nao | Weak | Negative | ns |
| L-Cmp | α-Selinene | Humulene oxide II | -0.139 | 0.608 | 0.947 | nao | nao | Weak | Negative | ns |
| L-Cmp | γ-Cadinene | β-Eudesmol | -0.133 | 0.623 | 0.947 | nao | nao | Weak | Negative | ns |
| L-Cmp | β-Eudesmene | 2-pentadecanone | -0.127 | 0.639 | 0.947 | nao | nao | Weak | Negative | ns |
| L-Cmp | Humulene | Humulene oxide II | -0.125 | 0.643 | 0.947 | nao | nao | Weak | Negative | ns |
| L-Cmp | β-Eudesmene | γ-Cadinene | -0.110 | 0.685 | 0.957 | nao | nao | Weak | Negative | ns |
| L-Cmp | γ-Muurolene | Cariophyllene oxide | 0.109 | 0.689 | 0.957 | nao | nao | Weak | Positive | ns |
| L-Cmp | β-Eudesmol | Phytol | -0.108 | 0.690 | 0.957 | nao | nao | Weak | Negative | ns |
| L-Cmp | β-Pinene | β-Eudesmol | -0.104 | 0.702 | 0.957 | nao | nao | Weak | Negative | ns |
| L-Cmp | Humulene | 2-pentadecanone | -0.100 | 0.712 | 0.957 | nao | nao | Weak | Negative | ns |
| L-Cmp | γ-Cadinene | β-Eudesmol | -0.093 | 0.733 | 0.960 | nao | nao | Weak | Negative | ns |
| L-Cmp | γ-Cadinene | Phytol | 0.088 | 0.746 | 0.960 | nao | nao | Weak | Positive | ns |
| L-Cmp | Humulene oxide II | Phytol | 0.076 | 0.779 | 0.960 | nao | nao | Weak | Positive | ns |
| L-Cmp | β-Pinene | α-Selinene | 0.075 | 0.784 | 0.960 | nao | nao | Weak | Positive | ns |
| L-Cmp | β-Eudesmol | β-Eudesmol | 0.063 | 0.817 | 0.960 | nao | nao | Weak | Positive | ns |
| L-Cmp | Cariophyllene | Humulene | 0.058 | 0.830 | 0.960 | nao | nao | Weak | Positive | ns |
| L-Cmp | Humulene oxide II | β-Eudesmol | -0.057 | 0.833 | 0.960 | nao | nao | Weak | Negative | ns |
| L-Cmp | Cariophyllene | γ-Cadinene | -0.057 | 0.833 | 0.960 | nao | nao | Weak | Negative | ns |
| L-Cmp | γ-Muurolene | 2-pentadecanone | -0.055 | 0.841 | 0.960 | nao | nao | Weak | Negative | ns |
| L-Cmp | Humulene | Phytol | 0.049 | 0.858 | 0.960 | nao | nao | Weak | Positive | ns |

| | | | | | | | | | | |
|---|---|---|---|---|---|---|---|---|---|---|
| L-Cmp | β-Eudesmene | Humulene oxide II | -0.048 | 0.860 | 0.960 | nao | nao | Weak | Negative | ns |
| L-Cmp | γ-Muurolene | β-Eudesmol | 0.047 | 0.861 | 0.960 | nao | nao | Weak | Positive | ns |
| L-Cmp | Cariophyllene oxide | β-Eudesmol | -0.041 | 0.880 | 0.967 | nao | nao | Weak | Negative | ns |
| L-Cmp | Cariophyllene oxide | β-Eudesmol | -0.034 | 0.902 | 0.977 | nao | nao | Weak | Negative | ns |
| L-Cmp | γ-Cadinene | Cariophyllene oxide | -0.023 | 0.934 | 0.980 | nao | nao | Weak | Negative | ns |
| L-Cmp | γ-Muurolene | Phytol | 0.017 | 0.950 | 0.980 | nao | nao | Weak | Positive | ns |
| L-Cmp | β-Eudesmol | 2-pentadecanone | 0.013 | 0.961 | 0.980 | nao | nao | Weak | Positive | ns |
| L-Cmp | Cariophyllene oxide | 2-pentadecanone | 0.012 | 0.965 | 0.980 | nao | nao | Weak | Positive | ns |
| L-Cmp | γ-Muurolene | Humulene oxide II | -0.011 | 0.967 | 0.980 | nao | nao | Weak | Negative | ns |
| L-Cmp | Humulene | γ-Cadinene | 0.000 | 0.999 | 0.999 | nao | nao | Weak | Positive | ns |
| L-Class | Hydrocarbon Sesquiterpenes | Ketones | -0.892 | 0.000 | 0.000 | sim | sim | Very strong | Negative | * |
| L-Class | Hydrocarbon Sesquiterpenes | Oxygenated Sesquiterpenes | -0.559 | 0.024 | 0.073 | sim | nao | Strong | Negative | ns |
| L-Class | Oxygenated Sesquiterpenes | Ketones | 0.514 | 0.042 | 0.083 | sim | nao | Strong | Positive | ns |
| L-Class | Hydrocarbon Sesquiterpenes | Diterpenes | -0.477 | 0.062 | 0.083 | nao | nao | Moderate | Negative | ns |
| L-Class | Diterpenes | Ketones | 0.466 | 0.069 | 0.083 | nao | nao | Moderate | Positive | ns |
| L-Class | Oxygenated Sesquiterpenes | Diterpenes | -0.296 | 0.265 | 0.265 | nao | nao | Weak | Negative | ns |

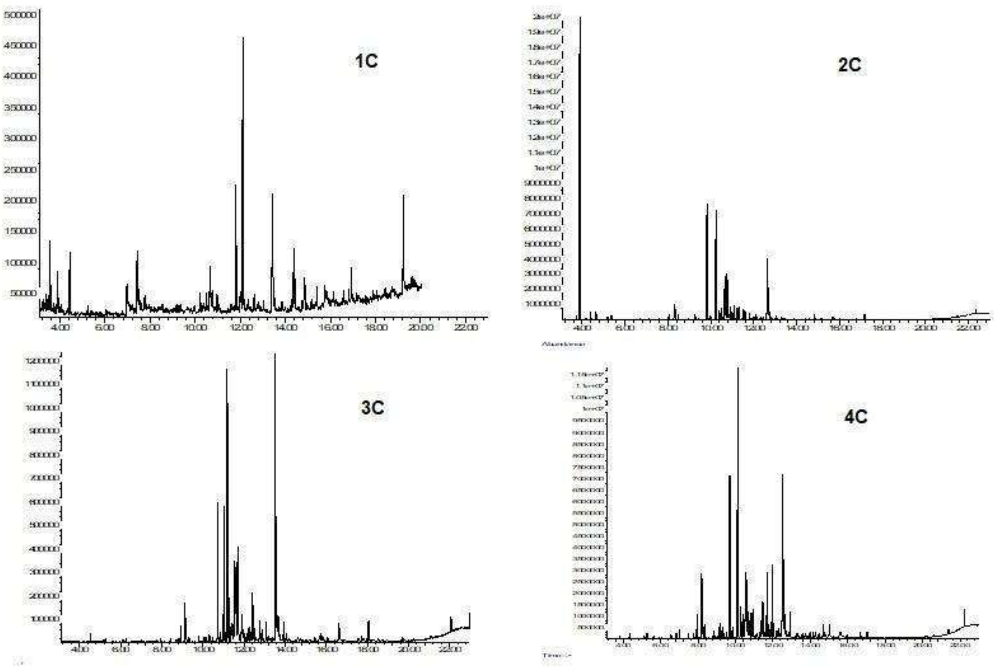


**Figure S1:** Chromatograms obtained by gas chromatography coupled with mass spectrometry (GC–MS) of the essential oil extracted from the **cones** of the Cascade variety of *Humulus lupulus* over the four cultivation cycles **1C**: 1st Cultivation (November 2022 to January 2023); **2C**: 2nd Cultivation (February–April 2023); **3C**: 3rd Cultivation: (September–December 2023) and **4C**: 4th Cultivation (January–May 2024).

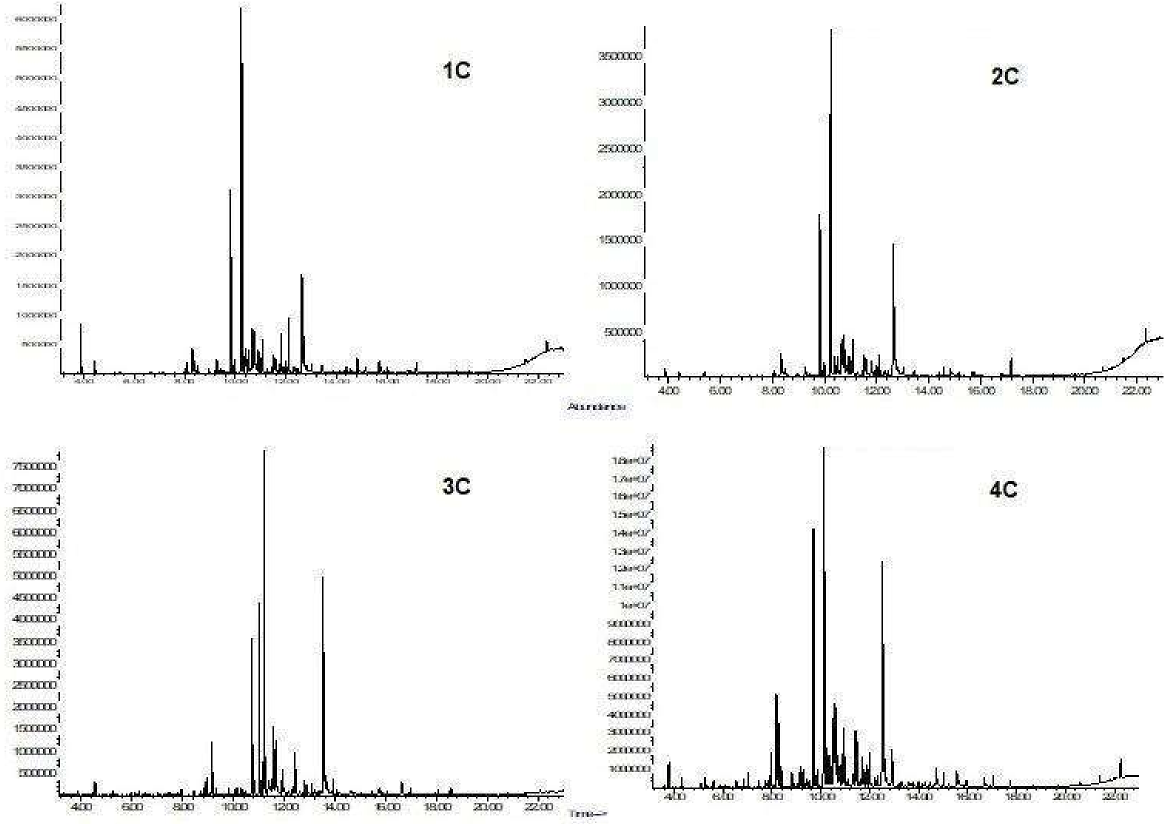


**Figure S2:** Chromatograms obtained by gas chromatography coupled with mass spectrometry (GC–MS) of the essential oil extracted from the **cones** of the Columbus variety of *Humulus lupulus* over the four cultivation cycles. **1C**: 1st Cultivation (November 2022 to January 2023); **2C**: 2nd Cultivation (February–April 2023); **3C**: 3rd Cultivation: (September–December 2023) and **4C**: 4th Cultivation (January–May 2024).

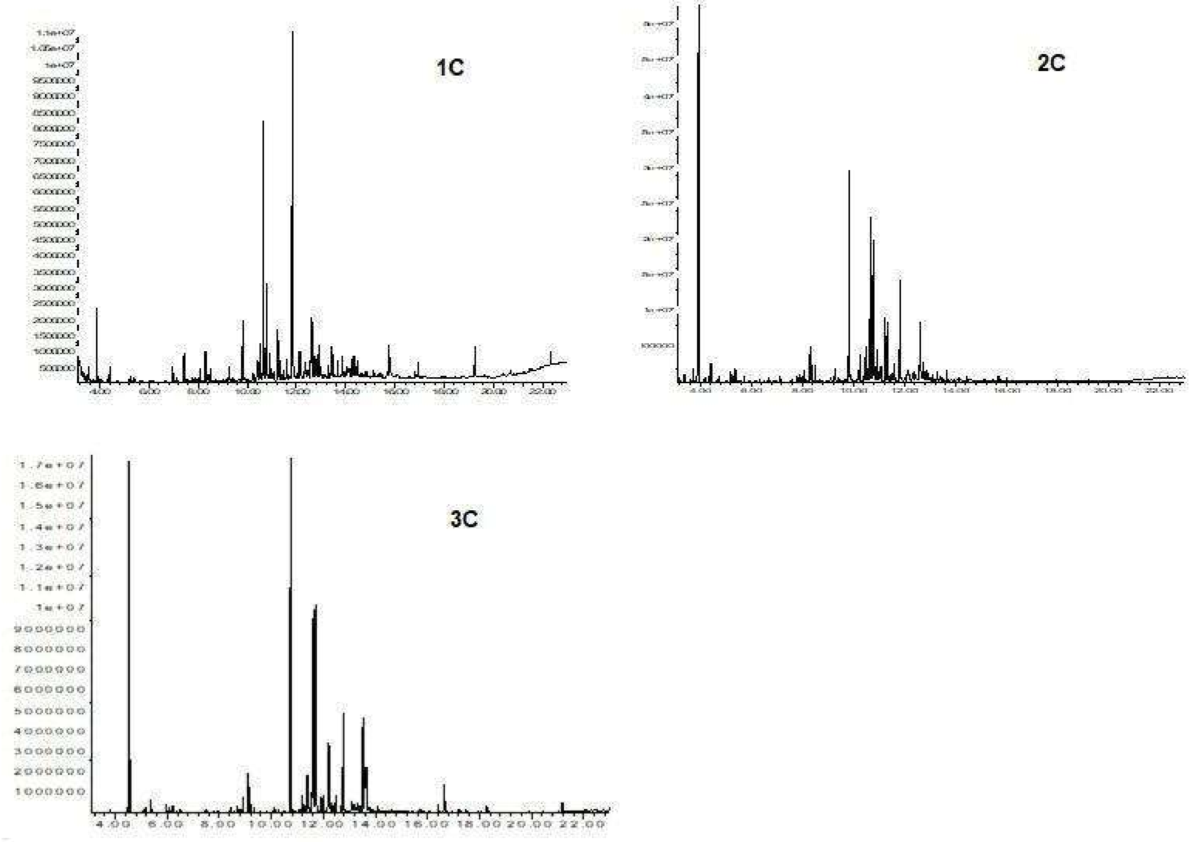


**Figure S3**: Chromatograms obtained by gas chromatography coupled with mass spectrometry (GC–MS) of the essential oil extracted from the **cones** of the Comet variety of *Humulus lupulus* over the four cultivation cycles. **1C**: 1st Cultivation (November 2022 to January 2023); **2C**: 2nd Cultivation (February–April 2023); **3C**: 3rd Cultivation: (September–December 2023) and **4C:** Absent.

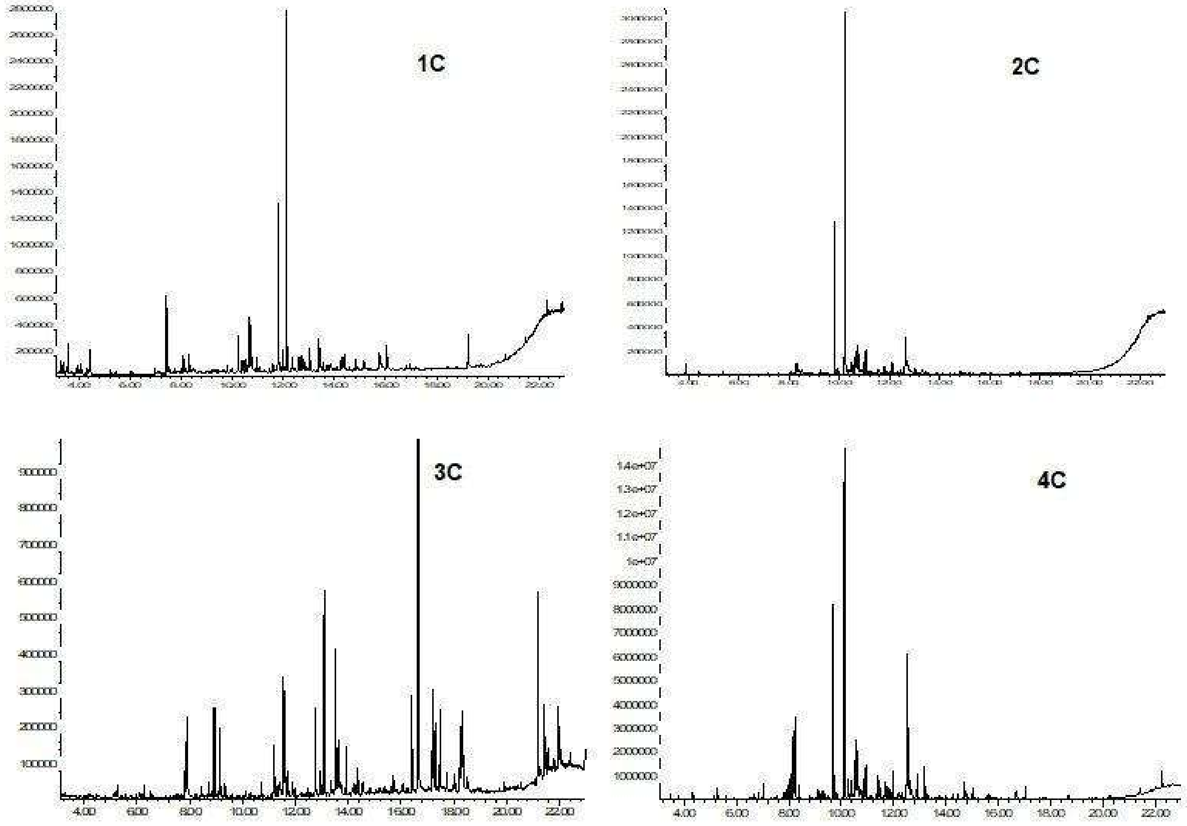


**Figure S4:** Chromatograms obtained by gas chromatography coupled with mass spectrometry (GC–MS) of the essential oil extracted from the **cones** of the Nugget variety of *Humulus lupulus* over the four cultivation cycles. **1C**: 1st Cultivation (November 2022 to January 2023); **2C**: 2nd Cultivation (February–April 2023); **3C**: 3rd Cultivation: (September–December 2023) and **4C**: 4th Cultivation (January–May 2024).

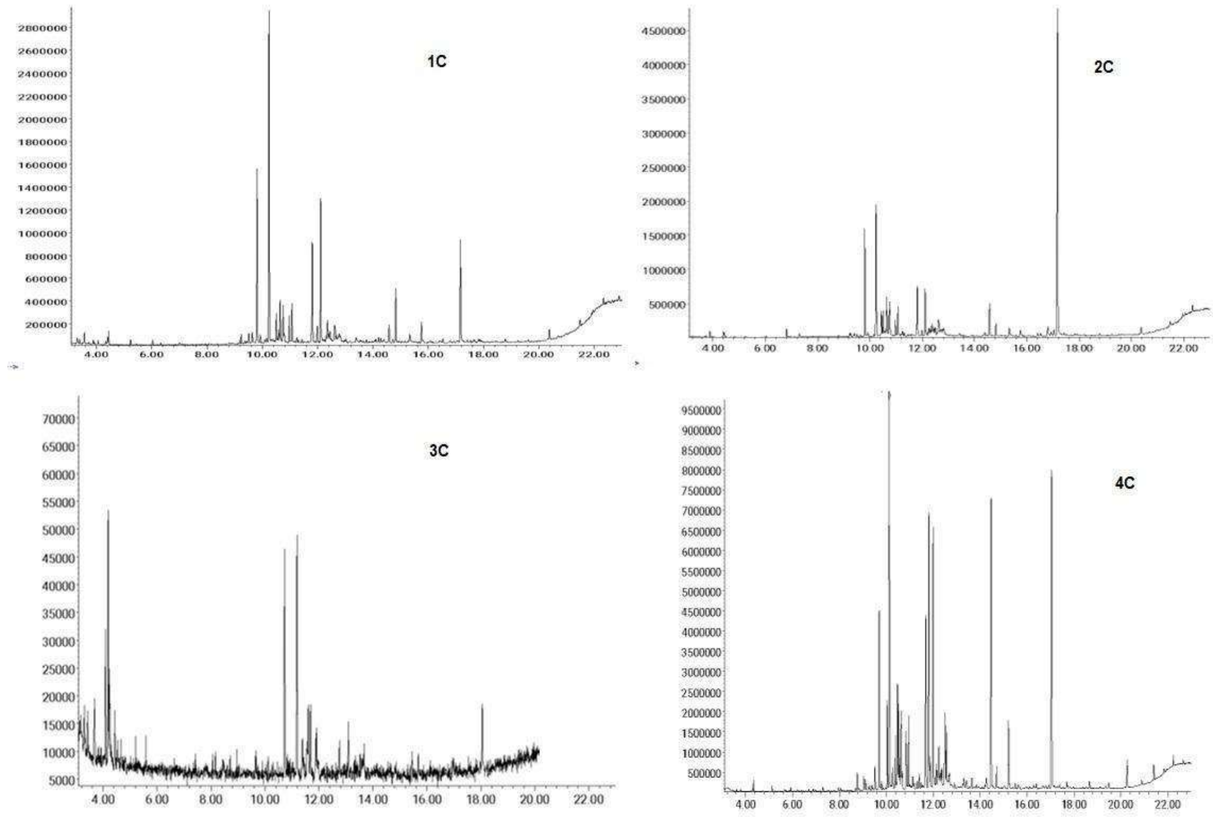


**Figure S5:** Chromatograms obtained by gas chromatography coupled with mass spectrometry (GC–MS) of the essential oil extracted from the **leaves** of the Cascade variety of *Humulus lupulus* over the four cultivation cycles. **1C**: 1st Cultivation (November 2022 to January 2023); **2C**: 2nd Cultivation (February–April 2023); **3C**: 3rd Cultivation: (September–December 2023) and **4C**: 4th Cultivation (January–May 2024).

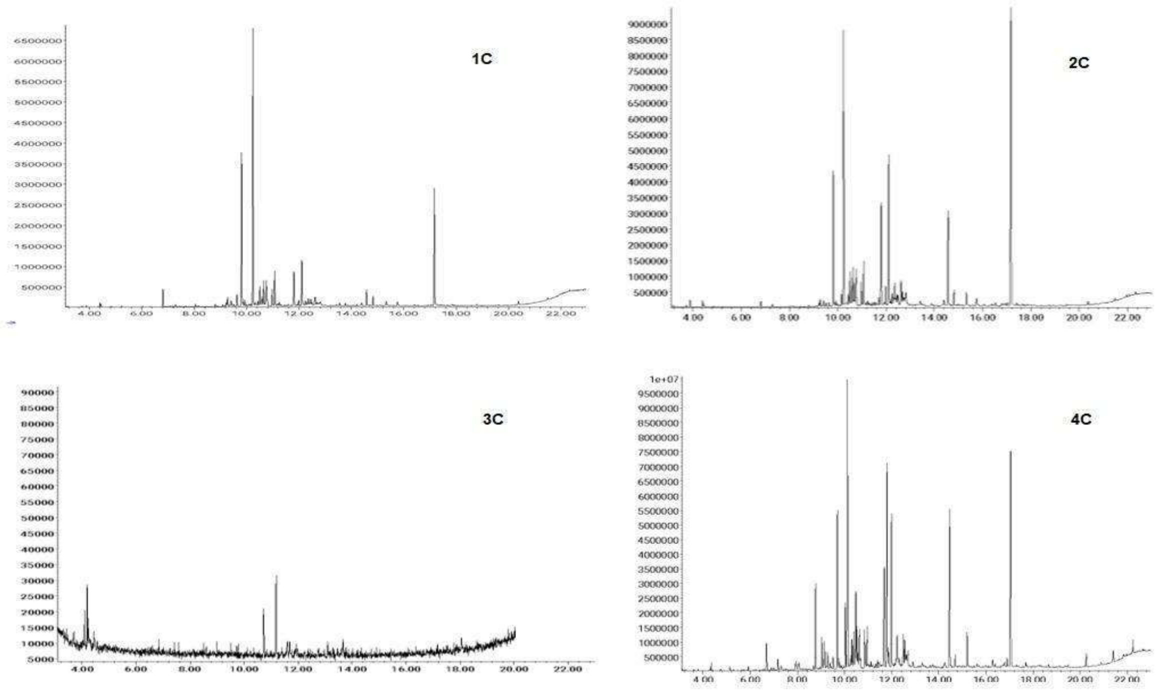


**Figure S6**: Chromatograms obtained by gas chromatography coupled with mass spectrometry (GC–MS) of the essential oil extracted from the **leaves** of the Columbus variety of *Humulus lupulus* over the four cultivation cycles. **1C**: 1st Cultivation (November 2022 to January 2023); **2C**: 2nd Cultivation (February–April 2023); **3C**: 3rd Cultivation: (September–December 2023) and **4C**: 4th Cultivation (January–May 2024).

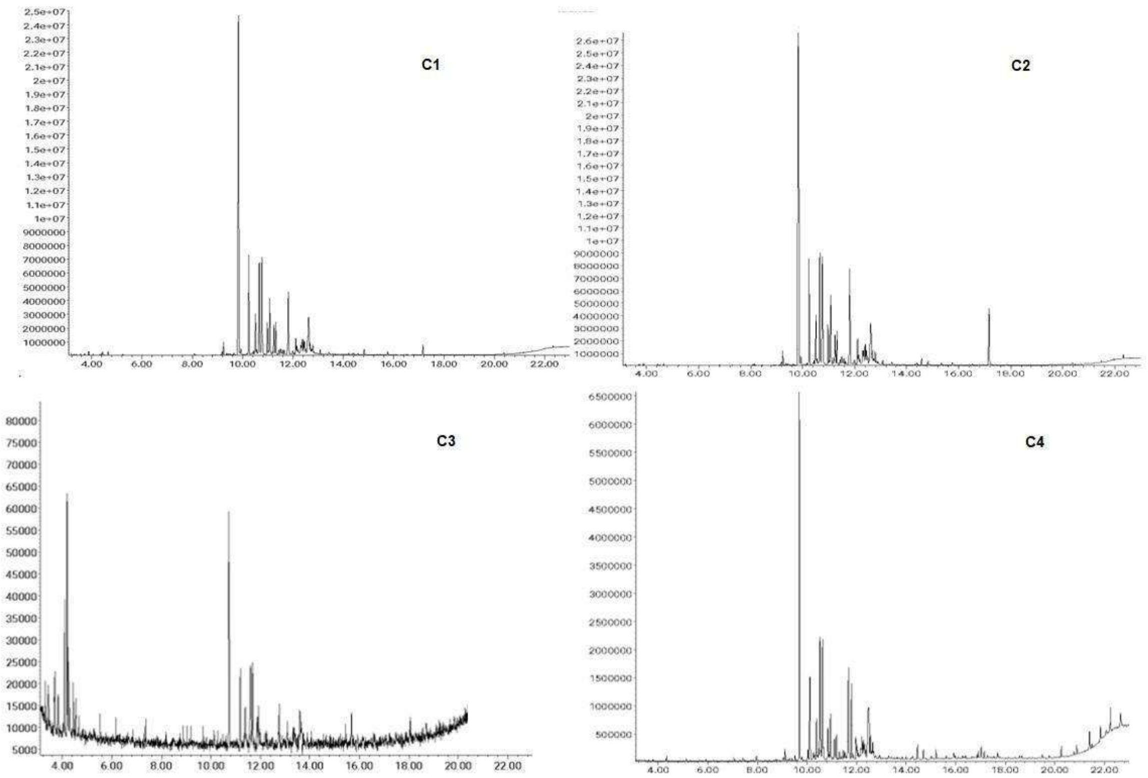


**Figure S7**: Chromatograms obtained by gas chromatography coupled with mass spectrometry (GC–MS) of the essential oil extracted from the **leaves** of the Comet variety of *Humulus lupulus* over the four cultivation cycles. **1C**: 1st Cultivation (November 2022 to January 2023); **2C**: 2nd Cultivation (February–April 2023); **3C**: 3rd Cultivation: (September–December 2023) and **4C**: 4th Cultivation (January–May 2024).

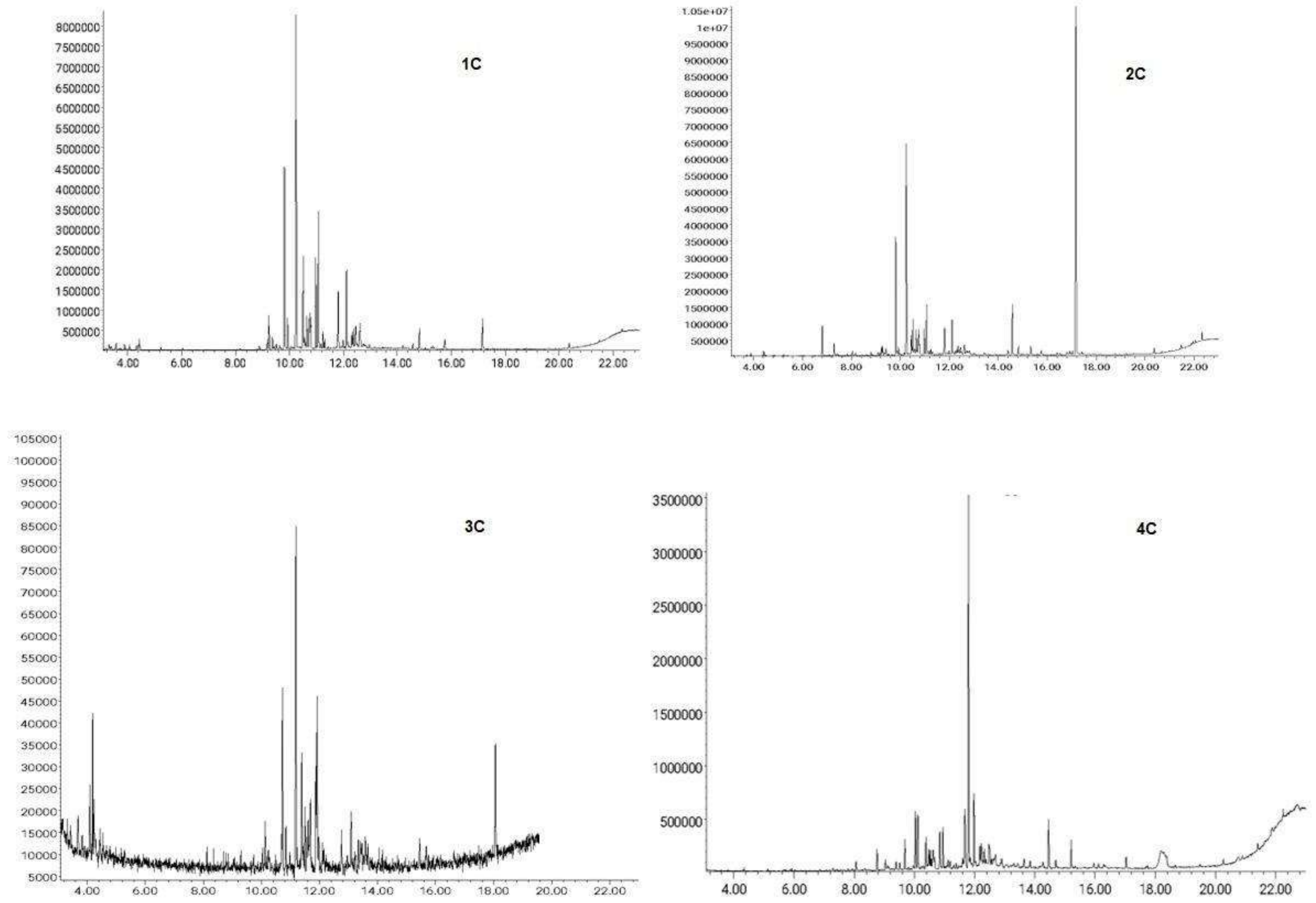


**Figure S8**: Chromatograms obtained by gas chromatography coupled with mass spectrometry (GC–MS) of the essential oil extracted from the **leaves** of the Nugget variety of *Humulus lupulus* over the four cultivation cycles. **1C**: 1st Cultivation (November 2022 to January 2023); **2C**: 2nd Cultivation (February–April 2023); **3C**: 3rd Cultivation: (September–December 2023) and **4C**: 4th Cultivation (January–May 2024).
